# Comparative Sequence Analysis of the *Ghd7* Orthologous Regions Revealed Movement of *Ghd7* in the Grass Genomes

**DOI:** 10.1371/journal.pone.0050236

**Published:** 2012-11-21

**Authors:** Lu Yang, Tieyan Liu, Bo Li, Yi Sui, Jinfeng Chen, Jinfeng Shi, Rod A. Wing, Mingsheng Chen

**Affiliations:** 1 State Key Laboratory of Plant Genomics, Institute of Genetics and Developmental Biology, Chinese Academy of Science, Beijing, China; 2 Arizona Genomics Institute, School of Plant Sciences, BIO5 Institute, University of Arizona, Tucson, Arizona, United States of America; National Rice Research Center, United States of America

## Abstract

*Ghd7* is an important rice gene that has a major effect on several agronomic traits, including yield. To reveal the origin of *Ghd7* and sequence evolution of this locus, we performed a comparative sequence analysis of the *Ghd7* orthologous regions from ten diploid *Oryza* species, *Brachypodium distachyon*, sorghum and maize. Sequence analysis demonstrated high gene collinearity across the genus *Oryza* and a disruption of collinearity among non*-Oryza* species. In particular, *Ghd7* was not present in orthologous positions except in *Oryza* species. The *Ghd7* regions were found to have low gene densities and high contents of repetitive elements, and that the sizes of orthologous regions varied tremendously. The large transposable element contents resulted in a high frequency of pseudogenization and gene movement events surrounding the *Ghd7* loci. Annotation information and cytological experiments have indicated that *Ghd7* is a heterochromatic gene. *Ghd7* orthologs were identified in *B. distachyon*, sorghum and maize by phylogenetic analysis; however, the positions of orthologous genes differed dramatically as a consequence of gene movements in grasses. Rather, we identified sequence remnants of gene movement of *Ghd7* mediated by illegitimate recombination in the *B. distachyon* genome.

## Introduction

Comparative genomics is a powerful tool to study gene and genome evolution [1]. However, studies based on model genomes are largely insufficient to interpret the evolutionary history and mechanism of genomic changes. The genus *Oryza* provides a fantastic model to study gene and genome evolution with its well defined phylogenic relationships and rich genomic resources available [2]–[6]. Comparative genomics in *Oryza* have provided insights into genome evolution [7]–[9], genome size variation [10], [11] and dynamics of gene evolution, such as lineage specific gene deletions, repeat-mediated gene movements and *de novo* gene formation [4], [12]–[14].

Recently, whole genome sequences of several grass species have provided us insights into genome conservation and lineage-specific features [15]–[17]. Exceptions to gene collinearity have also been observed frequently [1], [18]–[20]. Comparisons between *Oryza sativa* (rice) and *Brachypodium distachyon* indicate ∼18% of genes are absent in collinear blocks; this value rises to ∼43% when comparing rice and *Sorghum bicolor* (sorghum) [16], [21]. Erosion of gene collinearity can be explained by gene movement events [18], [22]. However, there were few reported cases for movements of agronomically important genes [13], [18], [23], [24].

The genus *Oryza*, together with several completely sequenced grass species, is becoming a powerful system to elucidate the evolutionary origin of agronomically important genes. *Ghd7*, a CCT domain-containing gene located on the short arm of rice chromosome 7, controls the number of grains per panicle, plant height and heading date [25]. Enhanced expression of *Ghd7* under long-day conditions plays a central role in the photoperiod pathway of flowering. The reduced function of *Ghd7* is associated with adaptation of rice to regions with low temperatures and short growth seasons [25]. *Ghd7* is thought to be an evolutionarily new gene, because it does not have homologs in *Arabidopsis thaliana*, and the protein sequence lacks a B-box domain, and the non-CCT portion differs from other CCT domain-containing proteins [25], [26]. Interestingly, comparative sequence analysis among rice, sorghum and maize indicated that *Ghd7* was absent from orthologous regions in the Andropogoneae lineage [27]. In order to uncover the evolutionary history of the *Ghd7* locus, we performed a comparative sequence analysis of the *Ghd7* orthologous regions in ten diploid *Oryza* species and related regions from *B. distachyon*, sorghum and *Zea mays* (maize). The *Ghd7* regions showed distinctive heterochromatic features compared to previously analyzed euchromatic regions (*Adh1*, *Moc1*, *Hd1*) in the genus *Oryza* [4], [13], [14]. The evolutionary history of *Ghd7* and the mechanism of gene movements were interpreted and discussed.

## Results

### Identification and Sequencing of BAC Clones of the *Ghd7* Orthologous Regions from the Genus *Oryza*

BAC clones covering the *Ghd7* orthologous regions were isolated from *Oryza rufipogon* (AA), *Oryza nivara* (AA), *Oryza glaberrima* (AA), *Oryza glumaepatula* (AA), *Oryza punctata* (BB), *Oryza officinalis* (CC), *Oryza australiensis* (EE), and *Oryza brachyantha* (FF). Thirty-three BAC clones were sequenced using Illumina Genome Analyzer II and Roche/454 Genome Sequencer (Table S1). In total, we generated ∼5.83 Mb of DNA sequence, representing 3.86 Mb of the corresponding *Ghd7* orthologous regions (Table S1). Having additional data points from the syntenic regions of non-*Oryza* species would be instrumental in reconstructing the evolution history of duplication, retention and syntenic gene order. Therefore, we also included the corresponding orthologous regions from O. *sativa* L. ssp. *japonica* (*japonica*), O. *sativa* L. ssp. *indica* (*indica*), B. *distachyon*, sorghum and maize for data analysis. A total of ∼7.6 Mb of genome sequence data were annotated (Table S2).

### Gene Organization of the *Ghd7* Regions

The *Ghd7* region from *japonica* was used as a reference for all comparative analyses. Genes were reannotated as described in the experimental procedures. Twenty gene models and two pseudogenes were annotated in the 553 kb region (Tables S3, S4). The intron/exon structures of the annotated genes were corrected according to the full-length cDNA or EST sequences (Table S3). A total of 163 genes were annotated in other *Oryza* species, *B. distachyon*, sorghum and maize (Table S2). Genes from each species are denoted by the abbreviation of each species: J, *japonica*; I, *indica*; GLA, *O. glaberrima*; RUF, *O. rufipogon*; NIV, *O. nivara*; GLU, *O. glumaepatula*; P, *O. punctata*; O, *O. officinalis*; A, *O. australiensis*; B, *O. brachyantha*; BD, *B. distachyon*; SB, *S. bicolor*; ZM, *Z. mays* (Tables S5, S6, S7, S8).

The gene densities of the *Ghd7*-surrounding regions were much lower than the *Moc1*, *Adh1* and *Hd1* regions [4], [13], [14], ranging from 9 kb/gene in *B. distachyon* to 110 kb/gene in *O. officinalis* (Figure S2 and Table S2). Eleven pseudogenes were annotated (Figure S1). The pseudogenes were caused by the insertion of repetitive elements (J-19, GLA-19, GLU-19, SB-6 and ZM-15), premature terminations (J-2, I-2, I-17, GLA-8 and GLA-18) and mutation at a splice site and the initiation codon (GLU-17) (Figure S1). Most of the pseudogenes were detected as duplicated genes or lineage-specific genes. However, three conserved genes were also observed to be pseudogenized in *O. glaberrima* (GLA-8), sorghum (SB-6) and maize (ZM-15). In addition, six gene models were observed to have variations in intron/exon structures in *Oryza* species, and their structures were confirmed by RT-PCR experiments (P-9, P-11, A-11, B-9, B-11 and B-22); the gene structures of 11 predicted gene models in the non-*Oryza* species were found to differ from their orthologs in *japonica* (Figure S1).

### High Gene Collinearity in the Diploid *Oryza* Species and a Loss of Conservation in *B. distachyon*, Sorghum and Maize

**Figure 1:**
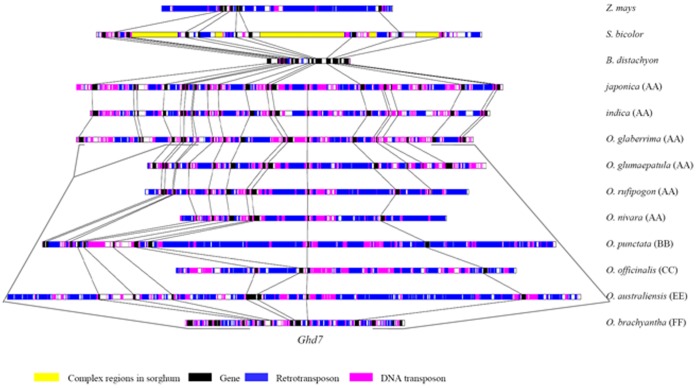
Synteny of the *Ghd7* orthologous regions in *Oryza* species, *B. distachyon*, sorghum and maize. Lines connect orthologous genes.

A comparison of the *Ghd7* orthologous regions indicated high gene collinearity within the genus *Oryza* and a loss of sequence conservation in *B. distachyon*, sorghum and maize (Figure 1, Tables S6, S7, S8). *Indica* has the highest level of sequence identity with *japonica*, differing mainly by the insertion or deletion of several repetitive elements. Even within the AA genomes, *O. glaberrima* and *O. glumaepatula* contain lineage-specific genes or pseudogenes. The core gene *Ghd7* was present in the diploid *Oryza* species only, but absent in the syntenic regions in *B. distachyon*, sorghum and maize. There were three pairs of tandem gene duplicates in *japonica* (Figure S1). Interestingly, these duplicated genes showed dramatic changes in distal species compared to rice, including copy number, the presence or absence of pseudogenes, gene structure and the direction of expression. In particular, one copy of a duplicated gene pair (J-18, I-18, GLA-18, GLU-18) contains a single exon and no introns, suggesting this duplicated gene copy may arise by reverse transcription of processed mRNA, with subsequent integration into the genome.

The sizes of the *Ghd7* orthologous regions varied tremendously among rice, *B. distachyon*, sorghum and maize. As shown in Figure 1 and Figure S1, *B. distachyon*, sorghum and maize only contained approximately half of the 22 genes or pseudogenes in *japonica*. The genome size of *B. distachyon* (∼272 Mb) [15] was smaller than that of rice (∼400 Mb) [28], and the corresponding orthologous region (107 kb) in *B. distachyon* was approximately 20% of the size of the rice syntenic region (553 kb). Annotation of the syntenic regions indicated that the reduced size was associated with the absence of ten genes, the high gene density (8.92 kb/gene) and low transposable element (TE) content (6.81%) in *B. distachyon*.

The genome size of maize is ∼2400 Mb [29], much larger than that of rice; however, the *Ghd7* orthologous region of maize (230 kb) is ∼40% of rice (553 kb). The eight annotated genes/gene fragments present in maize were divided into three gene islands surrounded by blocks of repetitive sequences. Many of the intergenic retrotransposon blocks were nested insertions. The factors contributing to the unexpectedly short sequence in maize differed from *B. distachyon*: 13 genes were absent and a high content of repetitive elements (78.04%) were observed, especially LTR retrotransposons.

Large regions of non-conservation were observed between the sorghum and rice *Ghd7* orthologous regions. The genome size of sorghum is ∼730 Mb [16]; however, the *Ghd7* orthologous region is ∼1.91 Mb. The five complex regions divided the sorghum region into six parts (Figure 1). A series of tandem duplicated genes were identified in the five complex regions, and many of them are related to biotic and abiotic stress responses, such as F-box genes (Table S7).

### Abundance and Variation of Transposable Elements Contributed to the Complexity of the *Ghd7* Orthologous Regions

TEs were annotated as described in the experimental procedures (Tables 1 and S9, S10, S11). The *Ghd7* orthologous regions showed a much higher levels of RNA TEs compared to the regions analyzed previously (Table S13), with only one exception of lower RNA TE content in *B. distachyon* (∼6%). Most of the RNA TEs are LTR retrotransposons (Table 1). The average insertion time of LTR retrotransposons from each species indicated that these LTRs spread after speciation (Table S10). In addition to the insertions, LTR retrotransposons were removed through illegitimate recombination (IR) and unequal homologous recombination (UR, converting LTR retrotransposons into solo-LTRs) in these orthologous regions [30]–[33]. However, we observed that the removal efficiency was not sufficient to counter the expansion caused by LTR retrotransposons in the *Ghd7* orthologous regions in *O. rufipogon*, *O. punctata*, sorghum and maize (Table 2).

**Table 1:** Composition and sequence contribution of TEs within *Ghd7* regions in *Oryza* species, *B. distachyon*, *S. bicolor* and *Z. mays*.

	JAA	IAA	GLAAA	GLUAA	RUFAA	NIVAA	PBB	OCC	AEE	BFF	BD	SB	ZM
Class I													
-LTR													
–Ty1/Copia	7.39	7.67	6.4	4.24	8.51	6.45	5.96	5.5	10.54	8.47	5.06	7.97	36.89
–Ty3/Gypsy	23.37	24.04	21.93	33.83	44.69	45.52	50.46	26.91	35.72	12.39	0.12	20.44	35.38
-Intact LTR	18.31	7.97	12.06	9.4	12.83	10.38	16.67	5.26	9.18	0	0	19.4	36.12
-Non-LTR													
–LINE	1.15	0.85	1.24	0.75	0.68	0.82	0.75	0.19	0.11	0.48	0.84	2.94	0.25
–SINE	0.54	0.54	0.62	0.3	0.25	0.27	0.06	0.2	0.03	0.13	0	0.035	0
-Intact Non-LTR	0.3	0.28	0.29	0	0.13	0.1	0	0.12	0	0	0	0.02	0
Total (%)	33.62	33.22	30.3	39.42	54.27	53.23	58.33	39.27	48.02	22.47	6.02	33.1	72.88
Class II													
–MITE/Tourist	1.62	1.51	1.51	1.7	1.24	1.14	0.69	1.05	0.13	1.98	0.65	0.802	1.18
-MITE/Stowaway	1.53	1.35	1.69	1.29	1.18	1.03	0	0.48	0.16	2.11	0	0.37	0
–hAT	0.94	0.91	1.02	0.4	0.29	0.06	0.08	0.11	0.75	0.51	0.14	0.67	0.58
–En-Spm	6.74	3.87	4.3	5.74	6.56	7.51	8.58	13.71	8.81	0.13	0	13.82	0.92
–MuDR-IS905	2.5	2.31	2.2	2.38	1.36	1.44	1.64	5.99	0.59	1.12	0	0.112	2.08
–Unclassified	1.98	1.71	1.91	1.8	0.86	1.31	0.64	0.29	0.77	1.18	0	0.796	0.4
-Intact DNA TE	10.48	6.01	7.55	9.05	7.02	9.02	3.49	11.17	9.53	2.04	0	6.03	1.05
Total (%)	15.31	11.66	12.63	13.31	11.49	12.49	11.63	21.63	11.21	7.03	0.79	16.57	5.16

**Table 2:** The number of different categories of RNA TEs presents in *Ghd7* orthologous regions.

	JAA	IAA	GLAAA	RUFAA	GLUAA	NIVAA	PBB	OCC	AEE	BFF	BD	SB	ZM	Mechanism
Intact LTR	9	7	7	12	5	8	14	2	9	0	1	44	19	
Solo w/TSD	16	13	14	10	10	8	12	7	10	2	0	5	2	UR(Intra)
Solo w/o TSD	3	4	3	3	3	3	1	5	1	0	0	0	3	UR (Inter)
Intact LTR w/o TSD	1	0	2	0	0	3	2	0	0	1	0	9	1	UR(Intra)
Truncated Solo	15	19	10	19	11	19	32	26	30	15	0	15	18	UR(Inter)or UR(Intra)&IR
3' LTR deleted	1	5	3	6	7	4	11	6	10	1	0	1	11	IR
5' LTR deleted	4	7	1	6	4	4	9	1	17	1	0	1	5	IR
5' and/or 3' partially deleted	4	3	9	13	8	7	22	14	15	7	1	10	8	IR
Recombination complex	1	3	2	2	2	3	6	3	9	0	0	1	8	UR (Inter)
Tendency^a^	C	C	C	E	C	C	E	C	C	C	E	E	E	

a C means contraction and E means expansion.

### *Ghd7* is a Heterochromatic Gene in Rice

The *Gypsy* retrotransposon content of the *Ghd7* orthologous regions was greater than *Copia*, especially in *O. rufipogon*, *O. nivara* and *O. punctata* (Table S12). However, this difference was not observed in *B. distachyon* or maize, suggesting the *Ghd7* regions in different species may be located in distinct chromatin environments (Table S12). From our annotation information, the *Ghd7* orthologous regions in most diploid *Oryza* species display features of heterochromatin compared to other well-characterized euchromatic regions (Figure S2, Tables S12 and S13). Previous cytological studies indicated that the *Ghd7* locus in chromosome 7 of rice is likely to be sited within a high density, or at least a moderately condensed, region of chromatin [34], [35]. Furthermore, strict recombination suppression was detected in the *Ghd7* region from QTL analysis in rice [25]. All of these results suggested that *Ghd7* might be a heterochromatic gene in the genus *Oryza* [36]–[38].

**Figure 2:**
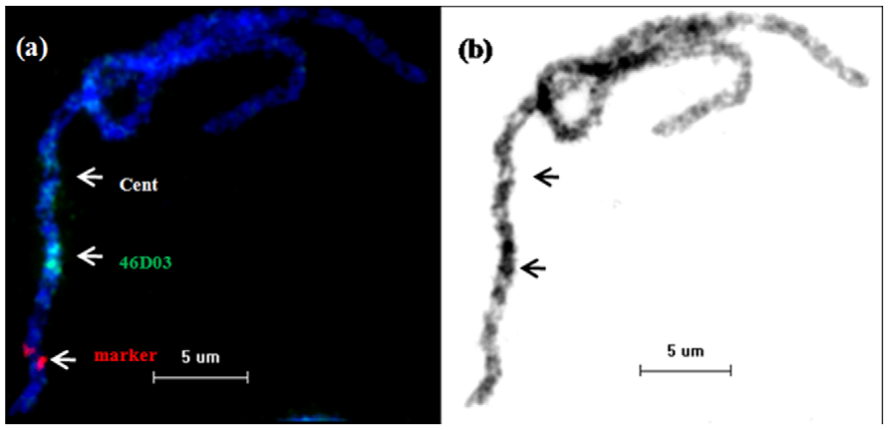
Physical mapping of PAC 46D03 by pachytene chromosome FISH in *japonica*. (a) The PAC 46D03 (green signal) is mapped to the heterochromatic region on the short arm of chromosome 7; “Cent” indicates the position of the centromere. The red signal is the marker for chromosome 7. (b) Inverted grayscale image of the same chromosome in (a). The black portion represents the heterochromatic region of chromosome 7 in rice.

To confirm this possibility, we performed a fluorescence-in-situ-hybridization (FISH) experiment using the PAC (P1 artificial chromosome) clone 46D03, which contains the *Ghd7* gene, as a probe. The result indicated that the PAC clone was located in a deeply stained region on the short arm of chromosome 7 of rice (Figure 2); indicating *Ghd7* is a heterochromatic gene in rice.

### The Evolutionary History of *Ghd7* in the Grass Genomes

**Figure 3:**
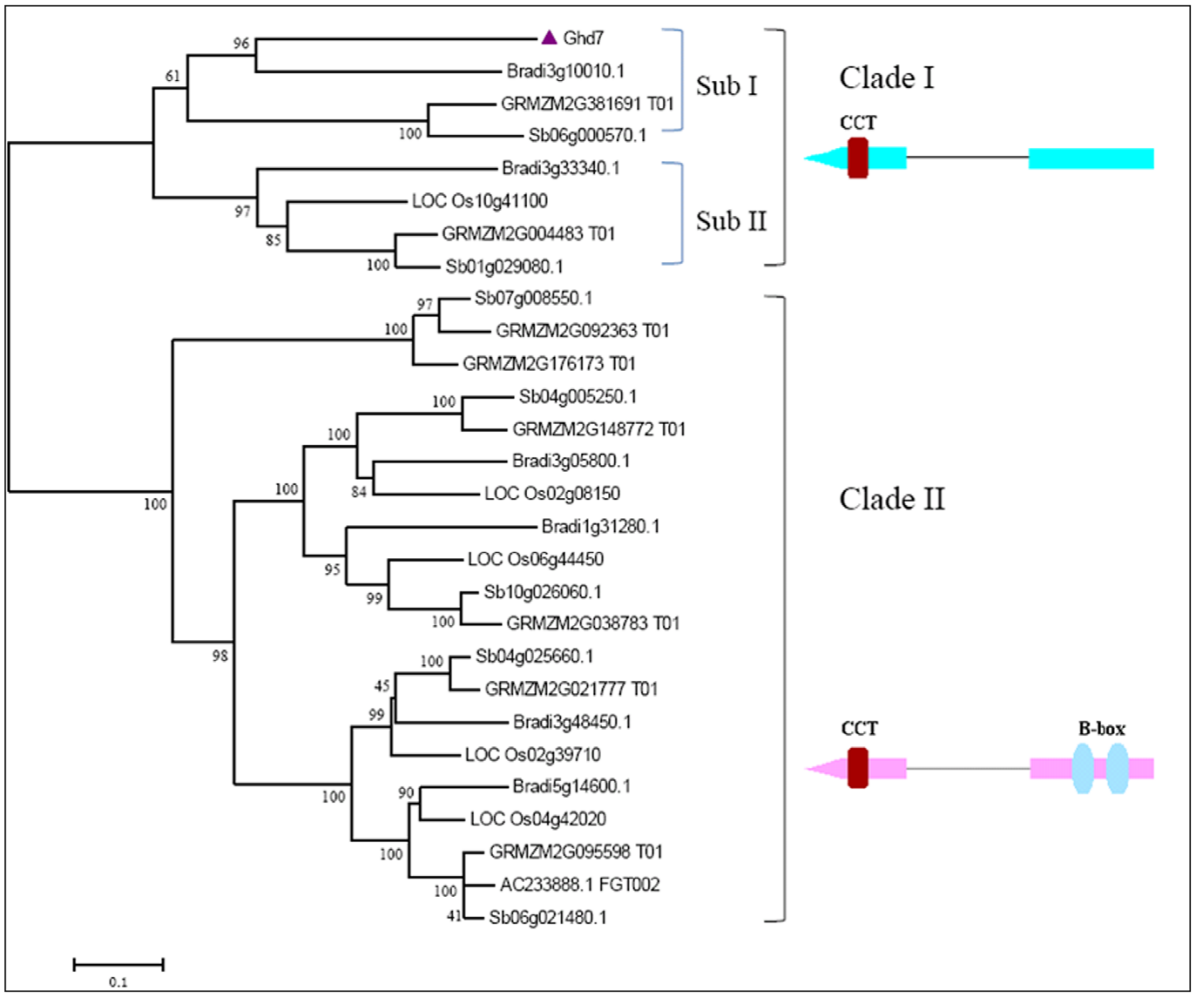
Unrooted phylogenetic tree for *Ghd7* and its homologous genes from rice, *B. distachyon*, sorghum and maize. The tree was built using the Neighbor-Joining method. The conserved motifs were shaded in different colors. The two clades can be distinguished by the motif distribution.

To trace the evolutionary history of *Ghd7*, the CCT domain-containing genes from rice, *B. distachyon*, sorghum and maize were identified by BLASTP based on homology to the *Ghd7* CCT domain. A total of 28 homologous genes were identified (Table S14). As shown in Figure 3, the CCT domain-containing genes from different species can be classified into two clades by phylogenetic analysis. SMART and Pfam analyses indicated that genes in Clade I contain a CCT domain only, similar to *Ghd7*. In contrast, both a B-box and a CCT domain were detected for genes in Clade II, typified by the CCT family gene *CO*.

The Clade I can be further divided into two subgroups, each containing four genes from rice, *B. distachyon*, sorghum and maize. In subgroup II, LOC_Os10g41100, Bradi3g33340, Sb01g029080 and GRMZM2G004483_P01 are orthologous genes within a syntenic region. LOC_Os10g41100 and *Ghd7* are duplicated genes in rice, and the gene duplication occurred prior to the divergence of rice, sorghum, maize and *Brachypodium*. In subgroup I, *Ghd7* (LOC_Os07g15770), Bradi3g10010, Sb06g000570 and GRMZM2G381691_T01 were also defined as gene orthologs, as they met the following criteria: they formed a monophyletic clade; systematic information within this clade conformed to a generally accepted species trees; the bootstrap value was >55% [39]. These results indicated that *Ghd7* has orthologs in non-*Oryza* species, but they are not located in syntenic regions (Table S15).

**Figure 4:**
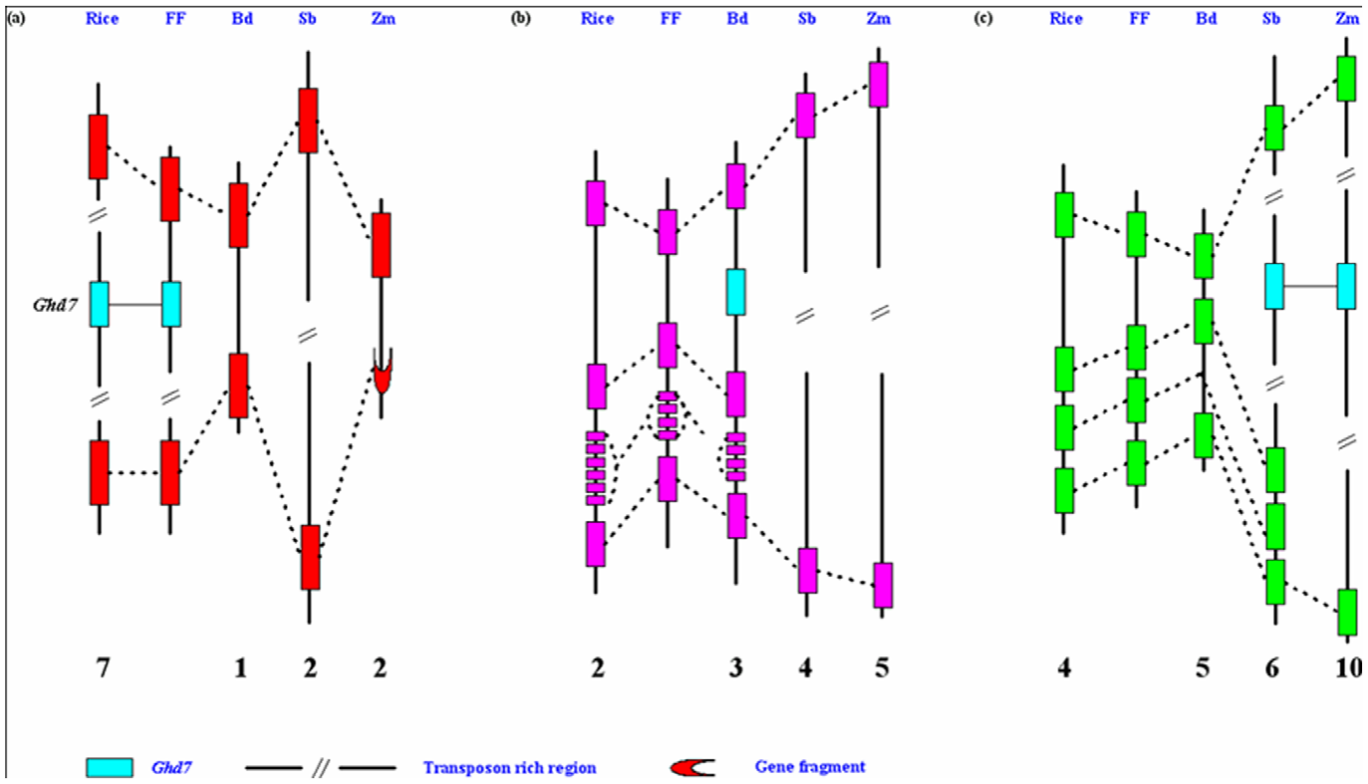
Comparative analysis of the corresponding orthologous regions from rice, *O. brachyantha*, *B. distachyon*, sorghum and maize. (a) the *Ghd7* orthologous regions; (b) the Bradi3g10010 orthologous regions; (c) the Sb06g000570 and GRMZM2G381691_T01 orthologous regions. FF: *O. brachyantha*; Bd: *B. distachyon*; Sb: *S. bicolor*; Zm: *Z. mays*. The number on the bottom depicts the chromosome number in each species.

To investigate the origins of *Ghd7* and its orthologs, we compared the corresponding orthologous regions from rice, *B. distachyon*, sorghum and maize. As a basal species of the genus *Oryza*, *O. brachyantha* (FF) had been subject to whole genome sequencing by our laboratory. To ensure a reasonable evolutionary gradient, the corresponding regions in *O. brachyantha* were included in the comparative analysis (Figure 4). Overall gene collinearity was observed in these genomic regions, but *Ghd7* and its orthologs are located in three different syntenic regions in *Oryza*, *B. distachyon* and the Andropogoneae lineages (Figure 4), indicating *Ghd7* was present prior to the divergence of rice, *B. distachyon*, sorghum and maize. Therefore, the *Ghd7* orthologs must have moved to their current positions by undefined mechanisms in some species.

### The Mechanisms for Movements of *Ghd7* and its Orthologs

A whole genome duplication event occurred ∼50–70 million years ago in grasses, prior to the divergence of major cereals [40], [41]. Many of the duplicated genes lost quickly after duplication by large-scale chromosomal rearrangements and deletions, leading to diploidization [42]–[45]. If the movement of *Ghd7* or its orthologs was resulted from the differential fractionation of duplicated genes derived by the whole genome duplication, *Ghd7* and its orthologs should be located on homoeologous chromosomes. Rice chromosome 3 is homoeologous to chromosome 7 where *Ghd7* resides. However, the *Ghd7* orthologs are located on chromosome 3 of *B. distachyon*, chromosome 6 of sorghum, and chromosome 10 of maize, which is homoeologous to chromosome 2 and chromosome 4 of rice, respectively (Figure 4), indicating *Ghd7* and its orthologs are not located on homoeologous chromosomes. In fact, the duplicated region of the *Ghd7* orthologous region could not be identified in rice at all, probably due to the heterochromatic features. Therefore, we conclude that the movements of *Ghd7* and/or its orthologs were not resulted from the fractionation of duplicated genes following the whole genome duplication in grasses.

**Figure 5:**
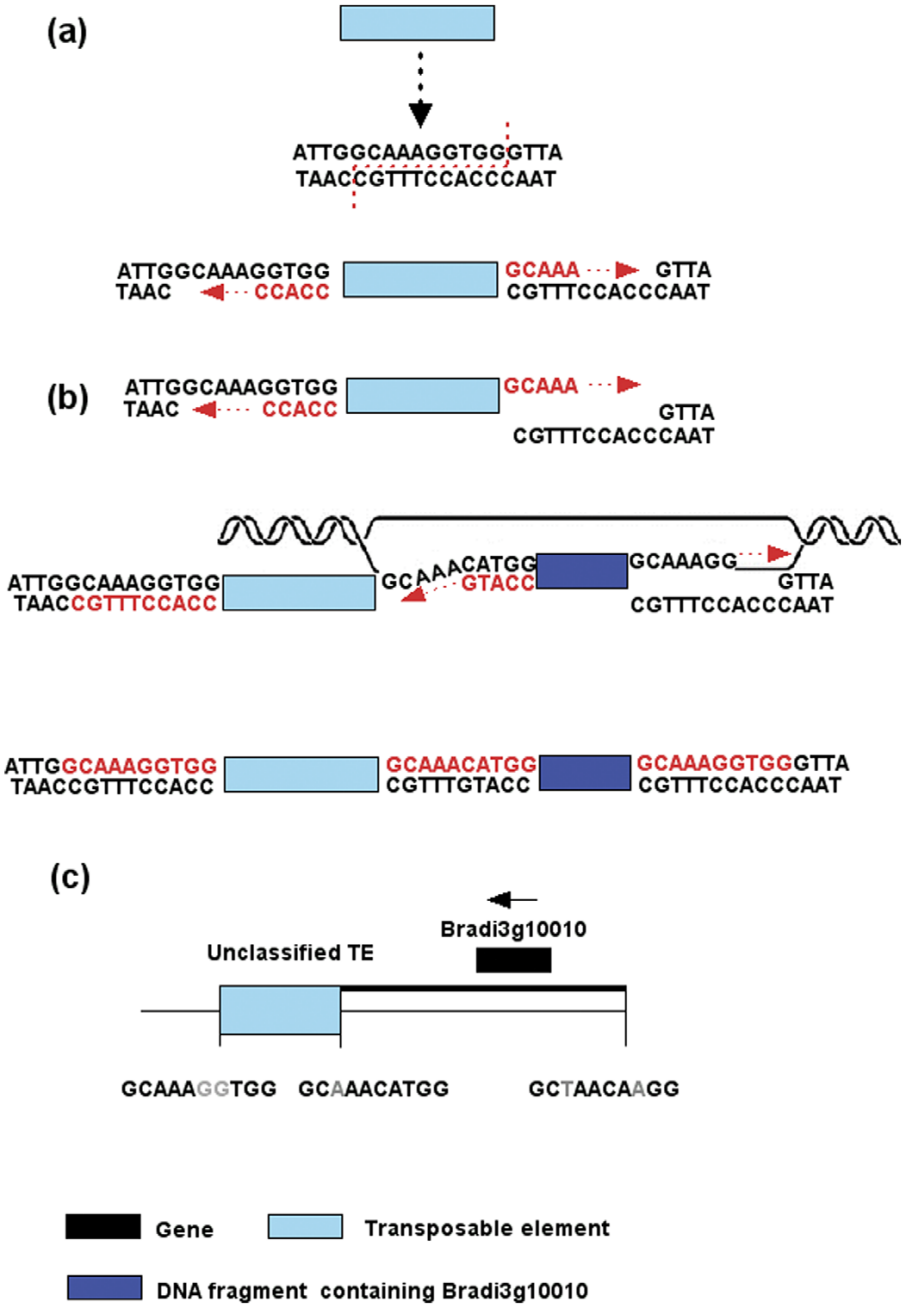
Molecular mechanism for the movement of Bradi3g10010. (a) An unclassified intact transposable element cleaves the host DNA for insertion and the formation of target site duplication. (b) The fragment containing Bradi3g10010 was used to fill the double-stranded break. (c) The model for the movement of Bradi3g10010. This figure was modified according to Wicker et al. 2010.

Based the model shown in Figure 5, we identified the evidence for the movement of Bradi3g10010, the *Ghd7* ortholog in *B. distachyon*. We modeled that a DSB was caused by insertion of an unknown transposable element, and a DNA fragment containing Bradi3g10010 was used to fill the gap. A 10 bp target site duplication flanking the unknown transposable element and one side of Bradi3g10010 were detected (Figure 5c). Unfortunately, we could not detect clear evidence of *Ghd7* movement in other species due to the high content of repetitive elements in the surrounding regions.

### Additional Gene Movements Mediated by DSB Repair in the *Ghd7* Orthologous Regions

**Figure 6:**
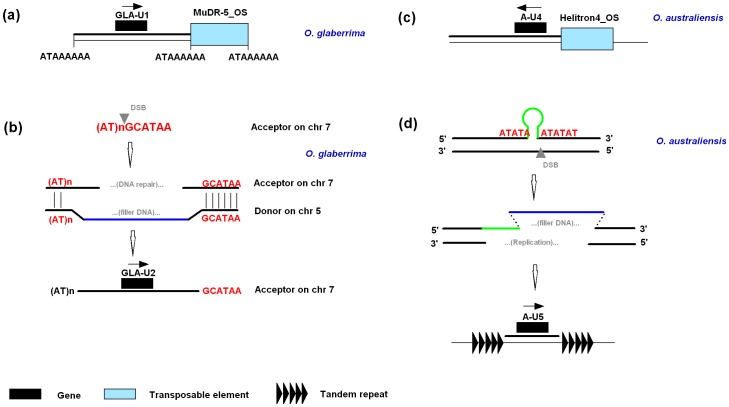
Examples of gene movements caused by DSB repair across the *Ghd7* orthologous regions. (a) The movement of GLA-U1 in *O. glaberrima* used a similar mechanism as Bradi3g10010 in *B. distachyon* (Figure 5). (b) The DSBs occur frequently in fragile sites, such as tandem repeats. A fragment containing GLA-U2 on chromosome 5 (donor) was used to fill the gap caused by DSB on chromosome 7 (acceptor) in *O. glaberrima*. The right borders for donor and acceptor are identical. (c) The fragment containing A-U4 might be captured by a *Helitron* element. (d) The A-U5 in *O. australiensis* is flanked by an array of tandem repeats on both sides. DSBs were possibly introduced during a template slippage of these tandem repeats, and the fragment containing A-U5 was used to fill the gap.

Four lineage-specific genes or gene fragments in the *Ghd7* orthologous regions have signatures of DSB repair (Figure 6). GLA-U1 in *O. glaberrima* and A-U4 in *O. australiensis* were hypothesized to be moved to their current positions by repair of DSBs created by insertion of MuDR-5_OS and HELITRON4_OS, respectively (Figure 6a and 6c). Tandem repeat motifs are hotspots for recombination [46]. GLA-U2 located on chromosome 7 of *O. glaberrima* (the acceptor) and its homolog LOC_Os05g34200 located on chromosome 5 was flanked by an identical 6-bp sequence signature on one side (Figure 6b). In addition, simple sequence repeats surrounding A-U5 in *O. australiensis* suggested that the A-U5 movement was mediated by either unequal crossing-over or template slippage (Figure 6d). In particular, the donor regions of GLA-U1and GLA-U2 were also observed.

### Gene Movements Mediated by Repeat Elements

The movements of four genes were found to be mediated by transposition of repeat elements (Figure S3). GLU-U7, A-U6 and B-U3 were embedded within different types of Pack-MULEs. GLU-U7, a GRF domain-containing gene, was evolved through capturing a gene fragment from elsewhere in the genome by *TNR12*/MuDR and inserted into the intron of pseudogene GLU-19. A-U6, a B-box-containing gene homologous to LOC_Os02g07930, moved to its current location by *MERMITEA*/MuDR. B-U3, which is located between B-8 and B-9 in *O. brachyantha*, was formed through transposition of OSTE1 carrying a DNA fragment from the second exon of LOC_Os10g34340. Finally, ZM7 moved to its current position by EnSpm-13_ZM, a CACTA family transposon in maize. Consistent with other studies, DNA TEs were found more frequently in capturing gene fragments through transposition [16], [17], [21].

## Discussion

More and more species have been fully sequenced and provided insight into genome structural organization and evolution. Comparative analyses have shown the extensive conservation of gene order, but the loss or gain of genes or genomic segments can be easily detected in closely related species and are important for genome organization and evolution. For instance, comparison of grass genomes indicated gene expansion in the evolution of grass-specific-genes [15]. However, the gene loss in duplicated regions were implicated in returning a paleopolyploid to a diploid state after whole-genome duplication [16], [17]. Gene movement is a specific type of gene gain by gain of genes or genomic segments in the acceptor sites, but seems like “movement” due to the subsequent gene loss in the donor sites. The loss and gain of gene or genomic segment usually happened during the cell cycles when recombination occurs at double-strand break via homologous recombination (HR) or non-homologous end joining (NHEJ) pathways [46]-[49]. The mechanism was found to be involved in many human diseases [50], [51]. In addition, gain of genes or genomic segments could also be mediated by transposable element capture or retroposition. However, the frequency of these mechanisms are much lower than recombination [21].

*Ghd7* is an agriculturally important gene that affects the number of grains per panicle, plant height and heading date. In our study, we found that *Ghd7* was conserved throughout the genus *Oryza*, but absent in orthologous positions in non-*Oryza* species. However, *Ghd7* was not deleted from the genome, but moved to different genomic locations in different subfamilies of grasses. In *B. distachyon*, features indicating *Ghd7* movement was detected. DNA double-strand break (DSB) repair can take place at any position in the genome and is another mechanism for gene movement. Wicker et al. (2010) proposed a model that the DSB could be repaired with a foreign DNA fragment containing a gene after template slippage or an unequal crossing-over event through synthesis-dependent strand annealing. As a result of a DSB, which was induced by an unknown DNA TE, a DNA fragment containing Bradi3g10010 was used as a template for DSB repair by microhomology or non-homologous end joining [52]. Unfortunately, footprints for movements of *Ghd7* in maize, sorghum, and foxtail millet have not been identified, possibly due to their being obscured by a high density of repetitive elements.

The whole genome analysis of rice and *B. distachyon* has indicated that “copy-and-paste” is the dominant duplication process for most non-collinear genes. The apparent movement of genes may result from subsequent deletions in the donor region [21]. The putative donor regions of *Ghd7* and its orthologs were not observed in the species analyzed, suggesting that the original copy in the donor region was deleted.

The *Ghd7* orthologs were identified in both short-day and long-day plants. The expressions of *Ghd7* orthologs were detected by RT-PCR experiments in eight *Oryza* species grown under long-day conditions (Figure S4). Similar to *Ghd7*, the *Ghd7* ortholog in maize (GRMZM2G381691_T01) was shown to regulate flowering time through determining photoperiod sensitivity [53]. We propose that the *Ghd7* orthologs in sorghum (Sb06g000570) and *Brachypodium* (Bradi3g10010) have similar functions in controlling flowering time. Thus, the *Ghd7* flowering pathway is not unique to rice; rather, it may regulate flowering properties in a wide range of grass species.

Heterochromatin is defined as densely coiled chromatin that generally replicates late during the S phase [38]. Low gene density, and large blocks of repetitive DNA, especially a high *Gypsy* content, are characteristics of heterochromatic regions [36]–[38]. *Gypsy* and *Copia*, which differ in the order of RT (reverse transcriptase) and INT (DDT integrase) in the POL, are two subfamilies of LTR retrotransposons [54]: *Gypsy* tends to insert into heterochromatin, while *Copia* inserts into euchromatin [55]. Most of the *Ghd7* orthologous regions in *Oryza* species displayed features of characteristic heterochromatin, especially in *O. rufipogon*, *O. nivara* and *O. punctata* (Figure S2, Tables S9 and S11). In contrast, the low TE content and high gene density is suggestive of a euchromatic environment for the *Ghd7* region of *O. brachyantha*. Heterochromatin is commonly characterized as silent chromatin; however, several hundreds of heterochromatic genes in *Drosophila*, plants and mammals are discovered recently and many of them have transcriptional activity [56]–[58]. In plants, agriculturally important heterochromatic genes have rarely been reported [59]–[61]. The expression of *Ghd7* was detected in diploid *Oryza* species under long-day condition, suggestive of its conserved activity in a heterochromatic environment.

In summary, the dramatic position-shift of *Ghd7* orthologs in the grass genomes and different allele distribution of *Ghd7* in rice indicated plasticity of this agronomically important gene. The mechanism for *Ghd7* movement in *B. distachyon* suggests that repetitive elements play an important role in gene and genome evolution in plants. Finally, as a heterochromatic gene, the regulation of *Ghd7* might be an interesting model to understand the effect of chromatin environment on gene regulation in plants.

## Materials and Methods

### Materials, Growth Conditions and Gene Structure Verification

The seed dormancy of diploid *Oryza* species was broken by heat treatment (50–54°C for five days). Seeds were washed thoroughly and germinated on moist filter paper in petri dishes at 37°C. Seedlings were transferred to pots and placed in greenhouse seven days later following the methods described by Xue *et al.* (2008): neutral day-length conditions (12 h sunlight/day) for 30 days; then long-day conditions (15 h sunlight/day) for ten days. Finally, leaves were harvested and stored at −80°C. Total RNA was extracted with TRIzol reagent (TIANGEN, Cat#DP405-02). The cDNA was synthesized by reverse transcriptase (Promega, Cat.M170A). The PCR conditions were as follows: 5 min at 94°C; 35 cycles of 30 sec at 94°C, 30 sec at suitable primers temperature and 1 min at 72°C; 8 min at 72°C. Amplified products were cloned into a T vector (Promega, Cat#1360) and sequences were verified using an ABI 3730 automated capillary sequencer (Applied Biosystems).

### Isolation of BAC Clones of *Ghd7* Orthologous Regions from Wild Rice

Ten OMAP BAC libraries (*O. nivara*, *O. rufipogon*, *O. glaberrima*, *O. glumaepatula*, *O. punctata*, *O. officinalis*, *O. australiensis*, *O. brachyantha*, *O. granulate* and *O. minuta*) from nine diploid and one tetraploid species were used to isolate BAC clones of the *Ghd7* orthologous regions. Primers for probes were designed for *Ghd7* and its flanking genes. Each probe was hybridized to the wild rice BAC filters using protocols described in Arizona Genomics Institute Website (http://www2.genome.arizona.edu/research/protocols_bacmanual). Unfortunately, none of the positive clones were isolated in *O. granulata* (GG) and *O. minuta* (BBCC) because of insufficient coverage of the BAC clones in the target region. Validated BACs were purified using a QIAGEN Large-Construct Kit (Cat.No.12462) and sequenced using the next generation sequencing technology.

### Sequencing the BAC Clones from the *Ghd7* Orthologous Regions

For each species, paired-end sequencing libraries were constructed with an insertion size of approximately 500 bp and sequenced on Illumina Genome Analyzer II. Because of the high content of repetitive elements, the assembled sequences resulted in many scaffolds without ordering information. Therefore, we used the Roche/454 Genome Sequencer FLX Instrument as a complementary method to sequence these low quality BAC clones. Overlaps between neighboring BACs were determined using BLASTN, and the resultant pseudomolecules were constructed after careful inspection and verification of each overlap. The sequences of all BACs used in this analysis were deposited to the GeneBank datalibrary under the following accession numbers [GeneBank: JN873128-JN873135]. The sequences and related CDS (coding sequence) databases of *japonica*, *indica*, *B. distachyon*, sorghum and maize were downloaded from individual websites (http://rice.plantbiology.msu.edu; http://rice.genomics.org.cn/rice/index2.jsp; http://www.brachypodium.org; http://www.phytozome.net/sorghum; http://www.maizegdb.org).

### Sequence Annotation of Protein-coding Genes and Transposable Elements

Sequences were annotated using the *ab initio* prediction programs FGENESH (http://www.softberry.com) for gene prediction [62]. In addition, candidate genes have to meet the following criteria: not transposon-related, and containing a known functional domain or having a homolog to known proteins or having a homolog at the syntenic position. All annotations were overlaid on individual BAC sequences and were visualized and edited using ACT v5 and Artemis [63]. The exon–intron structure of gene models were verified by aligning the genomic sequence with cDNAs or ESTs and experimental verification by sequencing RT-PCR products amplified using specific primers for each gene.

Transposable elements (TE) were identified by RepeatMasker (www.repeatmasker.org) and the signatures of each family of TE [54] using cross_match. The intact, truncated, recombination LTR elements and solo-LTRs were manually identified from the outputs of RepeatMasker and LTR_FINDER [64]. For sorghum and *B. distachyon* repeats analyses, the intact LTR retrotransposon sequences were isolated according to the results from LTR_FINDER, and then blasted against RepeatMasker TE libraries for subfamily classification.

### Isolation of CCT Family Members from Rice, *B. distachyon*, Sorghum and Maize

The *Ghd7* CCT domain protein sequence was used to search the rice, *B. distachyon*, sorghum and maize protein database using BLASTP with the following criterion: E-value ≤1e-5. The CDS of *Ghd7* homolog candidates were aligned using MUSCLE [65] and imported into GeneDoc (http://www.nrbsc.org/gfx/gene-doc/index.html) for manual adjustment. The phylogenetic tree was built using MEGA4.0 [66]. The Neighbor-Joining method was used with the following parameters: pairwise deletion; bootstrap 1000 replicates and Kimura 2-parameter model. The domain of each gene was identified with SMART (http://smart.embl-heidelberg.de/smart/set_mode.cgi?NORMAL=1) and Pfam analysis. The dotplot analysis of *Ghd7* homologous gene regions were carried out in Plant Genome Duplication Database [20] (http://chibba.agtec.uga.edu/duplication/index/dotplot).

### Insertion Dating of LTR Retrotransposon

The insertion times of LTR retrotransposons were estimated by the divergence time (*T*) between two LTRs of single intact LTR retrotransposon [67], *T* = *K*/2*r*, where *Ks* refers to the distance between the two LTRs and *r* refers to the average substation rate. The two LTRs were aligned using MUSCLE [65]. The distance between each pair of LTRs (*K*) were calculated using the *baseml* program (runmode  = 2; model  = 4) described in PAML [68]. We used substitution rate (*r*) of 1.3×10^-8^ substitutions per site per year to estimate the divergence time of LTR as repeat elements were suggested to evolve much more rapid than coding regions [32], [69].

### Analysis of the Molecular Mechanism of Gene Movement

Dot plot alignment was used to determine the borders of the repetitive elements in species without TE libraries. Target site duplications around genes and repetitive elements were identified using DOTTER [70]. Tandem repeat motifs around target genes were identified using Tandem Repeats Finder (http://tandem.bu.edu/trf/trf.submit.options.html) and RepeatMasker.

### Fluorescence In Situ Hybridization (FISH)

For *japonica* pachytene FISH, a PAC P0046D03 was used as probe (obtained from National Institute of Agrobiological Sciences in Japan; http://www.nias.affrc.go.jp/index_e.html). The chromosome 7 marker (BAC a0050F10) was obtained from Clemson University in USA (http://www.clemson.edu/). The FISH procedure applied to meiotic chromosomes was essentially the same as previously published protocols [71].

## Supporting Information

Figure S1 **Gene features of the** ***Ghd7*** **regions in diploid** ***Oryza*** **species,** ***B. distachyon*** **,** ***S. bicolor*** **and** ***Z. mays*** **.** Each gene is represented by a colored square. The yellow square with black borders indicates that the gene or gene fragment was captured by a transposable element. The numbers in purple rectangles represent the sequence length of the five complex regions in sorghum. The gene number is indicated above the black line and is summarized in Table S5. The “gap” indicates the non-overlapping regions. The abbreviation of each species is shown on the left.
(TIF)

Figure S2 **A comparison of gene densities in the** ***Ghd7*** **,** ***Adh1*** **,** ***Hd1*** **and** ***Moc1*** **regions of** ***Oryza*** **species.** *O. glumaepatula* is included for the comparative analysis of the *Ghd7* region only.
(TIF)

Figure S3 **Gene movements are mediated by repeat elements.** The genes or gene fragments are in yellow. The gene number is in the yellow polygons. The name and types of transposable elements are shown. Target site duplications are shown by flanking the terminal inverted repeats.
(TIF)

Figure S4 **RT-PCR results of** ***Ghd7*** **orthologs in eight** ***Oryza*** **species.**
(TIF)

Table S1 **BAC clones covering the** ***Ghd7*** **regions in** ***Oryza*** **species.**
(DOCX)

Table S2 **Genomic features of the** ***Ghd7*** **orthologous regions.**
(DOCX)

Table S3 **List of genes in the** ***Ghd7*** **regions of** ***O. sativa*** **L. ssp.** ***japonica*** **.**
(DOCX)

Table S4 **The gene models of** ***O. sativa*** **L. ssp.** ***japonica*** **derived from a comparative analysis.**
(DOCX)

Table S5 **List of shared genes, unshared genes or gene fragments within the** ***Ghd7*** **regions in** ***Oryza*** **species.**
(DOCX)

Table S6 **List of genes in the corresponding orthologous region of** ***B. distachyon*** **.**
(DOCX)

Table S7 **List of genes in the corresponding orthologous region of** ***S. bicolor*** **.**
(DOCX)

Table S8 **List of genes in the corresponding orthologous region of** ***Z. mays*** **.**
(DOCX)

Table S9 **Annotation of intact DNA transposable elements.**
(DOCX)

Table S10 **List of intact retrotransposons, solo-LTRs and their conservation in** ***Oryza*** **species.**
(DOCX)

Table S11 **List of intact DNA transposons and their conservation in** ***Oryza*** **species.**
(DOCX)

Table S12 **Comparison of** ***Gypsy*** **and** ***Copia*** **content in the** ***Ghd7*** **,** ***Adh1*** **and** ***Hd1*** **regions.**
(DOCX)

Table S13 **Comparison of gene densities and TE contents in the** ***Ghd7*** **,** ***Adh1*** **,** ***Hd1*** **and** ***Moc1*** **regions.**
(DOCX)

Table S14 **Accession number of CCT family genes from rice,** ***B. distachyon*** **,** ***S. bicolor*** **and** ***Z. mays*** **.**
(DOCX)

Table S15 **Chromosomal location and sequence size of** ***Ghd7*** **-related genes.**
(DOCX)
