# Supplementary figures and images for: Comparative Sequence Analysis of the Ghd7 Orthologous Regions Revealed Movement of Ghd7 in the Grass Genomes
Source: PLoS One. 2012 Nov 21;7(11):e50236. doi: 10.1371/journal.pone.0050236 (PMC3503983; doi:10.1371/journal.pone.0050236)

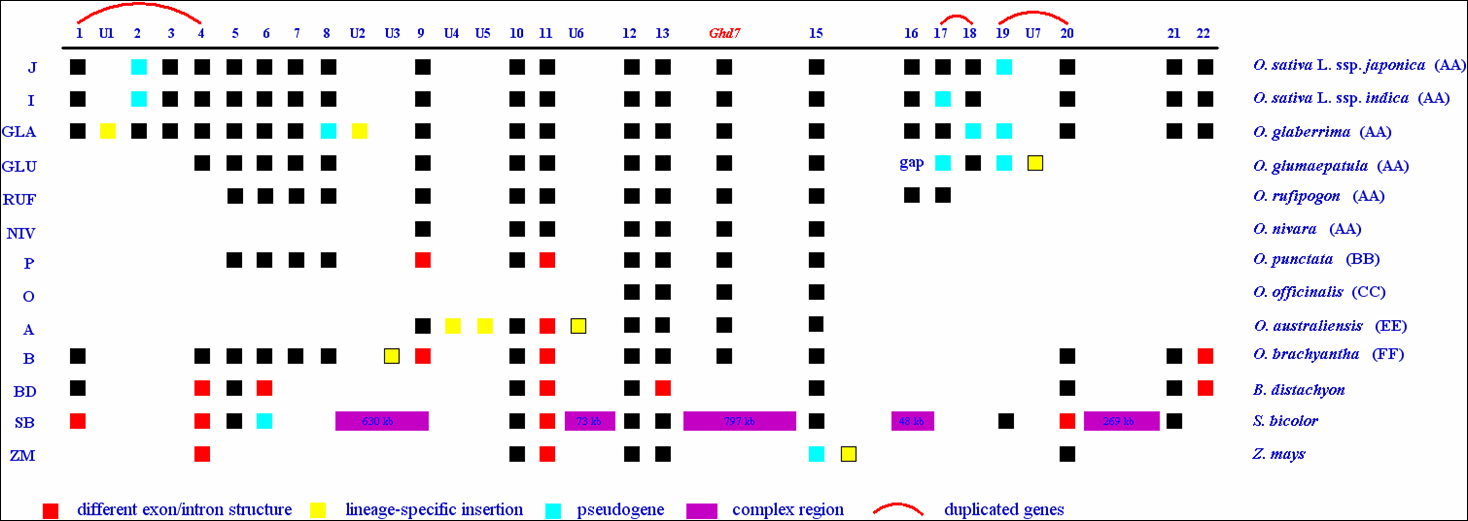

Supplement: Figure S1 — Gene features of the Ghd7 regions in diploid Oryza species, B. distachyon , S. bicolor and Z. mays . Each gene is represented by a colored square. The yellow square with black borders indicates that the gene or gene fragment was captured by a transposable element. The numbers in purple rectangles represent the sequence length of the five complex regions in sorghum. The gene number is indicated above the black line and is summarized in Table S5. The “gap” indicates the non-overlapping regions. The abbreviation of each species is shown on the left. (TIF) [file pone.0050236.s001.tif]

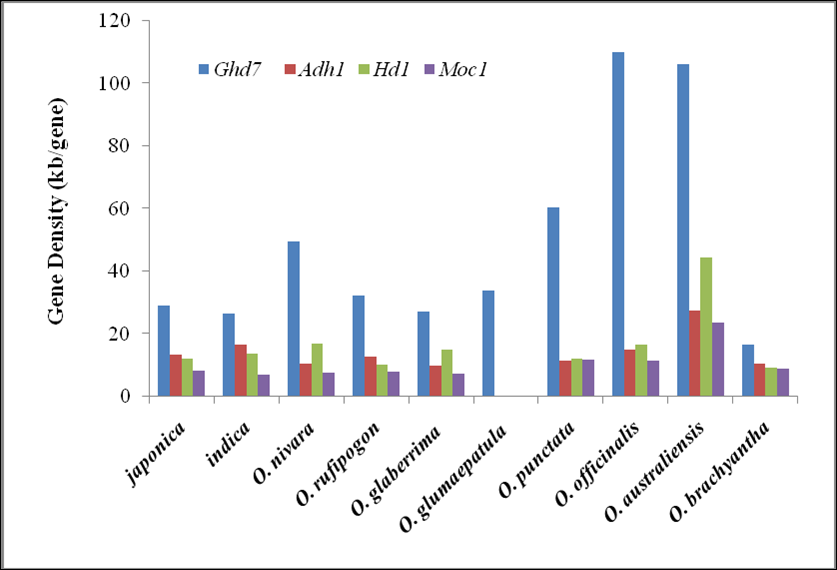

Supplement: Figure S2 — A comparison of gene densities in the Ghd7 , Adh1 , Hd1 and Moc1 regions of Oryza species. O. glumaepatula is included for the comparative analysis of the Ghd7 region only. (TIF) [file pone.0050236.s002.tif]

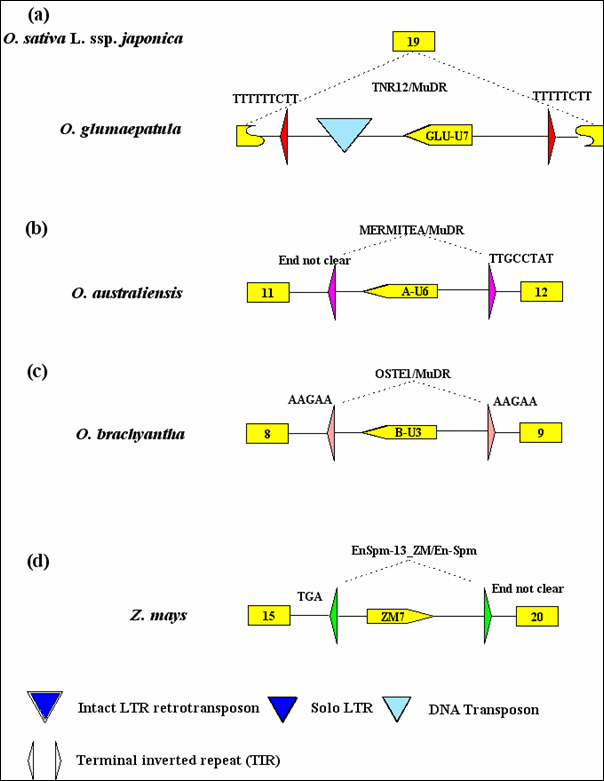

Supplement: Figure S3 — Gene movements are mediated by repeat elements. The genes or gene fragments are in yellow. The gene number is in the yellow polygons. The name and types of transposable elements are shown. Target site duplications are shown by flanking the terminal inverted repeats. (TIF) [file pone.0050236.s003.tif]

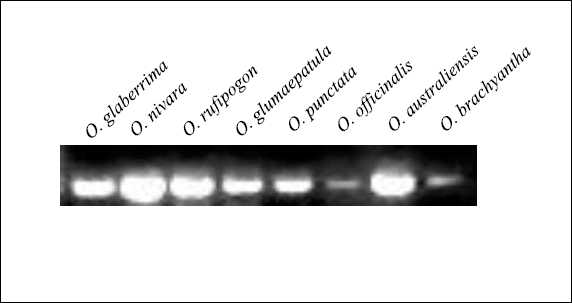

Supplement: Figure S4 — RT-PCR results of Ghd7 orthologs in eight Oryza species. (TIF) [file pone.0050236.s004.tif]
