# Supplementary material for: Comparative Sequence Analysis of the Ghd7 Orthologous Regions Revealed Movement of Ghd7 in the Grass Genomes
Source: PLoS One. 2012 Nov 21;7(11):e50236. doi: 10.1371/journal.pone.0050236 (PMC3503983; doi:10.1371/journal.pone.0050236)
Supplement: Table S1 — BAC clones covering the Ghd7 regions in Oryza species. (DOCX) [file pone.0050236.s005.docx]

**Table S1** BAC clones covering the *Ghd7* regions in *Oryza* species.

|  | Genome type | Clone Address | Insert Size (bp) | Total Length Sequenced |
| --- | --- | --- | --- | --- |
| *O. glaberrima* | AA | OG_BBa01D19^a^ | 141,474 | 514,555^b^ |
|  |  | OG_BBa67O20^a^ | 294,849 |  |
|  |  | OG_BBa89K05 | 150,094 |  |
| *O. rufipogon* | AA | OR_CBa03A17^a^ | 139,663 | 419,405 |
|  |  | OR_CBa11E20^a^ | 115,539 |  |
|  |  | OR_CBa34O09 | 168,978 |  |
|  |  | OR_CBa47B10 | 131,985 |  |
|  |  | OR_CBa24P03 | 133,611 |  |
| *O. glumaepatula* | AA | OG_EBa09A20 | 177,017 | 439,498 |
|  |  | OG_EBa68C15^a^ | 227,891 |  |
|  |  | OG_EBa63B05^a^ | 27,818 |  |
|  |  | OG_EBa106O14 | 120,434 |  |
|  |  | OG_EBa162O14 | 145,660 |  |
| *O. nivara* | AA | OR_BBa95E20^a^ | 192,302 | 346,186 |
|  |  | OR_BBa128I03 | 202,433 |  |
|  |  | OR_BBa33E21 | 187,941 |  |
| *O. punctata* | BB | OP_Ba20P16^a^ | 188,211 | 665,912 |
|  |  | OP_Ba04C12^a^ | 182,354 |  |
|  |  | OP_Ba14G24^a^ | 173,660 |  |
|  |  | OP_Ba13G18^a^ | 193,805 |  |
|  |  | OP_Ba18C07 | 133,105 |  |
|  |  | OP_Ba13B20 | 172,610 |  |
|  |  | OP_Ba68H01 | 196,294 |  |
| *O. officinalis* | CC | OO_Ba113G01^a^ | 258,843 | 440,945 |
|  |  | OO_Ba47F06 ^a^ | 273,447 |  |
|  |  | OO_Ba46E10 | 244,799 |  |
| *O. australiensis* | EE | OA_CBa34D10 | 228,105 | 744,605 |
|  |  | OA_CBa165D12 | 127,850 |  |
|  |  | OA_CBa178E24 | 144,140 |  |
|  |  | OA_CBa07H08 | 252,608 |  |
|  |  | OA_CBa167D03 | 124,530 |  |
|  |  | OA_CBa130A18 | 181,472 |  |
| *O. brachyantha* | FF | OB_Ba09F13 ^a^ | 197,437 | 284,909 ^b^ |
| Total |  |  | 5,830,959 | 3,856,015 |

^a^Sequenced by Illumina Genome Analyzer sequencing technology. The other BACs were sequenced by Roche/454GenomeSequencer FLX Instrument.

^b^Obtained from unpublished whole genome data.
