# Supplementary material for: Comparative Sequence Analysis of the Ghd7 Orthologous Regions Revealed Movement of Ghd7 in the Grass Genomes
Source: PLoS One. 2012 Nov 21;7(11):e50236. doi: 10.1371/journal.pone.0050236 (PMC3503983; doi:10.1371/journal.pone.0050236)
Supplement: Table S2 — Genomic features of the Ghd7 orthologous regions. (DOCX) [file pone.0050236.s006.docx]

**Table S2** Genomic features of the *Ghd7* orthologous regions.

|  | *O.* | *O.* | *O.* | *O.* | *O.* | *O.* | *O.* | *O.* | *O.* | *O.* | *B. distachyon* | *S. bicolor* | *Z. mays* |
| --- | --- | --- | --- | --- | --- | --- | --- | --- | --- | --- | --- | --- | --- |
|  | *sativa* L. | *sativa* L. | *nivara* | *rufipogon* | *glaberrima* | *glumaepatula* | *punctata* | *officinalis* | *australiensis* | *brachyantha* |  |  |  |
|  | ssp. | ssp. | (AA) | (AA) | (AA) | (AA) | (BB) | (CC) | (EE) | (FF) |  |  |  |
|  | *japonica* | *indica* |  |  |  |  |  |  |  |  |  |  |  |
|  | (AA) | (AA) |  |  |  |  |  |  |  |  |  |  |  |
| Genome Size (Mb) | 397 | 397 | 448 | 439 | 354 | 464 | 423 | 653 | 960 | 338 | 272 | 750 | 2400 |
| Sequence Size (bp) | 553,000 | 518,692 | 346,186 | 419,405 | 514,555 | 439,498 | 665,912 | 440,945 | 744,605 | 284,909 | 107,000 | 1,911,000 | 300,000 |
| Intact Genes | 20 | 19 | 7 | 13 | 19 | 13 | 11 | 4 | 7 | 16 | 12 | 56 | 6 |
| Pseudogenes/ fragments | 2 | 2 | No | No | 5 | 3 | No | No | 3 | 2 | No | 4 | 2 |
| Gene density (kb/gene) | 29.11 | 26.4 | 49.46 | 32.26 | 27.08 | 33.81 | 60.54 | 110.24 | 106.37 | 17.81 | 8.92 | 34.13 | 38.33 |
| Gene region (%) | 12.41 | 13.29 | 8.15 | 9.46 | 11.5 | 9.78 | 5.19 | 3.94 | 4.24 | 20.43 | 42.16 | 5.9 | 11.63 |
| TE region (%) | 48.99 | 44.89 | 66.76 | 65.93 | 43.01 | 53.28 | 70.96 | 61.28 | 60.03 | 29.5 | 6.81 | 48.3 | 78.04 |
| GC content (%) | 42.53 | 39.09 | 43.48 | 42.74 | 40.81 | 42.54 | 43.41 | 42.14 | 44.29 | 40.66 | 43.19 | 41.3 | 46.5 |
