# Supplementary material for: Comparative Sequence Analysis of the Ghd7 Orthologous Regions Revealed Movement of Ghd7 in the Grass Genomes
Source: PLoS One. 2012 Nov 21;7(11):e50236. doi: 10.1371/journal.pone.0050236 (PMC3503983; doi:10.1371/journal.pone.0050236)
Supplement: Table S3 — List of genes in the Ghd7 regions of O. sativa L. ssp. japonica . (DOCX) [file pone.0050236.s007.docx]

**Table S3** List of genes in the *Ghd7* regions of *O. sativa* L. ssp. *japonica*.

| Gene | Classification | Putative gene product | Identification method | | | TIGR Rice model V8 |
| --- | --- | --- | --- | --- | --- | --- |
|  |  |  | Transcript evidence | Accession | Known functional domain |  |
|  |  |  | full-length cDNA |  | domain name |  |
| J-1 | Expressed | Metal transporter Nramp6 | AK070788;  CT832446 | PF01566 | Nramp6 | LOC_Os07g15370 |
|  |  |  |  |  |  |  |
| J-2 | Psuedo | NONE | AK109782 | NONE | NONE | LOC_Os07g15430 |
| J-3 | Expressed | Alanyl-tRNA synthetase family protein | AK064037;  NM_001065849 | PF01411/  PF04424 | tRNA synthetases class II (A)/  DUF544 | LOC_Os07g15440 |
| J-4 | Expressed | Metal transporter Nramp6 | AK121534;  DQ431468 | PF01566 | Nramp6 | LOC_Os07g15460 |
|  |  |  |  |  |  |  |
| J-5 | Expressed | C2-BTB1 Bric-a-Brac Tramtrack Broad Complex BTB domain with C2 subfamily conserved sequence | AK068718;  AK106061;  AK069407;  CT832693 | PF00651 | BTB/POZ | LOC_Os07g15490 |
| J-6 | Expressed | Expressed protein | NM_001188195 | NONE | NONE | LOC_Os07g15500 |
| J-7 | Expressed | Expressed protein | AK106951 | NONE | NONE | LOC_Os07g15530 |
| J-8 | Expressed | Ethylene receptor | AY434734;  AK109593;  AF497626 | PF01590/  PF00072 | GAF domain/Response regulator receiver domain | LOC_Os07g15540 |
| J-9 | Expressed | Pentatricopeptide | NM_001065855.2 | PF01535 | PPR repeat | LOC_Os07g15570 |
| J-10 | Expressed | H-BTB6 Bric-a-Brac Tramtrack Broad Complex BTB domain with H family conserved sequence | AK067947 | NONE | NONE | LOC_Os07g15600 |
| J-11 | Expressed | CRR4 | NM_001065857 | PF01535 | PPR | LOC_Os07g15640 |
| J-12 | Expressed | Peroxiredoxin | AK066852;  CT829695 | PF00578 | AhpC/TSA family | LOC_Os07g15670 |
|  |  |  |  |  |  |  |
| J-13 | Expressed | Phospholipase D | AK070203 | PF00168/  PF00614 | C2 domain/Phospholipase D Active site motif | LOC_Os07g15680 |
| J-14 | Expressed | CCT motif family protein | EU286800 | PF06203 | CCT motif | LOC_Os07g15770 |
| J-15 | Expressed | Mitochondrial prohibitin complex protein 2 | AK071615;  CT832283;  AK104133 | PF01145 | PHB | LOC_Os07g15880 |
| J-16 | Hypothetical | NONE | NONE | NONE | NONE | LOC_Os07g15920 |
| J-17 | Expressed | Legume lectins beta domain containing protein | NM_001188197.1 | PF00139/  PF00069 | Legume lectin domain/Protein kinase domain | LOC_Os07g15930 |
| J-18 | Expressed |  | AK120208 |  |  | LOC_Os07g15940 |
| J-19 | Psuedo | Erythronate-4phosphate dehydrogenase domain containing protein | NONE | PF00389/  PF02826 | D-isomer specific 2-hydroxyacid dehydrogenase catalytic domain/ D-isomer specific 2-hydroxyacid dehydrogenaseNAD binding domain | LOC_Os07g15970 |
| J-20 | Expressed |  | AK065209;  AK104725;  AK104560 |  |  | LOC_Os07g16040 |
| J-21 | Expressed | Acetyltransferase | AK066403;  AK104697 | PF00583 | Acetyltransferase (GNAT) family | LOC_Os07g16130 |
| J-22 | Expressed | FAD binding protein | CT828613;  AK071462 | NONE | NONE | LOC_Os07g16140 |
