# Supplementary material for: Comparative Sequence Analysis of the Ghd7 Orthologous Regions Revealed Movement of Ghd7 in the Grass Genomes
Source: PLoS One. 2012 Nov 21;7(11):e50236. doi: 10.1371/journal.pone.0050236 (PMC3503983; doi:10.1371/journal.pone.0050236)
Supplement: Table S4 — The gene models of O. sativa L. ssp. japonica derived from a comparative analysis. (DOCX) [file pone.0050236.s008.docx]

**Table S4** The gene models of *O. sativa* L. ssp. *japonica* derived from a comparative analysis.

| No. | TIGR model (V8) | Classification | Putative function | Omission reason |
| --- | --- | --- | --- | --- |
| 1 | LOC_Os07g15360 | Hypothetical | Retrotransposon protein | Retrotransposon protein putative Ty1-copia subclass |
| 2 | LOC_Os07g15380 | Hypothetical | Hypothetical protein | No transcript and conserved domain evidence support, No homology out of AA genome |
| 3 | LOC_Os07g15390 | Hypothetical | Retrotransposon protein, putative, unclassified | Retrotransposon protein |
| 4 | LOC_Os07g15400 | Hypothetical | Hyprothetical protein | No transcript and conserved domain evidence support, No homology in other genome |
| 5 | LOC_Os07g15410 | Hypothetical | Retrotransposon protein, putative, unclassified | Retrotransposon protein |
| 6 | LOC_Os07g15420 | Hypothetical | Transposon protein | Transposon protein, AcDs subclass |
| 7 | LOC_Os07g15450 | Hypothetical | Retrotransposon protein, putative, unclassified | (RETRO2/Gypsy ) Retrotransposon protein |
| 8 | LOC_Os07g15470 | Hypothetical | Retrotransposon protein,putative, unclassified | (COPIO/Copia) Retrotransposon protein |
| 9 | LOC_Os07g15480 | Hypothetical | Retrotransposon protein,putative, unclassified | Retrotransposon protein |
| 10 | LOC_Os07g15510 | Hypothetical | Retrotransposon protein,putative, unclassified | (SZ-10/Gypsy) Retrotransposon protein |
| 11 | LOC_Os07g15520 | Hypothetical | Retrotransposon protein, putative, Ty3-gypsy subclass | (SZ-10/Gypsy) Retrotransposon protein |
| 12 | LOC_Os07g15550 | Hypothetical | Retrotransposon protein, putative, unclassified | (OSCopia2/Copia) Retrotransposon protein |
| 13 | LOC_Os07g15560 | Hypothetical | Retrotransposon protein, putative, unclassified | (OSCopia2/Copia) Retrotransposon protein |
| 14 | LOC_Os07g15580 | Hypothetical | Hypothetical protein | No transcript and conserved domain evidence support, No homology protein |
| 15 | LOC_Os07g15590 | Hypothetical | Hypothetical protein | No transcript and conserved domain evidence support, No homology protein |
| 16 | LOC_Os07g15610 | Hypothetical | Hypothetical protein | (GypsO/Gypsy) Retrotransposon protein |
| 17 | LOC_Os07g15620 | Hypothetical | Retrotransposon protein, putative, unclassified | (RETRO2/Gypsy ) Retrotransposon protein |
| 18 | LOC_Os07g15630 | Hypothetical | Retrotransposon protein, putative, unclassified | (RETRO2/Gypsy ) Retrotransposon protein |
| 19 | LOC_Os07g15650 | Hypothetical | Hypothetical protein | No transcript and conserved domain evidence support, No homology protein |
| 20 | LOC_Os07g15660 | Hypothetical | Retrotransposon protein, putative, LINE subclass | OSLINE1-4\|LINE/L1 Retrotransposon protein |
| 21 | LOC_Os07g15690 | Hypothetical | Transposon protein, putative, CACTA EnSpm sub class | ENSPM2_OS\|DNA/En-Spm, Ttransposon protein |
| 22 | LOC_Os07g15700 | Hypothetical | Transposon protein, putative, unclassified | Transposon protein |
| 23 | LOC_Os07g15710 | Hypothetical | Transposon protein, putative, unclassified | Transposon protein |
| 24 | LOC_Os07g15720 | Hypothetical | Hypothetical protein | (ATLANTYS/Gypsy) Retrotransposon protein |
| 25 | LOC_Os07g15730 | Hypothetical | Retrotransposon protein, putative, Ty3-gypsy subclass | (ATLANTYS/Gypsy) Retrotransposon protein |
| 26 | LOC_Os07g15740 | Hypothetical | Retrotransposon protein, putative, Ty3-gypsy subclass | (ATLANTYS/Gypsy) Retrotransposon protein |
| 27 | LOC_Os07g15750 | Hypothetical | Retrotransposon protein, putative, Ty3-gypsy subclass | (ATLANTYS/Gypsy) Retrotransposon protein |
| 28 | LOC_Os07g15760 | Hypothetical | Retrotransposon protein, putative, Ty3-gypsy subclass | (ATLANTYS/Gypsy) Retrotransposon protein |
| 29 | LOC_Os07g15780 | Hypothetical | Retrotransposon protein, putative, Ty1-copia subclass | (COPIA2/Copia) Retrotransposon protein |
| 30 | LOC_Os07g15790 | Hypothetical | Transposon protein, putative, CACTA EnSpm subclass | SPMLIKE\|DNA/En-Spm, Transposon protein |
| 31 | LOC_Os07g15800 | Hypothetical | Transposon protein, putative, CACTA EnSpm subclass | SPMLIKE\|DNA/En-Spm , Transposon protein |
| 32 | LOC_Os07g15809 | Hypothetical | Hypothetical protein | No transcript and conserved domain evidence support, No homology in other genome |
| 33 | LOC_Os07g15820 | Hypothetical | Hypothetical protein | No transcript and conserved domain evidence support, No homology in other genome |
| 34 | LOC_Os07g15830 | Hypothetical | Retrotransposon protein, putative, unclassified | (SZ-66/Gypsy) Retrotransposon protein |
| 35 | LOC_Os07g15840 | Hypothetical | Retrotransposon, putative, centromere-specific | (SZ-38/Gypsy) Retrotransposon protein |
| 36 | LOC_Os07g15850 | Hypothetical | Retrotransposon protein, putative, Ty1-copia subclass | (RIRE5/Copia) Retrotransposon protein |
| 37 | LOC_Os07g15860 | Hypothetical | Hypothetical protein | No transcript and conserved domain evidence support, No homology in other genome |
| 38 | LOC_Os07g15870 | Hypothetical | Hypothetical protein | No transcript and conserved domain evidence support, No homology protein |
| 39 | LOC_Os07g15890 | Hypothetical | Retrotransposon protein, putative, Ty1-copia subclass | (RIRE1/Copia) Retrotransposon protein |
| 40 | LOC_Os07g15900 | Hypothetical | Hypothetical protein | (SZ-35/Gypsy) Retrotransposon protein |
| 41 | LOC_Os07g15910 | Hypothetical | Conserved hypothetical protein | ENSPM7_OS\|DNA/En-Spm, Transposon protein |
| 42 | LOC_Os07g15950 | Hypothetical | Hypothetical protein | MERMITE18D\|DNA/MuDR, Transposon protein |
| 43 | LOC_Os07g15959 | Hypothetical | Expressed protein | (SZ-36/Gypsy) Retrotransposon protein |
| 44 | LOC_Os07g15980 | Hypothetical | Hypothetical protein | No transcript and conserved domain evidence support, No homology protein |
| 45 | LOC_Os07g15990 | Hypothetical | Retrotransposon protein, putative, Ty3-gypsy subclass | (RETROSAT5/Gypsy) Retrotransposon protein |
| 46 | LOC_Os07g16000 | Hypothetical | Retrotransposon protein, putative, Ty3-gypsy subclass | (RETROSAT5/Gypsy) Retrotransposon protein |
| 47 | LOC_Os07g16010 | Hypothetical | Retrotransposon protein, putative, Ty3-gypsy subclass | (RETROSAT5/Gypsy) Retrotransposon protein |
| 48 | LOC_Os07g16019 | Hypothetical | Hypothetical protein | No transcript and conserved domain evidence support, No homology in other genome |
| 49 | LOC_Os07g16030 | Hypothetical | Expressed protein | No transcript and conserved domain evidence support, No homology in other genome |
| 50 | LOC_Os07g16054 | Hypothetical | Hypothetical protein | (RETROSOR2/Gypsy) Retrotransposon protein |
| 51 | LOC_Os07g16070 | Hypothetical | Retrotransposon protein, putative, unclassified | (SZ-7/Gypsy) Retrotransposon protein |
| 52 | LOC_Os07g16080 | Hypothetical | Retrotransposon protein, putative, unclassified | (SZ-7/Gypsy) Retrotransposon protein |
| 53 | LOC_Os07g16090 | Hypothetical | Retrotransposon protein, putative, unclassified | (RIREX/Gypsy) Retrotransposon protein |
| 54 | LOC_Os07g16100 | Hypothetical | Rretrotransposon protein, putative, unclassified | (RIREX/Gypsy) Retrotransposon protein |
| 55 | LOC_Os07g16110 | Hypothetical | Retrotransposon protein, putative, unclassified | (RIREX/Gypsy) Retrotransposon protein |
| 56 | LOC_Os07g16120 | Hypothetical | Retrotransposon protein, putative, unclassified | (SZ-7/Gypsy) Retrotransposon protein |
