# Supplementary material for: Comparative Sequence Analysis of the Ghd7 Orthologous Regions Revealed Movement of Ghd7 in the Grass Genomes
Source: PLoS One. 2012 Nov 21;7(11):e50236. doi: 10.1371/journal.pone.0050236 (PMC3503983; doi:10.1371/journal.pone.0050236)
Supplement: Table S5 — List of shared genes, unshared genes or gene fragments within the Ghd7 regions in Oryza species. (DOCX) [file pone.0050236.s009.docx]

**Table S5** List of shared genes, unshared genes or gene fragments within the *Ghd7* regions in *Oryza* species.

| No. | TIGR Rice model V8 | *O. sativa* L. ssp. *japonica* (AA) | *O. sativa* L. ssp. *indica*(AA) | *O. glaberrima* (AA) | *O. rufipogon* (AA) | *O. nivara* (AA) | *O. glumaepatula* (AA) | *O. punctata* (BB) | *O. officinalis* (CC) | *O. australiensis* (EE) | *O. brachyantha* (FF) | Information |
| --- | --- | --- | --- | --- | --- | --- | --- | --- | --- | --- | --- | --- |
|  |  | J | I | GLA | RUF | NIV | GLU | P | O | A | B |  |
| 1 | LOC_Os07g15370 | J-1 | I-1 | GLA-1 | NC | NC | NC | NC | NC | NC | B-1 |  |
| U1 |  | * | * | GLA-U1 | NC | NC | NC | NC | NC | NC | * | Homolog to LOC_Os04g30860 |
| 2 | LOC_Os07g15430 | J-2 | I-2 | GLA-2 | NC | NC | NC | NC | NC | NC | * |  |
| 3 | LOC_Os07g15440 | J-3 | I-3 | GLA-3 | NC | NC | NC | NC | NC | NC | * |  |
| 4 | LOC_Os07g15460 | J-4 | I-4 | GLA-4 | NC | NC | GLU-4 | NC | NC | NC | B-4 |  |
| 5 | LOC_Os07g15490 | J-5 | I-5 | GLA-5 | RUF-5 | NC | GLU-5 | P-5 | NC | NC | B-5 |  |
| 6 | LOC_Os07g15500 | J-6 | I-6 | GLA-6 | RUF-6 | NC | GLU-6 | P-6 | NC | NC | B-6 |  |
| 7 | LOC_Os07g15530 | J-7 | I-7 | GLA-7 | RUF-7 | NC | GLU-7 | P-7 | NC | NC | B-7 |  |
| 8 | LOC_Os07g15540 | J-8 | I-8 | GLA-8 | RUF-8 | NC | GLU-8 | P-8 | NC | NC | B-8 |  |
| U2 |  | * | * | GLA-U2 | * | * | * | * | * | * | * | Homolog to LOC_Os05g34200 |
| U3 |  | * | * | * | * | * | * | * | * | * | B-U3 | Gene fragment; homolog to LOC_Os10g34340; embedded by OSTE1/MuDR |
| 9 | LOC_Os07g15570 | J-9 | I-9 | GLA-9 | RUF-9 | NIV-9 | GLU-9 | P-9 | NC | A-9 | B-9 |  |
| U4 |  | * | * | * | * | * | * | * | * | A-U4 | * | Homolog to LOC_Os10g24954.1 (ulp1 protease family) |
| U5 |  | * | * | * | * | * | * | * | * | A-U5 | * | Utp11 domain; homolog to LOC_Os01g59500 (U3 small nucleolar RNA-associated protein 11) |
| 10 | LOC_Os07g15600 | J-10 | I-10 | GLA-10 | RUF-10 | NIV-10 | GLU-10 | P-10 | NC | A-10 | B-10 |  |
| 11 | LOC_Os07g15640 | J-11 | I-11 | GLA-11 | RUF-11 | NIV-11 | GLU-11 | P-11 | NC | A-11 | B-11 |  |
| U6 |  | * | * | * | * | * | * | * | * | A-U6 | * | Homolog to LOC_Os02g07930 (B-box zinc finger family protein); mediated by MERMITEA/MuDR |
| 12 | LOC_Os07g15670 | J-12 | I-12 | GLA-12 | RUF-12 | NIV-12 | GLU-12 | P-12 | O-12 | A-12 | B-12 |  |
| 13 | LOC_Os07g15680 | J-13 | I-13 | GLA-13 | RUF-13 | NIV-13 | GLU-13 | P-13 | O-13 | A-13 | B-13 |  |
| 14 | LOC_Os07g15770 | J-14 | I-14 | GLA-14 | RUF-14 | NIV-14 | GLU-14 | P-14 | O-14 | A-14 | B-14 |  |
| 15 | LOC_Os07g15880 | J-15 | I-15 | GLA-15 | RUF-15 | NIV-15 | GLU-15 | P-15 | O-15 | A-15 | B-15 |  |
| 16 | LOC_Os07g15920 | J-16 | I-16 | GLA-16 | RUF-16 | NC | NC | * | * | * | * |  |
| 17 | LOC_Os07g15930 | J-17 | I-17 | GLA-17 | RUF-17 | NC | GLU-17 | NC | NC | NC | * |  |
| 18 | LOC_Os07g15940 | J-18 | I-18 | GLA-18 | NC | NC | GLU-18 | NC | NC | NC | * |  |
| 19 | LOC_Os07g15970 | J-19 | * | GLA-19 | NC | NC | GLU-19 | NC | NC | NC | * |  |
| U7 |  | * | * | * | * | * | GLU-U7 | * | * | * | * | Homology to LOC_Os05g04390, GRF domain; mediated by NR12\|DNA/MuDR |
| 20 | LOC_Os07g16040 | J-20 | I-20 | GLA-20 | NC | NC | NC | NC | NC | NC | B-20 |  |
| U8 |  | * | * | * | * | * | * | * | * | * | B-U8 | Gene fragment; homology to LOC_Os10g34540 |
| 21 | LOC_Os07g16130 | J-21 | I-21 | GLA-21 | NC | NC | NC | NC | NC | NC | B-21 |  |
| 22 | LOC_Os07g16140 | J-22 | I-22 | GLA-22 | NC | NC | NC | NC | NC | NC | B-22 |  |
| * | Inexistence |  |  |  |  |  |  |  |  |  |  |  |
| NC | No coverage |  |  |  |  |  |  |  |  |  |  |  |
