# Supplementary material for: Comparative Sequence Analysis of the Ghd7 Orthologous Regions Revealed Movement of Ghd7 in the Grass Genomes
Source: PLoS One. 2012 Nov 21;7(11):e50236. doi: 10.1371/journal.pone.0050236 (PMC3503983; doi:10.1371/journal.pone.0050236)
Supplement: Table S6 — List of genes in the corresponding orthologous region of B. distachyon . (DOCX) [file pone.0050236.s010.docx]

**Table S6** List of genes in the corresponding orthologous region of *B. distachyon*.

| Gene | Classification | Putative gene product | Identification method | | | Ortholog in rice |
| --- | --- | --- | --- | --- | --- | --- |
|  |  |  | Transcript evidence | Known functional domain | |  |
|  |  |  | Fl-cDNA | Accession | Domain name |  |
| BD-1 | Expressed | Metal transporter Nramp6 | Bradi1g53160.1 | PF01566 | Nramp | LOC_Os07g15370 |
| BD-4 | Expressed | Metal transporter Nramp6 | Bradi1g53150.1 | PF01566 | Nramp | LOC_Os07g15460 |
| BD-5 | Expressed | C2-BTB1-Bric-a-Brac Tramtrack Broad Complex BTB domain with C2 subfamily | Bradi1g53140.1 | PF00651 | BTB | LOC_Os07g15490 |
| BD-6 | Expressed | Expressed protein | Bradi1g53130.1 | NONE | NONE | LOC_Os07g15500 |
| BD-10 | Expressed | H-BTB6-Bric-a-Brac, Tramtrack, Broad Complex BTB domain with H family | Bradi1g53120.1 | NONE | NONE | LOC_Os07g15600 |
| BD-11 | Expressed | Pentatricopeptide repeat (PPR) proteins | Bradi1g53110.1 | PF01535 | PPR | LOC_Os07g15640 |
| BD-12 | Expressed | Peroxiredoxin | Bradi1g53090.1 | PF00578 | AhpC-TSA | LOC_Os07g15670 |
| BD-13 | Expressed | Phospholipase D | Bradi1g53080.1 | PF00168/  PF00614 | C2/  PLDc | LOC_Os07g15680 |
| BD-15 | Expressed | Mitochondrial prohibitin complex protein 2 | Bradi1g53070.1 | PF01145 | PHB | LOC_Os07g15880 |
| BD-20 | Expressed | Erythronate-4-phosphate dehydrogenase domain containing protein | Bradi1g53060.1 | PF02826 | 2-Hacid_dh_C | LOC_Os07g16040 |
| BD-21 | Expressed | Acetyltransferase, GNAT family | Bradi1g53050.1 | PF00583 | Acetyltransf_1 | LOC_Os07g16130 |
| BD-22 | Expressed | FAD binding protein | Bradi1g53040.1 | NONE | NONE | LOC_Os07g16140 |
