# Supplementary material for: Comparative Sequence Analysis of the Ghd7 Orthologous Regions Revealed Movement of Ghd7 in the Grass Genomes
Source: PLoS One. 2012 Nov 21;7(11):e50236. doi: 10.1371/journal.pone.0050236 (PMC3503983; doi:10.1371/journal.pone.0050236)
Supplement: Table S7 — List of genes in the corresponding orthologous region of S. bicolor . (DOCX) [file pone.0050236.s011.docx]

**Table S7** List of genes in the corresponding orthologous region of *S. bicolor*.

| Gene | Classification | Putative gene product | Identification method | | | Homolog in rice |
| --- | --- | --- | --- | --- | --- | --- |
|  |  |  | Transcript evidence | Known functional domain | |  |
|  |  |  | Fl-cDNA /EST | accession | domain name |  |
| SB-1 | Expressed | Metal transporter Nramp6 | Sb02g007870.1 | PF01566 | Nramp | LOC_Os07g15370 |
| SB-4 | Expressed | Metal transporter Nramp6 | Sb02g007890.1 | PF01566 | Nramp | LOC_Os07g15460 |
| SB-5 | Expressed | C2-BTB1-Bric-a-Brac Tramtrack Broad Complex BTB domain with C2 subfamily | Sb02g007900.1 | PF00651 | BTB | LOC_Os07g15490 |
| SB-6 | Psuedo/Hypothetical | Expressed protein | NONE | NONE | NONE | LOC_Os07g15500 |
| SB9 | Expressed | Minor ampullate silk protein MiSp1 | Sb02g007910.1 | NONE | NONE | LOC_Os05g28450 |
| SB23 | Expressed | Copper methylamine oxidase precursor | Sb02g007930.1 | PF02727/  PF02728/  PF01179 | Cu_amine_oxidN2/  Cu_amine_oxidN3/  Cu_amine_oxid | LOC_Os06g23114 |
| SB43 | Expressed | Serine/threonine-protein phosphatase 2A activator 2 | Sb02g007940.1 | NONE | NONE | LOC_Os06g11640 |
| SB44 | Psuedo/Hypothetical | Serine/threonine-protein phosphatase 2A activator 2 | NONE | NONE | NONE | LOC_Os06g11640 |
| SB54 | Expressed | OsFBX210 - F-box domain containing protein,A Receptor for Ubiquitination Targets | Sb02g007956.1 | PF00646 | F-box | LOC_Os07g02930 |
| SB56 | Psuedo/Hypothetical | OsFBX210 - F-box domain containing protein,A Receptor for Ubiquitination Targets | NONE | PF00646 | F-box | LOC_Os07g02930 |
| SB60 | Expressed | Expressed protein no apical meristem protein | Sb02g007960.1 | PF02365 | NAM | LOC_Os09g12380 |
| SB65 | Expressed | OsFBX209 - F-box domain containing protein | Sb02g007965.1 | PF00646 | F-box | LOC_Os07g02890 |
| SB66 | Expressed | Autophagy-related protein 3 | Sb02g007970.1 | PF03986/  PF03987 | Autophagy_N/  Autophagy_act_C | LOC_Os01g10290 |
| SB71 | Expressed | Expressed protein | Sb02g007990.1 | NONE | NONE | LOC_Os07g02710 |
| SB74 | Expressed | Conserved hypothetical protein | Sb02g008000.1 | PF00646/  PF07723/  PF08387 | F-box/  LRR_2/  FBD | LOC_Os07g18560 |
| SB78 | Expressed | OsFBLD4 - F-box, LRR and FBD domain containing protein | Sb02g008020.1 | PF08387 | FBD | LOC_Os07g13900 |
| SB80 | Expressed | OsFBLD4 - F-box, LRR and FBD domain containing protein | Sb02g008030.1 | PF00646/  PF08387 | F-Box/  FBD | LOC_Os07g18510 |
| SB81 | Expressed | Expressed protein | Sb02g008040.1 | PF08224 | DUF1719 | LOC_Os07g02970 |
| SB86 | Expressed | Expressed protein | Sb02g008053.1 | PF08224 | DUF1719 | LOC_Os07g02970 |
| SB92 | Expressed | ulp1 protease family, C-terminal catalytic domain containing protein | Sb02g008056.1 | PF02902 | Peptidase_C48 | LOC_Os01g16730 |
| SB96 | Expressed | Expressed protein | Sb02g008060.1 | PF08224 | DUF1719 | LOC_Os07g02970 |
| SB98 | Expressed | Expressed protein | Sb02g008065.1 | NONE | NONE | LOC_Os02g46920 |
| SB103 | Expressed | Expressed protein | Sb02g008070.1 | PF08224 | DUF1719 | LOC_Os07g02970 |
| SB119 | Expressed | Expressed protein | Sb02g008080.1 | PF08224 | DUF1719 | LOC_Os07g02970 |
| SB120 | Expressed | Expressed protein | Sb02g008082.1 | SM00343 | ZnF_C2HC domain | LOC_Os11g44890 |
| SB123 | Expressed | Hypothetical protein | Sb02g008086.1 | NONE | NONE | LOC_Os04g01560 |
| SB125 | Expressed | Hypothetical protein | Sb02g008090.1 | NONE | NONE | LOC_Os04g01560 |
| SB126 | Expressed | Hypothetical protein | Sb02g008095.1 | PF08224 | DUF1719 | LOC_Os04g01560 |
| SB-10 | Expressed | H-BTB6 - Bric-a-Brac, Tramtrack, Broad Complex BTB domain with H family | Sb02g008100.1 | NONE | NONE | LOC_Os07g15600 |
| SB-11 | Expressed | Pentatricopeptide repeat (PPR) proteins | Sb02g008110.1 | PF01535 | PPR | LOC_Os07g15640 |
| SB130-1 | Expressed | OsFBX230 - F-box domain containing protein | Sb02g008113.1 | PF00646/  PF08268 | F-Box/  FBA_3 | LOC_Os07g13890 |
| SB130-2 | Expressed | Legume lectins beta domain containing protein | Sb02g008116.1 | NONE | NONE | LOC_Os02g19550 |
| SB-12 | Expressed | Peroxiredoxin | Sb02g008120.1\.2 | PF00578 | AhpC-TSA | LOC_Os07g15670 |
| SB-13 | Expressed | Phospholipase D | Sb02g008130.1 | PF00168/  PF00614 | C2/  PLDc | LOC_Os07g15680 |
| SB137 | Expressed | Expressed protein | Sb02g008135.1 | PF05633 | DUF793 | LOC_Os04g59140 |
| SB198 | Expressed | Expressed protein | Sb02g008350.1 | PF08224 | DUF1719 | LOC_Os07g02970 |
| SB203 | Expressed | OsFBX234 - F-box domain containing protein, expressed | Sb02g008355.1 | NONE | NONE | LOC_Os07g23900 |
| SB205 | Expressed | OsFBX15 - F-box domain containing protein | Sb02g008357.1 | NONE | NONE | LOC_Os01g37670 |
| SB210 | Expressed | OsFBX426 - F-box domain containing protein | Sb02g008360.1 | NONE | NONE | LOC_Os11g37340 |
| SB211 | Hypothetical | OsFBX426 - F-box domain containing protein | NONE | NONE | NONE | LOC_Os11g37340 |
| SB215 | Expressed | OsFBX209 - F-box domain containing protein | Sb02g008380.1 | PF00646 | F-box | LOC_Os07g02890 |
| SB216 | Expressed | Expressed protein | Sb02g008390.1 | PF08224 | DUF1719 | LOC_Os07g02970 |
| SB219 | Expressed | Expressed protein | Sb02g008400.1 | PF08224 | DUF1719 | LOC_Os07g02970 |
| SB220 | Expressed | OsFBLD4 - F-box, LRR and FBD domain containing protein | Sb02g008410.1 | PF08387 | FBD | LOC_Os07g13900 |
| SB223 | Expressed | Histone-lysine N-methyltransferase ATX5 | Sb02g008411.1 | NONE | NONE | LOC_Os01g11952 |
| SB229 | Expressed | Hypothetical protein | Sb02g008415.1 | PF08224 | DUF1719 | LOC_Os07g02980 |
| SB235 | Expressed | Jacalin-like lectin domain containing protein | Sb02g008430.1 | PF01419 | Jacalin | LOC_Os12g09700 |
| SB236 | Expressed/gap | Mannose-6-phosphate isomerase | Sb02g008440.1 | NONE | NONE | LOC_Os11g38810 |
| SB249 | Expressed | ADP-ribosylation factor | Sb02g008541.1 | SM00010 | small_GTPase domain | LOC_Os07g12170 |
| SB-15 | Expressed | Mitochondrial prohibitin complex protein 2 | Sb02g008640.1 | PF01145 | PHB | LOC_Os07g15880 |
| SB265 | Expressed | Zinc finger, C3HC4 type domain containing protein | Sb02g008650.1 | SM00249/  PF02182/  PF00097 | PHD/  SRA/  RING | LOC_Os05g01230 |
| SB-19 | Expressed | Erythronate-4-phosphate dehydrogenase domain containing protein | Sb02g008660.1 | PF02826 | 2-Hacid_dh_C | LOC_Os07g15970 |
| SB-20 | Expressed | Erythronate-4-phosphate dehydrogenase domain containing protein | Sb02g008670.1 | PF02826 | 2-Hacid_dh_C | LOC_Os07g16040 |
| SB270 | Expressed | OsFBX279 - F-box domain containing protein | Sb02g008675.1 | PF00646 | F-box | LOC_Os08g16630 |
| SB276 | Psuedo/Hypothetical | OsFBX279 - F-box domain containing protein | NONE | PF00646 | F-box | LOC_Os08g16630 |
| SB279 | Hypothetical | OsFBX279 - F-box domain containing protein | NONE | PF00646 | F-box | LOC_Os08g16630 |
| SB283 | Expressed | OsFBX279 - F-box domain containing protein | Sb02g008680.1 | PF00646 | F-box | LOC_Os08g16630 |
| SB284 | Expressed | OsFBX279 - F-box domain containing protein | Sb02g008690.1 | PF00646 | F-box | LOC_Os08g16630 |
| SB307 | Expressed | Hypothetical protein | Sb02g008695.1 | NONE | NONE | LOC_Os10g11240 |
| SB-21 | Expressed | Acetyltransferase, GNAT family | Sb02g008700.1 | PF00583 | Acetyltransf_1 | LOC_Os07g16130 |

Genes are colored. The light blue means the orthologous genes in rice. The same color means the genes are from the same gene family.
