# Supplementary material for: Comparative Sequence Analysis of the Ghd7 Orthologous Regions Revealed Movement of Ghd7 in the Grass Genomes
Source: PLoS One. 2012 Nov 21;7(11):e50236. doi: 10.1371/journal.pone.0050236 (PMC3503983; doi:10.1371/journal.pone.0050236)
Supplement: Table S8 — List of genes in the corresponding orthologous region of Z. mays . (DOCX) [file pone.0050236.s012.docx]

**Table S8** List of genes in the corresponding orthologous region of *Z. mays*.

| Gene | Classification | Putative gene product | Identification method | | | Homolog in rice | Information |
| --- | --- | --- | --- | --- | --- | --- | --- |
|  |  |  | Transcript evidence | Known functional domain | |  |  |
|  |  |  | Fl-cDNA | Accession domain | Domain name |  |  |
| ZM-4 | Expressed | Meta transporter Nramp6 | GRMZM2G366919_T01 | PF01566 | Nramp | LOC_Os07g15460 |  |
| ZM-10 | Expressed | H-BTB6-Bric-a-Brac, Tramtrack, Broad Complex BTB domain with H family | GRMZM2G067756_T01 | NONE | NONE | LOC_Os07g15600 |  |
| ZM-11 | Expressed | Pentatricopeptide repeat (PPR) proteins | GRMZM2G367008_T01 | PF01535 | PPR | LOC_Os07g15640 |  |
| ZM-12 | Expressed | Peroxiredoxin | GRMZM2G145895_T01;  GRMZM2G145895_T02;  GRMZM2G145895_T03 | PF00578 | AhpC-TSA | LOC_Os07g15670 |  |
| ZM-13 | Expressed | Phospholipase D | GRMZM2G145944_T01;  GRMZM2G145944_T02 | PF00168/  PF00614 | C2/  PLDc | LOC_Os07g15680 |  |
| ZM6 | Fragment | Succinate dehydrogenase iron-sulfur subunit 2, mitochondrial precursor | NONE | NONE | NONE | LOC_Os08g02640 |  |
| ZM7 | Fragment | CK1_CaseinKinase_1.8 - CK1 includes the casein kinase 1 kinases | NONE | NONE | NONE | LOC_Os05g51560 | Captured by EnSpm-13_ZM\|DNA/En-Spm |
| ZM-20 | Expressed | Erythronate-4-phosphate dehydrogenase domain containing protein | GRMZM2G176977_T01;  GRMZM2G176977_T02;  GRMZM2G176977_T03;  GRMZM2G176977_T04;  GRMZM2G176977_T05 | PF02826 | 2-Hacid_dh_C | LOC_Os07g16040 |  |

The light blue means the orthologous genes in rice.
