# Supplementary material for: Comparative Sequence Analysis of the Ghd7 Orthologous Regions Revealed Movement of Ghd7 in the Grass Genomes
Source: PLoS One. 2012 Nov 21;7(11):e50236. doi: 10.1371/journal.pone.0050236 (PMC3503983; doi:10.1371/journal.pone.0050236)
Supplement: Table S9 — Annotation of intact DNA transposable elements. (DOCX) [file pone.0050236.s013.docx]

**Table S9** Annotation of intact DNA transposable elements.

|  | *O. sativa* L. ssp. j*aponica* | *O. sativa* L. ssp. *indica* | *O. glaberrima* | *O. rufipogon* | *O. nivara* | *O. glumaepatula* | *O. punctata* | *O. officinalis* | *O. australiensis* | *O. brachyantha* | *B. distachyon* | *S. bicolor* | *Z. mays* |
| --- | --- | --- | --- | --- | --- | --- | --- | --- | --- | --- | --- | --- | --- |
|  |  |  |  |  |  |  |  |  |  |  |  |  |  |
| MITEs/Tourist | 20 | 17 | 17 | 10 | 7 | 17 | 7 | 2 | 1 | 5 | 0 | 9 | 0 |
| MITEs/Stowaway | 24 | 24 | 23 | 17 | 12 | 17 | 2 | 3 | 4 | 6 | 0 | 15 | 0 |
| MuDR | 14 | 8 | 10 | 5 | 5 | 11 | 2 | 2 | 0 | 4 | 0 | 0 | 1 |
| En-Spm | 7 | 5 | 6 | 4 | 4 | 2 | 2 | 6 | 6 | 0 | 0 | 24 | 0 |
| SEVERIN-2 | 4 | 1 | 1 | 1 | 0 | 2 | 0 | 0 | 0 | 0 | 0 | 0 | 0 |
| Others | 6 | 5 | 5 | 2 | 0 | 4 | 1 | 1 | 2 | 2 | 0 | 2 | 6 |
| BAC sequences occupied by intact TE (%) | 10.48 | 6.01 | 7.55 | 7.02 | 9.02 | 9.05 | 3.49 | 11.17 | 9.53 | 2.04 | 0 | 6.03 | 1.05 |
| Total (%) | 15.31 | 11.66 | 12.63 | 11.49 | 12.49 | 13.31 | 11.63 | 21.63 | 11.21 | 7.03 | 0.79 | 16.57 | 5.16 |
