# Supplementary material for: Comparative Sequence Analysis of the Ghd7 Orthologous Regions Revealed Movement of Ghd7 in the Grass Genomes
Source: PLoS One. 2012 Nov 21;7(11):e50236. doi: 10.1371/journal.pone.0050236 (PMC3503983; doi:10.1371/journal.pone.0050236)
Supplement: Table S10 — List of intact retrotransposons, solo-LTRs and their conservation in Oryza species. (DOCX) [file pone.0050236.s014.docx]

**Table S10** List of intact retrotransposons, solo-LTRs and their conservation in *Oryza* species.

|  | | Begin | End | F/R |  |  | TSD | Ks | Insertion time (Mya) | Conservation |
| --- | --- | --- | --- | --- | --- | --- | --- | --- | --- | --- |
| ***O. sativa* L. ssp. *japonica*** | |  |  |  |  |  |  |  |  |  |
| SZ-10old_LTR\|LTR/Copia | | 121189 | 132938 |  | AUTO | RNA | ATGAC | 0.0294 | 1.13 | 1,2,4 |
| SZ-57\|LTR/Copia | | 161340 | 166711 |  | AUTO | RNA | ATATA | 0.0181 | 0.70 | 1,2,3,4,6 |
| RETRO2 \|LTR/Gypsy | | 202982 | 215883 |  | AUTO | RNA | TTAAC | 0.0087 | 0.33 | 1 |
| ATLANTYS_OS\|LTR/Gypsy | | 280218 | 297135 |  | NESTED | RNA | ACTAT | 0.0293 | 1.13 | solo in 3 |
| RIRE1 \|LTR/Copia | | 376562 | 384208 | C | AUTO | RNA | GCTGC /GCTAC | 0.0465 | 1.79 | 1,2,3,4,5 |
| RETROSOR2\|LTR/Gypsy | | 506043 | 510652 |  | AUTO | RNA | ATGCC | 0.0322 | 1.24 | 1,3 |
| SZ-33\|LTR/Gypsy | | 516163 | 541510 | C | NESTED | RNA | GAGGG | 0.0691 | 2.66 | 1,3 |
| RIREX \|LTR/Gypsy | | 521425 | 535350 |  | NESTED | RNA | GTGAG | 0.0515 | 1.98 | 1,truncated in 2,3 |
| RIREX \|LTR/Gypsy | | 521713 | 529117 | C | AUTO | RNA | TATGA | 0.0049 | 0.19 | 1 |
| SINE9_OS\|SINE | | 17273 | 17566 | C | SINE | RNA | AAATTGTTATGTAGCAA/AAATTGTTATATAGCAA |  |  | 1,2,3 |
| SINE9_OS\|SINE | | 18857 | 19134 |  | SINE | RNA | TAGACGGTACGA/TAGGACGGTACGA |  |  | 1,2,3 |
| SINE1R5_OS\|SINE | | 39936 | 40055 | C | SINE | RNA | TTTTATG/TTTTCTC |  |  | 1,2,3 |
| SINE03_OS\|SINE | | 188216 | 188572 |  | SINE | RNA | AT |  |  | 1,2,3,4,5,6 |
| SINE9_OS\|SINE | | 399906 | 400114 | C | SINE | RNA | TGGCT |  |  | 1,2,3,4,6 |
| SINE03_OS\|SINE | | 542319 | 542583 |  | SINE | RNA | TAA |  |  | 1,2,3 |
| COPI1_LTR\|LTR/Copia | | 10221 | 10535 | C | SOLO | RNA | GCTTC |  |  | 1 |
| Atlantys-2-LTR_OS\|LTR/Gypsy | | 29752 | 30738 |  | SOLO | RNA | GCGTC |  |  | 1,2,3 |
| ATLANTYS-LTR_OS\|LTR/Gypsy | | 57490 | 58717 | C | SOLO | RNA | CCAG |  |  | 1,2,truncated in 3 |
| SZ-66LTR\|LTR/Gypsy | | 95464 | 96690 |  | SOLO | RNA | GGTT/GATT |  |  | 1,2,3 |
| SZ-31_LTR\|LTR/Gypsy | | 108430 | 109330 |  | SOLO | RNA | TAT/TAG |  |  | 1,2,truncated in 3 |
| Atlantys-2-LTR_OS\|LTR/Gypsy | | 157759 | 158744 | C | SOLO | RNA | CACCC/CACCT |  |  | 1,2,3,4,6 |
| BAJIE_LTR\|LTR/Gypsy | | 177905 | 178577 | C | SOLO | RNA | AGAGG |  |  | 1,2,3,4,5,6 |
| COPIA1-LTR_OS\|LTR/Copia | | 194109 | 195247 |  | SOLO | RNA | ACCAC/CTCTC |  |  | 1,2,4,5,6 |
| RIRE3A_LTR\|LTR/Gypsy | | 226017 | 229250 |  | SOLO | RNA | TTTT |  |  | 1,2,3,4,5,6 |
| RIRE1_LTR\|LTR/Copia | | 232049 | 233438 |  | SOLO | RNA | GTAGA |  |  | 1,2,3,4,5,6 |
| RIRE3_LTR\|LTR/Gypsy | | 270836 | 276711 | C | SOLO | RNA | AGGAA |  |  | 1 |
| RIRE3_LTR\|LTR/Gypsy | | 280626 | 283776 |  | SOLO | RNA | TAAAT |  |  | 1 |
| SZ-5LIKE\|LTR/Copia | | 326097 | 327179 | C | SOLO | RNA | TTCAC/CAGTG |  |  | 1,2,3,4,5,6 |
| SZ-42_LTR\|LTR/Gypsy | | 340641 | 341577 | C | SOLO | RNA | TATA/GAGG |  |  | 1,2,3,4,5 |
| SZ-66LTR\|LTR/Gypsy | | 342605 | 343842 |  | SOLO | RNA | ATAGG |  |  | 1,2,3,4,5 |
| SZ-61_LTR\|LTR/Copia | | 387510 | 393696 |  | SOLO | RNA | TAACC/CAACC |  |  | 1,3,truncated in 2,4,5 |
| SZ-35_LTR\|LTR/Gypsy | | 387804 | 391530 | C | SOLO | RNA | CATGC |  |  | 1,2,3,truncated in 4,5 |
| SZ-36_LTR\|LTR | | 426592 | 432447 | C | SOLO | RNA | CATTT |  |  | 1 |
| COPIA2-LTR_OS\|LTR/Copia | | 457171 | 458144 |  | SOLO | RNA | GTTGT/ATTGT |  |  | 1,2,3,6 |
| ***O. sativa* L. ssp. *indica*** | |  |  |  |  |  |  |  |  |  |
| SZ-10old_LTR\|LTR/Copia | | 101899 | 113259 |  | NESTED | RNA | ATGAC | 0 | 0.00 |  |
| TRUNCATOR2_OS\|LTR/Gypsy | | 108560 | 111411 |  | AUTO | RNA | GGCAG/GATTC |  |  | 2 |
| SZ-57\|LTR/Copia | | 144091 | 149456 |  | AUTO | RNA | ATATA | 0.013 | 0.50 |  |
| TRUNCATOR2_OS\|LTR/Gypsy | | 265638 | 268732 |  | AUTO | RNA | CAGAA |  |  | 2,4,5,truncated in 1 |
| COPIA2_OS\|LTR/Copia | | 289957 | 296434 | C | AUTO | RNA | ATCTC | 0.0755 | 2.90 | 2,5,6,truncated in 1,3,4 |
| RIRE1\|LTR/Copia | | 367573 | 375200 | C | AUTO | RNA | CGTCG | 0.0534 | 2.05 |  |
| TRUNCATOR2_OS\|LTR/Gypsy | | 488102 | 501631 |  | NESTED | RNA | TGAAC |  |  | 2 |
| SINE9_OS\|SINE | | 778 | 1075 | C | SINE | RNA | AAATTGTTATGTAGCAA/AAATTGTTATATAGCAA |  |  |  |
| SINE9_OS\|SINE | | 2364 | 2640 |  | SINE | RNA | TAGACGGTACGA/TAGGACGGTACGA |  |  |  |
| SINE1R5_OS\|SINE | | 23345 | 23464 | C | SINE | RNA | TTTTATG/TTTTCTC |  |  |  |
| SINE03_OS\|SINE | | 171991 | 172340 |  | SINE | RNA | AT |  |  |  |
| SINE9_OS\|SINE | | 390630 | 390760 | C | SINE | RNA | TGGCT |  |  |  |
| SINE03_OS\|SINE | | 508560 | 508812 |  | SINE | RNA | TAA |  |  |  |
| Atlantys-2_OS\|LTR/Gypsy | | 13146 | 14142 |  | SOLO | RNA | GCGTC |  |  |  |
| ATLANTYS-LTR_OS\|LTR/Gypsy | | 40986 | 42212 | C | SOLO | RNA | CCAGC |  |  |  |
| SZ-66LTR\|LTR/Gypsy | | 74652 | 75896 |  | SOLO | RNA | GGATT/ATGGA |  |  |  |
| SZ-31_LTR\|LTR/Gypsy | | 87349 | 88252 |  | SOLO | RNA | CTAC |  |  |  |
| Atlantys-2-LTR_OS\|LTR/Gypsy | | 137443 | 141509 | C | SOLO | RNA | CACCC/CACCT |  |  |  |
| RETROSAT2LTRA\|LTR/Gypsy | | 138350 | 141425 |  | SOLO | RNA | GAGGG |  |  | 2,4 |
| BAJIE_LTR\|LTR/Gypsy | | 161258 | 161930 | C | SOLO | RNA | AGAGG |  |  |  |
| COPIA1-LTR_OS\|LTR/Copia | | 194828 | 195966 |  | SOLO | RNA | ACCAC/CTCTC |  |  |  |
| RIRE3A_LTR\|LTR/Gypsy | | 214195 | 217442 |  | SOLO | RNA | TTTT |  |  |  |
| RIRE1_LTR\|LTR/Copia | | 220243 | 221641 |  | SOLO | RNA | GTAAA/GTAGA |  |  |  |
| SC-9_LTR\|LTR/Copia | | 301194 | 301650 | C | SOLO | RNA | CTTAT |  |  | 2 |
| ATLANTYS-LTR_OS\|LTR/Gypsy | | 307511 | 309026 |  | SOLO | RNA | GTACT |  |  | 2 |
| SZ-5LIKE\|LTR/Copia | | 315902 | 317015 | C | SOLO | RNA | TTCAC/CAGTG |  |  |  |
| SZ-42_LTR\|LTR/Gypsy | | 330535 | 331471 | C | SOLO | RNA | TATAC/GAGGA |  |  |  |
| SZ-66LTR\|LTR/Gypsy | | 332499 | 333736 |  | SOLO | RNA | ATAGG |  |  |  |
| SZ-35_LTR\|LTR/Gypsy | | 378722 | 383870 | C | SOLO | RNA | CATGC |  |  |  |
| COPIA2-LTR_OS\|LTR/Copia | | 425354 | 426309 |  | SOLO | RNA | GTTGT/ATTGT |  |  |  |
| ***O. glaberrima*** | |  |  |  |  |  |  |  |  |  |
| SZ-57\|LTR/Copia | | 167709 | 173486 |  | AUTO | RNA | ATATA | 0.0183 | 0.70 |  |
| SZ-19_LTR and ATLANTYS-I_OS-int\|LTR/Gypsy | | 229342 | 252024 | C | NESTED | RNA | GATAG | 0.0481 | 1.85 | 3,solo in 6 |
| GYPSY1-LTR_OS\|LTR/Gypsy | | 234277 | 243130 | C | AUTO | RNA | GAAAA | 0.0848 | 3.26 | 3 |
| RIRE1 \|LTR/Copia | | 365866 | 373567 | C | AUTO | RNA | CGTCG | 0.049 | 1.88 |  |
| RETROSOR2\|LTR/Gypsy | | 468533 | 472852 |  | AUTO | RNA | ATGCC | 0.0269 | 1.03 |  |
| SZ-7 and SZ-33\|LTR/Gypsy | | 478363 | 496908 | C | NESTED | RNA | GAGGG | 0.0568 | 2.18 |  |
| RIREX \|LTR/Gypsy | | 483698 | 490688 |  | AUTO | RNA | GTGAG | 0.0495 | 1.90 |  |
| SINE9_OS\|SINE | | 70 | 361 | C | SINE | RNA | AAATTGTTATGTAGCA/AAATTGTTATATAGCA |  |  |  |
| SINE9_OS\|SINE | | 1652 | 1930 |  | SINE | RNA | TAGACGGTACGA/TAGGACGGTACGA |  |  |  |
| SINE1R5_OS\|SINE | | 32978 | 33097 | C | SINE | RNA | TTTTATG/TTTTCTC |  |  |  |
| SINE03_OS\|SINE | | 194928 | 195279 |  | SINE | RNA | AT |  |  |  |
| SINE9_OS\|SINE | | 389349 | 389557 | C | SINE | RNA | TGGCT |  |  |  |
| SINE03_OS\|SINE | | 497704 | 497928 |  | SINE | RNA | TAA |  |  |  |
| Atlantys-2-LTR_OS\|LTR/Gypsy | | 12390 | 13385 |  | SOLO | RNA | GCGTC |  |  |  |
| SZ-38_LTR\|LTR/Gypsy | | 103001 | 104496 | C | SOLO | RNA | TGGGT/TGGGC |  |  | 3,truncated in 1,2 |
| SZ-66_LTR\|LTR/Gypsy | | 104988 | 105324 |  | SOLO | RNA | GGACT/ATGGA |  |  |  |
| SZ-43_LTR\|LTR/Gypsy | | 112355 | 116487 |  | SOLO | RNA | GCGTC/GCGCC |  |  | 3 |
| Atlantys-2-LTR_OS\|LTR/Gypsy | | 157545 | 158528 | C | SOLO | RNA | CACCC/CACCT |  |  |  |
| BAJIE_LTR\|LTR/Gypsy | | 184617 | 185289 | C | SOLO | RNA | AGAGG |  |  |  |
| RIRE3A_LTR\|LTR/Gypsy | | 219835 | 223068 |  | SOLO | RNA | TTTT |  |  |  |
| RIRE1_LTR\|LTR/Copia | | 225860 | 227240 |  | SOLO | RNA | GTAGA |  |  |  |
| SZ-43_LTR\|LTR/Gypsy | | 257400 | 261654 |  | SOLO | RNA | TGGTG |  |  | 3,6 |
| ATLANTYS-LTR_OS\|LTR/Gypsy | | 295668 | 296983 |  | SOLO | RNA | ACTAT |  |  |  |
| SZ-5LIKE\|LTR/Copia | | 315221 | 316313 | C | SOLO | RNA | TTCAC |  |  |  |
| SZ-42_LTR\|LTR/Gypsy | | 329904 | 330840 | C | SOLO | RNA | TATAC/GAGGA |  |  |  |
| SZ-66LTR\|LTR/Gypsy | | 331870 | 333107 |  | SOLO | RNA | ATAGG |  |  |  |
| SZ-61_LTR\|LTR/Copia | | 376780 | 383125 |  | SOLO | RNA | CAACC |  |  |  |
| SZ-35_LTR\|LTR/Gypsy | | 377064 | 380965 | C | SOLO | RNA | CATGC |  |  |  |
| RIREX_LTR\|LTR/Gypsy | | 489570 | 490013 |  | SOLO | RNA | GCTAC/CCCTG |  |  | 3 |
| COPIA2-LTR_OS\|LTR/Copia | | 439845 | 440813 |  | SOLO | RNA | GTTGT/ATTGT |  |  |  |
| ***O. rufipogon*** | |  |  |  |  |  |  |  |  |  |
| SZ-10old_LTR\|LTR/Copia | | 4856 | 16640 |  | AUTO | RNA | ATGAC | 0.0287 | 1.10 |  |
| SZ-57_LTR\|LTR/Copia | | 47776 | 53142 |  | AUTO | RNA | ATATA | 0.013 | 0.50 |  |
| RETRO2 \|LTR/Gypsy | | 67123 | 71806 |  | AUTO | RNA | ATACC | 0.0166 | 0.64 | 4 |
| TRUNCATOR\|LTR/Gypsy | | 74801 | 77807 | C | AUTO | RNA | TCACT |  |  | 4,5 |
| TRUNCATOR2_OS\|LTR/Gypsy | | 158122 | 161252 |  | AUTO | RNA | CAGAC |  |  | 4,5 |
| TRUNCATOR2_OS\|LTR/Gypsy | | 167371 | 170220 |  | AUTO | RNA | CCCGG |  |  | 4,5 |
| TRUNCATOR2_OS\|LTR/Gypsy | | 176566 | 179519 |  | AUTO | RNA | GAAAC |  |  | 4 |
| RIRE3\|LTR/Gypsy | | 251947 | 263995 | C | AUTO | RNA | NNNNN | 0.1172 | 4.51 | 4 |
| TRUNCATOR2_OS\|LTR/Gypsy | | 279331 | 282220 | C | AUTO | RNA | TCCAA/TCAA |  |  | 4 |
| RIRE1\|LTR/Copia | | 333313 | 340119 | C | AUTO | RNA | CGTCG | 0.0634 | 2.44 |  |
| TRUNCATOR2_OS\|LTR/Gypsy | | 351627 | 353969 | C | AUTO | RNA | GGGTT |  |  | 4 |
| RETRO2\|LTR/Gypsy | | 374085 | 387192 | C | AUTO | RNA | CTCCA | 0.0137 | 0.53 | 4 |
| SINE03_OS\|SINE | | 86708 | 87059 |  | SINE | RNA | AT/TT |  |  |  |
| SINE9_OS\|SINE | | 371335 | 371544 | C | SINE | RNA | TGGCT |  |  |  |
| Atlantys-2-LTR_OS\|LTR/Gypsy | | 41156 | 45219 | C | SOLO | RNA | CACCT/CACCC |  |  |  |
| RETROSAT2LTRA\|LTR/Gypsy | | 42063 | 45135 |  | SOLO | RNA | GAGGG |  |  |  |
| BAJIE_LTR\|LTR/Gypsy | | 64373 | 65045 | C | SOLO | RNA | AGAGG |  |  |  |
| RETROSAT5_LTR\|LTR/Gypsy | | 65934 | 73720 | C | SOLO | RNA | TCAGG |  |  | 4,5 |
| COPIA1-LTR_OS\|LTR/Copia | | 92611 | 93749 |  | SOLO | RNA | ACCAC/ATCTC |  |  |  |
| RIRE3A_LTR\|LTR/Gypsy | | 112479 | 115729 |  | SOLO | RNA | TTTT |  |  |  |
| RIRE1_LTR\|LTR/Copia | | 118535 | 119938 |  | SOLO | RNA | GTAGA |  |  |  |
| RIRE3_LTR\|LTR/Gypsy | | 152867 | 156005 | C | SOLO | RNA | CTATG |  |  | 4 |
| SZ-5LIKE\|LTR/Copia | | 235147 | 236262 | C | SOLO | RNA | TTCAC/CCCAG |  |  |  |
| SZ-42_LTR\|LTR/Gypsy | | 249841 | 250777 | C | SOLO | RNA | GAGGA/TATAC |  |  |  |
| SZ-66LTR\|LTR/Gypsy | | 266557 | 267794 |  | SOLO | RNA | ATAGG |  |  |  |
| RIRE3\|LTR/Gypsy | | 297138 | 300456 |  | SOLO | RNA | ACCTC |  |  | 4 |
| RIRE3A\|LTR/Gypsy | | 360795 | 364812 | C | SOLO | RNA | CTCTC |  |  | 4 |
| ***O. nivara*** | |  |  |  |  |  |  |  |  |  |
| TRUNCATOR\|LTR/Gypsy | | 33347 | 36353 | C | AUTO | RNA | TCACT |  |  |  |
| TRUNCATOR2_OS\|LTR/Gypsy | | 119398 | 122351 | C | AUTO | RNA | GAAAC |  |  | 5 |
| TRUNCATOR2_OS\|LTR/Gypsy | | 129036 | 132166 |  | AUTO | RNA | CAGAC |  |  |  |
| ATLANTYS_OS\|LTR/Gypsy | | 136572 | 162828 |  | NESTED | RNA | ACTAT | 0.0349 | 1.34 |  |
| TRUNCATOR2_OS\|LTR/Gypsy | | 138285 | 141134 |  | AUTO | RNA | CCCGG |  |  |  |
| COPIA2_OS\|LTR/Copia | | 172984 | 176921 | C | AUTO | RNA | CTCTA | 0.1029 | 3.96 |  |
| RIRE1\|LTR/Copia | | 268838 | 275644 | C | AUTO | RNA | CGTCG | 0.0593 | 2.28 |  |
| TRUNCATOR2_OS\|LTR/Gypsy | | 331693 | 334035 |  | AUTO | RNA | TTGGG |  |  | 5 |
| SINE03_OS\|SINE | | 45251 | 45600 |  | SINE | RNA | AT/TT |  |  |  |
| BAJIE_LTR\|LTR/Gypsy | | 28544 | 29216 | C | SOLO | RNA | AGAGG |  |  |  |
| RETROSAT5_LTR\|LTR/Gypsy | | 30105 | 32266 | C | SOLO | RNA | TCAGG |  |  |  |
| COPIA1-LTR_OS\|LTR/Copia | | 51149 | 52287 |  | SOLO | RNA | ACCAC/CTCTC |  |  |  |
| RIRE3A_LTR\|LTR/Gypsy | | 70494 | 73744 |  | SOLO | RNA | TTTT |  |  |  |
| RIRE1_LTR\|LTR/Copia | | 76550 | 77953 |  | SOLO | RNA | GTAGA |  |  |  |
| SZ-5LIKE\|LTR/Copia | | 189090 | 190205 | C | SOLO | RNA | CCCAG/TTCAC |  |  |  |
| SZ-42_LTR\|LTR/Gypsy | | 203690 | 204626 | C | SOLO | RNA | GAGGA/TATAC |  |  |  |
| SZ-66LTR\|LTR/Gypsy | | 229533 | 230770 |  | SOLO | RNA | ATAGG |  |  |  |
| RIRE3A_LTR\|LTR/Gypsy | | 247708 | 248528 |  | SOLO | RNA | ACCGT |  |  | 5 |
| RIRE3_LTR\|LTR/Gypsy | | 290223 | 298525 |  | SOLO | RNA | ACCTA |  |  | 5 |
| RIRE3A_LTR\|LTR/Gypsy | | 290780 | 294796 |  | SOLO | RNA | CTCTC |  |  | 5 |
| ***O. glumaepatula*** | |  |  |  |  |  |  |  |  |  |
| SZ-57\|LTR/Copia | | 45133 | 50498 |  | AUTO | RNA | ATATA | 0.046 | 1.77 |  |
| ATLANTYS_OS\|LTR/Gypsy | | 183677 | 204715 |  | NESTED | RNA | ACTAT | 0.036 | 1.38 |  |
| TRUNCATOR\|LTR/Gypsy | | 187711 | 189960 |  | AUTO | RNA | AGTGG/NNNNN |  |  | 6 |
| COPIA2_OS\|LTR/Copia | | 214749 | 221174 | C | AUTO | RNA | CTCTA | 0.0577 | 2.22 |  |
| RIRE3_LTR\|LTR/Gypsy | | 225673 | 238806 |  | NESTED | RNA | TGCAT | 0.0332 | 1.28 | 6 |
| SINE03_OS\|SINE | | 89668 | 90010 |  | SINE | RNA | AT |  |  |  |
| SINE9_OS\|SINE | | 319566 | 319775 | C | SINE | RNA | TGGCT |  |  |  |
| Atlantys-2-LTR_OS\|LTR/Gypsy | | 41455 | 42442 | C | SOLO | RNA | CACCT |  |  |  |
| BAJIE_LTR\|LTR/Gypsy | | 61427 | 62099 | C | SOLO | RNA | AGAGG |  |  |  |
| COPIA1-LTR_OS\|LTR/Copia | | 95563 | 96701 |  | SOLO | RNA | ACCAC/TTCTC |  |  |  |
| RIRE3A_LTR\|LTR/Gypsy | | 114525 | 117757 |  | SOLO | RNA | TTTT |  |  |  |
| RIRE1_LTR\|LTR/Copia | | 120585 | 131170 |  | SOLO | RNA | GTAGA |  |  |  |
| SZ-19_LTR\|LTR/Gypsy | | 133254 | 134758 |  | SOLO | RNA | GATAG |  |  |  |
| SZ-43_LTR\|LTR/Gypsy | | 140172 | 144422 |  | SOLO | RNA | TGGTG |  |  |  |
| SZ-5LIKE\|LTR/Copia | | 222964 | 224077 | C | SOLO | RNA | CAGTG/TTCAC |  |  |  |
| RIRE5-LTR_OS\|LTR/Copia | | 244754 | 245670 | C | SOLO | RNA | CTTTC |  |  | 6 |
| SZ-42_LTR\|LTR/Gypsy | | 249869 | 250796 |  | SOLO | RNA | TCTAC/GTTGG |  |  | 6 |
| Gypsy-A_LTR\|LTR/Gypsy | | 304447 | 312328 |  | SOLO | RNA | ACTAA |  |  | 6 |
| SZ-62_LTR\|LTR/Gypsy | | 305839 | 310329 | C | SOLO | RNA | GAAGC |  |  | 6 |
| COPIA2-LTR_OS\|LTR/Copia | | 405541 | 406578 |  | SOLO | RNA | GTTGT/ATTGT |  |  |  |
| ***O. punctata*** | |  |  |  |  |  |  |  |  |  |
| ATLANTYS_OS\|LTR/Gypsy | | 8256 | 21442 |  | AUTO | RNA | GTTTG | 0.0283 | 1.09 |  |
| ATLANTYS_OS\|LTR/Gypsy | | 145441 | 149733 | C | AUTO | RNA | TCGAG | 0.0763 | 2.93 |  |
| TRUNCATOR\|LTR/Gypsy | | 157121 | 160084 | C | AUTO | RNA | GGGA |  |  |  |
| TRUNCATOR2_OS\|LTR/Gypsy | | 192253 | 195566 | C | AUTO | RNA | CATTT |  |  |  |
| LTR2-ZM\|LTR | | 198260 | 198924 | C | AUTO | RNA | TGGTG | 0.0346 | 1.33 |  |
| ATLANTYS_OS\|LTR/Gypsy | | 273692 | 284661 |  | AUTO | RNA | CCAAT/CCAGG | 0.0492 | 1.89 |  |
| VEJU1_TM-LTR\|LTR | | 397032 | 399028 | C | AUTO | RNA | ATCAA/AGCAA | 0.11 | 4.23 |  |
| RIRE2\|LTR/Gypsy | | 399617 | 410828 |  | AUTO | RNA | ACTAG | 0.0641 | 2.47 |  |
| SZ-50\|LTR/Gypsy | | 445623 | 456757 |  | AUTO | RNA | CCTGG/TCTGG | 0.0859 | 3.30 |  |
| RETROSOR2\|LTR/Gypsy | | 508443 | 520074 | C | AUTO | RNA | CTCAT | 0.0443 | 1.70 |  |
| CRMA1\|LTR/Gypsy | | 536196 | 543987 | C | AUTO | RNA | CAAAA | 0.0288 | 1.11 |  |
| ATLANTYS_OS\|LTR/Gypsy | | 580751 | 593429 |  | AUTO | RNA | GGTAT | 0.1085 | 4.17 |  |
| RIREX_LTR\|LTR/Gypsy | | 609171 | 615204 |  | AUTO | RNA | CTAAG/CTCAA | 0.0957 | 3.68 |  |
| ATLANTYS_OS\|LTR/Gypsy | | 573231 | 599036 | C | NESTED | RNA | TACAC | 0.0504 | 1.94 |  |
| ATLANTYS_OS\|LTR/Gypsy | | 42191 | 43696 | C | SOLO | RNA | CATGG |  |  |  |
| RIRE1_LTR\|LTR/Copia | | 50322 | 51705 |  | SOLO | RNA | CGTGT |  |  |  |
| ATLANTYS-LTR_OS\|LTR/Gypsy | | 58149 | 59614 |  | SOLO | RNA | GGATG/TGATG |  |  |  |
| SZ-20LTR\|LTR/Gypsy | | 163728 | 165203 | C | SOLO | RNA | CAGGA |  |  |  |
| RIREX_LTR\|LTR/Gypsy | | 167039 | 167476 | C | SOLO | RNA | ATGTG |  |  |  |
| ATLANTYS-LTR_OS\|LTR/Gypsy | | 175991 | 177439 |  | SOLO | RNA | GCACC |  |  |  |
| SZ-66LTR\|LTR/Gypsy | | 220985 | 222161 |  | SOLO | RNA | GCCGC/AGCAT |  |  |  |
| SZ-44_LTR\|LTR/Gypsy | | 428914 | 430447 |  | SOLO | RNA | ACTAG |  |  |  |
| RIRE1_LTR\|LTR/Copia | | 484871 | 486272 |  | SOLO | RNA | GATTT/GCTTT |  |  |  |
| SZ-44_LTR\|LTR/Gypsy | | 561593 | 562891 |  | SOLO | RNA | GTACC |  |  |  |
| SZ-20LTR\|LTR/Gypsy | | 623374 | 624809 |  | SOLO | RNA | GCAAC |  |  |  |
| COPI1_LTR\|LTR/Copia | | 638138 | 638408 |  | SOLO | RNA | CCATT |  |  |  |
| RIRE1_LTR\|LTR/Copia | | 642164 | 643565 |  | SOLO | RNA | AAATG |  |  |  |
| ***O. officinalis*** | |  |  |  |  |  |  |  |  |  |
| RETROSAT4_I-int/Gypsy SZ-35_LTR | | 133047 | 145037 | C | AUTO | RNA | CCTAA | 0.028 | 1.08 |  |
| RIRE2_I-int/RIREX_LTR\|LTR/Gypsy | | 294282 | 305492 |  | AUTO | RNA | CTTAG | 0.0262 | 1.01 |  |
| SINE9_OS\|SINE | | 177469 | 177724 |  | SINE | RNA | TAAAGCTATGGG/TAAAGCTAATGGG |  |  |  |
| SINE9_OS\|SINE | | 279279 | 279570 |  | SINE | RNA | GAGTCA/TTGTCA |  |  |  |
| ATLANTYS-LTR_OS\|LTR/Gypsy | | 7919 | 9503 | C | SOLO | RNA | ATGGT/CTATA |  |  |  |
| COPIA1-LTR_OS\|LTR/Copia | | 48199 | 49318 | C | SOLO | RNA | TGCGT |  |  |  |
| SZ-48_LTR\|LTR/Gypsy | | 65088 | 73983 | C | SOLO | RNA | AAGGA |  |  |  |
| SZ-45_LTR\|LTR/Gypsy | | 78849 | 80410 |  | SOLO | RNA | CGACT/AGCGG |  |  |  |
| COPIA1-LTR_OS\|LTR/Copia | | 83773 | 84891 |  | SOLO | RNA | CGTCT |  |  |  |
| SZ-14_LTR\|LTR/Gypsy | | 89171 | 91582 |  | SOLO | RNA | GAATC |  |  |  |
| Gypsy-A_LTR\|LTR/Gypsy | | 95979 | 99259 | C | SOLO | RNA | TACAT |  |  |  |
| SZ-5LIKE\|LTR/Copia | | 108883 | 110038 |  | SOLO | RNA | TTA |  |  |  |
| SZ-44_LTR\|LTR/Gypsy | | 150570 | 151901 |  | SOLO | RNA | GCATG/TCGGT |  |  |  |
| RETROSAT5_LTR\|LTR/Gypsy | | 288016 | 291127 |  | SOLO | RNA | CTGTG/CGACG |  |  |  |
| SZ-40_LTR\|LTR/Gypsy | | 407237 | 414212 | C | SOLO | RNA | AGATT/TGCTG |  |  |  |
| SZ-58_LTR\|LTR | | 421855 | 422248 | C | SOLO | RNA | GATCC |  |  |  |
| ***O. australiensis*** | |  |  |  |  |  |  |  |  |  |
| COPIA1_OS\|LTR/Copia | | 175877 | 184829 | C | AUTO | RNA | GTATG | 0.074 | 2.85 |  |
| RIREX\|LTR/Gypsy | | 248828 | 259264 |  | AUTO | RNA | ATAAG | 0.0113 | 0.43 |  |
| RIRE1\|LTR/Copia | | 264295 | 271459 |  | AUTO | RNA | CATCG | 0.0124 | 0.48 |  |
| RETROSAT2\|LTR/Gypsy | | 299719 | 308473 |  | AUTO | RNA | ATTGT | 0.0141 | 0.54 |  |
| RIRE2\|LTR/Copia | | 316622 | 323636 |  | AUTO | RNA | GGGAG | 0.0999 | 3.84 |  |
| GYPSY1_OS\|LTR/Gypsy | | 380092 | 385464 |  | AUTO | RNA | TATAG | 0.1323 | 5.09 |  |
| RIRE1\|LTR/Copia | | 402385 | 409768 |  | AUTO | RNA | AGAAG | 0.0234 | 0.90 |  |
| ATLANTYS_OS\|LTR/Gypsy | | 436571 | 451824 |  | NESTED | RNA | ATCGG/ATCGT | 0.057 | 2.19 |  |
| RIREX\|LTR/Gypsy | | 456427 | 467280 | C | AUTO | RNA | CATCT | 0.4184 | 16.09 |  |
| RIRE1\|LTR/Copia | | 527297 | 528903 | C | SOLO | RNA | TGAGA |  |  |  |
| RETROSAT3_LTR\|LTR/Copia | | 15751 | 18771 | C | SOLO | RNA | TTGAT |  |  |  |
| RIRE1_LTR\|LTR/Copia | | 49664 | 51276 |  | SOLO | RNA | TATGG/CATAT |  |  |  |
| SZ-21_LTR\|LTR/Gypsy | | 54234 | 55490 |  | SOLO | RNA | CCTGG |  |  |  |
| RIRE1_LTR\|LTR/Copia | | 190837 | 192409 | C | SOLO | RNA | ATTTT |  |  |  |
| ATLANTYS-LTR_OS\|LTR/Gypsy | | 244295 | 245778 | C | SOLO | RNA | CCACC |  |  |  |
| ATLANTYS-LTR_OS\|LTR/Gypsy | | 352735 | 354257 |  | SOLO | RNA | GATCC |  |  |  |
| RIRE1_LTR\|LTR/Copia | | 437840 | 439449 | C | SOLO | RNA | ATATA |  |  |  |
| SZ-36_LTR\|LTR | | 587091 | 591711 |  | SOLO | RNA | AAGTT |  |  |  |
| SZ-36_LTR\|LTR | | 595506 | 601377 | C | SOLO | RNA | TTTCG/TTCCG |  |  |  |
| RIRE1_LTR\|LTR/Copia | | 708080 | 709813 |  | SOLO | RNA | CTAGC |  |  |  |
| ***O. brachyantha*** | |  |  |  |  |  |  |  |  |  |
| SZ-30_LTR\|LTR/Copia | | 229899 | 232601 |  | SOLO | RNA | CTCTC |  |  |  |
| COPIA2-LTR_OS\|LTR/Copia | | 238813 | 239737 | C | SOLO | RNA | ATTAG |  |  |  |
| ***B. distachyon*** | |  |  |  |  |  |  |  |  |  |
| COPIA3-I_OS\|LTR/Copia | | 26675 | 29200 |  | AUTO | RNA | TTTTA | 0.0778 | 2.99 |  |
| ***S. bicolor*** | |  |  |  |  |  |  |  |  |  |
| Gypsy2-SB\|LTR/Gypsy | | 30761 | 33524 |  | AUTO | RNA | TAGTC | 0.0044 | 0.17 |  |
| Gypsy20_ZM\|LTR/Gypsy | | 112894 | 117934 |  | AUTO | RNA | AGAAG | 0.0109 | 0.42 |  |
| TEKAY_ZM\|LTR/Gypsy | | 198069 | 209717 | C | AUTO | RNA | AGCAG | 0.0044 | 0.17 |  |
| Gypsy2-SB\|LTR/Gypsy | | 374084 | 386118 | C | AUTO | RNA | TTGCG | 0.0046 | 0.18 |  |
| Copia3-SB\|LTR/Gypsy | | 444784 | 449863 | C | AUTO | RNA | TCATG | 0.0062 | 0.24 |  |
| Copia5-SB\|LTR/Copia | | 480684 | 492907 | C | AUTO | RNA | CTCCA | 0.0087 | 0.33 |  |
| Copia29-ZM \|LTR/Copia | | 502461 | 522321 | C | AUTO | RNA | TTATA | 0.0046 | 0.18 |  |
| Gypsy65-ZM \|LTR/Gypsy | | 507292 | 521449 | C | AUTO | RNA | GCGCC | 0.009 | 0.35 |  |
| Copia10-ZM \|LTR/Copia | | 540502 | 544844 |  | AUTO | RNA | ATGAT | 0.0913 | 3.51 |  |
| Gypsy-80_ZM\| LTR/Gypsy | | 582409 | 594146 | C | AUTO | RNA | TGTCA | 0.0314 | 1.21 |  |
| Copia5-SB\|LTR/Copia | | 621377 | 635210 | C | NESTED | RNA | TCTTA | 0.0249 | 0.96 |  |
| Copia5-SB\|LTR/Copia | | 622508 | 631550 |  | AUTO | RNA | GTAAT | 0.0114 | 0.44 |  |
| Gypsy2-SB\|LTR/Gypsy | | 662190 | 669851 |  | AUTO | RNA | ATATT | 0 | 0.00 |  |
| Copia15-ZM\|LTR/Copia | | 725554 | 737694 | C | AUTO | RNA | GAATA | 0.0114 | 0.44 |  |
| Copia22-ZM\|LTR/Copia | | 747797 | 758501 |  | AUTO | RNA | CCATC | 0.0168 | 0.65 |  |
| Gypsy2-SB\|LTR/Gypsy | | 787980 | 800002 | C | AUTO | RNA | TGTTA | 0 | 0.00 |  |
| Gypsy7-ZM\|LTR/Gypsy | | 809529 | 811192 |  | AUTO | RNA | CTAAG/GTAAG | 0.0273 | 1.05 |  |
| Copia18-ZM\|LTR/Copia | | 825954 | 831148 | C | AUTO | RNA | ACTT | 0.0245 | 0.94 |  |
| Gypsy2-SB\|LTR/Gypsy | | 865194 | 867866 | C | AUTO | RNA | ATATG | 0.0074 | 0.28 |  |
| Copia9-ZM\|LTR/Copia | | 885874 | 900478 |  | AUTO | RNA | CACTG | 0.0142 | 0.55 |  |
| Gypsy2-SB\|LTR/Gypsy | | 948823 | 962066 |  | AUTO | RNA | GCAGT | 0.0149 | 0.57 |  |
| Gypsy2-SB\|LTR/Gypsy | | 950662 | 958669 | C | AUTO | RNA | GGGATA | 0.0132 | 0.51 |  |
| Gypsy2-SB\|LTR/Gypsy | | 955433 | 958262 | C | AUTO | RNA | CAACC | 0.0257 | 0.99 |  |
| Copia23-ZM\|LTR/Copia | | 991882 | 997036 |  | AUTO | RNA | GAGAA | 0.022 | 0.85 |  |
| Copia2_ZM\|LTR/Copia | | 1140946 | 1147666 |  | AUTO | RNA | TTCGG/TTCAA | 0.1572 | 6.05 |  |
| Copia29-ZM\|LTR/Copia | | 1291149 | 1296641 | C | AUTO | RNA | TTGGC | 0.0191 | 0.73 |  |
| GYPSOR1_LTR\|LTR/Gypsy | | 1393727 | 1403504 | C | AUTO | RNA | GAAAG | 0.0244 | 0.94 |  |
| Copia5_SB\|LTR/Copia | | 1411727 | 1423882 | C | AUTO | RNA | CCGGC | 0.0182 | 0.70 |  |
| Gypsy2-SB_LTR\|LTR/Gypsy | | 1433725 | 1445788 |  | AUTO | RNA | GTCCT | 0 | 0.00 |  |
| ATLANTYS_OS\|LTR/Gypsy | | 1472239 | 1484600 | C | AUTO | RNA | AGTTG | 0.0074 | 0.28 |  |
| Copia-39_ZM\|LTR/Copia | | 1543545 | 1549936 | C | AUTO | RNA | ACGGA | 0.0262 | 1.01 |  |
| Gypsy2-SB_LTR\|LTR/Gypsy | | 1607443 | 1619020 |  | AUTO | RNA | ATATG | 0.0205 | 0.79 |  |
| SZ-38\|LTR/Gypsy | | 1748916 | 1756938 |  | AUTO | RNA | ATCAC | 0.0357 | 1.37 |  |
| SZ-38_LTR\|LTR/Gypsy | | 1768015 | 1775783 | C | AUTO | RNA | ATCAG | 0.0433 | 1.67 |  |
| GYPSOR1\|LTR/Gypsy | | 1791147 | 1798926 | C | AUTO | RNA | TTTTA | 0.0201 | 0.77 |  |
| GypsO\|LTR/Gypsy | | 1806509 | 1815785 |  | AUTO | RNA | GGTGA | 0.0137 | 0.53 |  |
| GypsO\|LTR/Gypsy | | 1865547 | 1873943 |  | AUTO | RNA | CACTG | 0.0173 | 0.67 |  |
| Gypsy2-SB\|LTR/Gypsy | | 1900741 | 1903348 | C | AUTO | RNA | CGTAA | 0.005 | 0.19 |  |
| GYPSOR1\|LTR/Gypsy | | 1918247 | 1930116 | C | AUTO | RNA | GGTTA | 0.018 | 0.69 |  |
| Gypsy3-SB\|LTR/Gypsy | | 1959576 | 1973103 | C | AUTO | RNA | GTATA | 0.0045 | 0.17 |  |
| RETROSOR1_SB\|LTR/Gypsy | | 2009871 | 2023384 |  | AUTO | RNA | TGCTT | 0.0025 | 0.10 |  |
| SZ-38\|LTR/Gypsy | | 2045209 | 2054071 |  | AUTO | RNA | TCAA | 0.0344 | 1.32 |  |
| ATLANTYS_OS\|LTR/Gypsy | | 2064470 | 2077317 | C | AUTO | RNA | CCAAC | 0.0352 | 1.35 |  |
| SC-4\|LTR/Copia | | 2085327 | 2090524 | C | AUTO | RNA | CGGAA | 0 | 0.00 |  |
| F524\|SINE | | 6583 | 6880 | C | SINE | RNA | TCACAAACATC |  |  |  |
| SZ-66LTR\|LTR/Gypsy | | 34731 | 35928 | C | SOLO | RNA | GAGAA |  |  |  |
| Copia22-ZM_LTR\|LTR/Copia | | 575150 | 577484 |  | SOLO | RNA | GTTGT/GTTAT |  |  |  |
| Copia22-ZM_LTR\|LTR/Copia | | 689770 | 692163 |  | SOLO | RNA | CACA |  |  |  |
| Copia14-ZM_LTR\|LTR/Copia | | 1541042 | 1543118 |  | SOLO | RNA | CCTTG |  |  |  |
| Copia14-ZM_LTR\|LTR/Copia | | 1575106 | 1577422 |  | SOLO | RNA | GCTTC |  |  |  |
| ***Z. mays*** | |  |  |  |  |  |  |  |  |  |
| Copia13-ZM \|LTR/Copia | | 8162 | 16159 | C | AUTO | RNA | TAATC | 0.0101 | 0.39 |  |
| LTR2-ZM\|LTR | | 34348 | 35155 |  | AUTO | RNA | TCCGG | 0.0592 | 2.28 |  |
| PREM4_ZM_LTR\|LTR/Copia | | 46884 | 54798 |  | AUTO | RNA | CTAGT | 0.0253 | 0.97 |  |
| XILON1_ZM \|LTR/Gypsy | | 139358 | 150276 | C | AUTO | RNA | GAACA | 0.0031 | 0.12 |  |
| PREM2_ZM\|LTR/Copia | | 163923 | 173297 | C | AUTO | RNA | CACCG | 0.0318 | 1.22 |  |
| XILON1_ZM\|LTR/Gypsy | | 225991 | 236788 | C | AUTO | RNA | GTACC | 0.0085 | 0.33 |  |
| Copia7-ZM\|LTR/Copia | | 266295 | 274142 | C | AUTO | RNA | GACCC | 0.0072 | 0.28 |  |
| Gypsy-76_ZM\|LTR/Gypsy | | 293628 | 301039 | C | AUTO | RNA | GGCTG | 0.0108 | 0.42 |  |
| Gypsy-67_ZM\|LTR/Gypsy | | 311716 | 322462 | C | AUTO | RNA | AGGCG | 0.007 | 0.27 |  |
| HUCK1_ZM\|LTR/Gypsy | | 339226 | 352859 | C | AUTO | RNA | CTTTA | 0.0124 | 0.48 |  |
| Copia20-ZM\|LTR/Copia | | 360618 | 371488 |  | AUTO | RNA | CATAC | 0.0502 | 1.93 |  |
| PREM2_ZM\|LTR/Copia | | 418349 | 427170 | C | AUTO | RNA | TGGTA | 0.0398 | 1.53 |  |
| Gypsy-74 OR HUCK1_ZM\|LTR/Gypsy | | 440181 | 453725 | C | AUTO | RNA | CGTGA | 0.0416 | 1.60 |  |
| HUCK1_ZM\|LTR/Gypsy | | 476419 | 487419 |  | AUTO | RNA | AATAG | 0.0364 | 1.40 |  |
| Gypsy-73_ZM\|LTR/Gypsy | | 283349 | 289306 | C | AUTO | RNA | GACCC | 0.0039 | 0.15 |  |
| Gypsy-74-LTR_ZM\|LTR/Gypsy | | 259533 | 306428 | C | NESTED | RNA | TGCAA | 0.0416 | 1.60 |  |
| HUCK1_ZM\|LTR/Gypsy | | 265141 | 289999 | C | NESTED | RNA | TGAAA | 0.039 | 1.50 |  |
| Copia20-ZM\|LTR/Copia | | 339154 | 363279 | C | NESTED | RNA | GCTTC | 0.0488 | 1.88 |  |
| LTR2-ZM\|LTR | | 418067 | 427701 | C | NESTED | RNA | TATAC | 0 | 0.00 |  |
| OPIE2_ZM-LTR\|LTR/Copia | | 150787 | 152002 | C | SOLO | RNA | GGTTA/TAGAG |  |  |  |
| HUCK1-LTR_ZM\|LTR/Gypsy | | 194203 | 196137 |  | SOLO | RNA | CCTT |  |  |  |
| PREM3_ZM-LTR\|LTR/Copia | | 212903 | 214199 | C | SOLO | RNA | GGTTA/CATAC |  |  |  |
| PREM3_ZM-LTR\|LTR/Copia | | 246259 | 247516 |  | SOLO | RNA | GAGAT/CATAC |  |  |  |
| Gypsy-73-LTR_ZM\|LTR/Gypsy | | 353193 | 354797 |  | SOLO | RNA | CTCTC |  |  |  |
| 1- *O.sativa* L. ssp. *japonica* | |  |  |  |  |  |  |  |  |  |
| 2- *O. sativa* L. ssp. *indica* | |  |  |  |  |  |  |  |  |  |
| 3- *O. glaberrima* | |  |  |  |  |  |  |  |  |  |
| 4- *O. rufipogon* | |  |  |  |  |  |  |  |  |  |
| 5- *O. nivara* | |  |  |  |  |  |  |  |  |  |
| 6- *O. glumaepatula* | |  |  |  |  |  |  |  |  |  |
