# Supplementary material for: Comparative Sequence Analysis of the Ghd7 Orthologous Regions Revealed Movement of Ghd7 in the Grass Genomes
Source: PLoS One. 2012 Nov 21;7(11):e50236. doi: 10.1371/journal.pone.0050236 (PMC3503983; doi:10.1371/journal.pone.0050236)
Supplement: Table S11 — List of intact DNA transposons and their conservation in Oryza species. (DOCX) [file pone.0050236.s015.docx]

**Table S11** List of intact DNA transposons and their conservation in *Oryza* species.

| Species | | Begin | | End | F/R |  | Type | TSD | Conservation | |
| --- | --- | --- | --- | --- | --- | --- | --- | --- | --- | --- |
| ***O. sativa* L. ssp. *japonica*** | |  | |  |  |  |  |  |  | |
| STOWAWAY1_OS\|DNA/TcMar-Stowaway | | 6048 | | 6204 | C | NON | DNA | ATT | Non-coverage in other AA genome | |
| DITTO/3\|DNA/Tourist | | 14081 | | 15104 | C | NESTED | DNA | ATT |  |  |
| MUDRN1_OS\|DNA/MuDR | | 14288 | | 15058 | C | NON | DNA | ATCTAT |  |  |
| ZM13-1_OS\|DNA/Tourist | | 17937 | | 18065 |  | NON | DNA | TA | 1,2,3 |  |
| TREP215\|DNA/TcMar-Stowaway | | 22166 | | 22405 |  | NON | DNA | TA | 1,2 |  |
| UNIQUE\|DNA/Tourist | | 32847 | | 32995 | C | NON | DNA | TAT | 1,2 |  |
| TNR2_OR\|DNA | | 34140 | | 34298 |  | NON | DNA | ACGTTATTCGG/ ACGTTATTCTT | 1,2,3 |  |
| STOWAWAY11_OS\|DNA/TcMar-Stowaway | | 44072 | | 44359 |  | NON | DNA | TA | 1,2,3 |  |
| MERMITE\|DNA/MuDR | | 47033 | | 48475 |  | NON | DNA | ATCAAGAGG | 1 |  |
| CACTA-H\|DNA/En-Spm | | 49150 | | 50193 |  | NON | DNA | CTAT | 1,3; gap in 2 | |
| F770\|DNA/Tourist | | 92008 | | 92294 |  | NON | DNA | ATA | 1,3 |  |
| ENSPM4_OS\|DNA/En-Spm | | 103442 | | 104748 | C | NON | DNA | AGAT | 1,2 |  |
| DS-RICE3N\|DNA/hAT-Ac | | 133776 | | 134266 |  | NON | DNA | CTCAGCT | 1,2,3,4,6; truncated in 3 | |
| WANDERER_OS\|DNA/Tourist | | 138713 | | 138952 |  | NON | DNA | TA/TG | 1,2,4 |  |
| STOWAWAY12_OS\|DNA/TcMar-Stowaway | | 141399 | | 141709 |  | NON | DNA | GAAGTA | 1,2.4 |  |
| F770\|DNA/Tourist | | 145280 | | 145615 | C | NON | DNA | ATA | 1,2,3,4,6 |  |
| STOWAWAY41_OS\|DNA/TcMar-Stowaway | | 154335 | | 154581 |  | NON | DNA | TA | 1,2,3,4 |  |
| DS-RICE3N\|DNA/hAT-Ac | | 156809 | | 157278 |  | NON | DNA | GCCACATG/GCCACAGA | 1,3,4,6; truncated in 2 | |
| DITTO\|DNA/Tourist | | 157612 | | 167469 | C | NESTED | DNA | ATAA | 1,2,3,4,6 |  |
| STOWAWAY2_OS\|DNA/TcMar-Stowaway | | 168318 | | 168553 |  | NON | DNA | TA | 1,2,3,4,5,6 |  |
| STOWAWAY26_OS\|DNA/TcMar-Stowaway | | 173638 | | 173744 |  | NON | DNA | TA | 1,2,3,4,5,6 |  |
| MDM1\|DNA/MuDR | | 174362 | | 174728 |  | NON | DNA | ATCTA | 1,2,3,4,5,6 |  |
| STOWAWAY9_OS\|DNA/TcMar-Stowaway | | 174769 | | 175007 | C | NON | DNA | AT | 1,2,3,4,5,6 |  |
| CLOUD-6\|DNA/MuDR | | 180818 | | 181268 |  | NON | DNA | TTTATTATA/TTTCTTATA | 1,2,3,4,5,6 |  |
| STOWAWAY15-2_OS\|DNA/TcMar-Stowaway | | 187670 | | 187764 |  | NON | DNA | TA | 1,2,3,4,5,6 |  |
| CLOUD\|DNA/MuDR | | 189135 | | 189873 |  | NON | DNA | TAAAAAAA | 1,3,4,5,6,6 |  |
| STOWAWAY1_OS\|DNA/TcMar-Stowaway | | 191945 | | 192089 |  | NON | DNA | TA/GA | 1,2,3,4,5,6 |  |
| CASTAWAY\|DNA/Tourist | | 217057 | | 217462 |  | NON | DNA | TAA/TTC | 1,2,3,4,5,6 |  |
| CASTAWAY\|DNA/Tourist | | 217877 | | 218246 |  | NON | DNA | TAA/AAG | 1,2,3,4,5,6 |  |
| STOWAWAY2_OS\|DNA/TcMar-Stowaway | | 230927 | | 231153 |  | NON | DNA | TA | 1,2,3,4,5,6 |  |
| MUDRN1_OS\|DNA/MuDR | | 236401 | | 237422 |  | NON | DNA | AAAAAAATA/AAAAACAAA | 1,2,3,4,5,6 |  |
| STOWAWAY2_OS\|DNA/TcMar-Stowaway | | 238414 | | 238635 |  | NON | DNA | TA | 1,2,3,4,5,6 |  |
| Gaijin\|DNA/Tourist | | 251162 | | 251308 | C | NON | DNA | ATT | 1,2,3,4,5,6 |  |
| STOWAWAY1_OS\|DNA/TcMar-Stowaway | | 255169 | | 255319 | C | NON | DNA | TAA | 1,2,3,4,5,6 |  |
| STOWAWAY21_OS\|DNA/TcMar-Stowaway | | 258484 | | 258724 | C | NON | DNA | TCATA | 1,2,3,4,5,6 |  |
| ENSPM2_OS\|DNA/En-Spm | | 264306 | | 297660 |  | NESTED | DNA | TGT | 1,2,3,5,6; gap in 2 | |
| AMYLTP\|DNA/Tourist | | 298901 | | 299279 |  | NON | DNA | TAAAT/CCGAT | 1,2,3,4,5,6 |  |
| CACTA-F\|DNA/En-Spm | | 304978 | | 306080 |  | NON | DNA | AAA | 1,2,3,4,5,6 |  |
| STOWAWAY15-2_OS\|DNA/TcMar-Stowaway | | 306559 | | 306681 |  | NON | DNA | TA | 1,2,3,4,5,6 |  |
| NDNA1TNA_OS\|DNA/Tourist | | 306834 | | 307175 | C | NON | DNA | AAT/TAA | 1,2,3,4,5,6 |  |
| STOWAWAY21_OS\|DNA/TcMar-Stowaway | | 314109 | | 314344 |  | NON | DNA | TACTT/TATTT | 1,2,3,4,5,6 |  |
| SPMLIKE\|DNA/En-Spm | | 314379 | | 325692 |  | AUTO | DNA | ACAC | 1,2,4,5 |  |
| STOWAWAY25_OS\|DNA/TcMar-Stowaway | | 331432 | | 331520 |  | NON | DNA | TA | 1,2,3,4,5 |  |
| CACTA-K\|DNA/En-Spm | | 338868 | | 339953 |  | NON | DNA | TGC/TAC | 1,2,3,4,5 |  |
| WANDERER_OS\|DNA/Tourist | | 342217 | | 342457 |  | NON | DNA | TAA | 1,2,3,4,5,6 |  |
| STOWAWAY1_OS\|DNA/Stowaway | | 375805 | | 376227 | C | NON | DNA | AT | 1,2,3,4,6 |  |
| EnSpm-N2_OS\|DNA/En-Spm | | 385216 | | 386863 |  | AUTO | DNA | ATA | 1,2,3,4 |  |
| TREP215\|DNA/TcMar-Stowaway | | 402419 | | 402658 | C | NON | DNA | TG/TA | 1,2,3,4; truncated in 3 and 4 | |
| STOWAWAY44_OS\|DNA/TcMar-Stowaway | | 403723 | | 403959 |  | NON | DNA | TA | 1,2,3,4,6 |  |
| STOWAWAY15-2_OS\|DNA/TcMar-Stowaway | | 404056 | | 404174 |  | NON | DNA | TA | 1,2,3,4,6 |  |
| OSTE33\|DNA/MuDR | | 404520 | | 407455 |  | NESTED | DNA | CTATCCGCAC/CTATCCATAC | 1,2,3,4 |  |
| SEVERIN-2\|DNA\|Helitron | | 404785 | | 406721 |  | NON | DNA | ATAAA | 1,2,3,4 |  |
| SEVERIN-2\|DNA\|Helitron | | 411101 | | 413060 |  | NON | DNA | TAA | 1 |  |
| OLO24\|DNA/Tourist | | 410898 | | 413665 |  | NESTED | DNA | TGCAA/TAA | 1,2,3,6; non-coverage in 4 | |
| MERMITE18D\|DNA/MuDR | | 414049 | | 414984 | C | NON | DNA | AAAAAGA | 1,2,3,6 |  |
| STOWAWAY41_OS\|DNA/TcMar-Stowaway | | 415438 | | 415684 |  | NON | DNA | TA | 1,2,3,6 |  |
| OLO24C\|DNA/Tourist | | 416333 | | 416697 |  | NON | DNA | TAA | 1,2,3,6 |  |
| MUDRN5_OS\|DNA/MuDR | | 416907 | | 417270 | C | NON | DNA | TTTTTTTTTT | 1,2,3,6 |  |
| OLO24\|DNA/Tourist | | 417576 | | 417913 | C | NON | DNA | ATT | 1,2,3,6 |  |
| SEVERIN-2\|DNA\|Helitron | | 442096 | | 444054 |  | NON | DNA | TAA | 1,6 |  |
| MUDRN2_OS\|DNA/MuDR | | 449985 | | 450842 | C | NON | DNA | AAGCTTGTA | 1,3,6 |  |
| STOWAWAY40_OS\|DNA/TcMar-Stowaway | | 459193 | | 459431 |  | NON | DNA | TGTTT/ACAAT | 1,2,3,6 |  |
| OSTE33\|DNA/MuDR | | 479979 | | 481368 |  | NON | DNA | GCTTGCTC | 1 |  |
| CLOUD-6\|DNA/MuDR | | 483201 | | 483639 | C | NON | DNA | ATT | 1,2,3,6 |  |
| CLOUD-3\|DNA/MuDR | | 484881 | | 485397 | C | NON | DNA | AAAGATTTA | 1,2,3,6 |  |
| SEVERIN-2\|DNA\|Helitron | | 487542 | | 489980 |  | NON | DNA | AACAAAA/ATTGATTT | 1,6 |  |
| INDITTO\|DNA/Tourist | | 490775 | | 491036 | C | NON | DNA | AAT | 1,6 |  |
| F569\|DNA/Tourist | | 491040 | | 491261 |  | NON | DNA | TTA | 1,2,3,6 |  |
| NDNA1TNA_OS\|DNA/Tourist | | 491910 | | 492239 |  | NON | DNA | TGA | 1 |  |
| TYPEH\|DNA/Tourist | | 498647 | | 498957 | C | NON | DNA | ATT | 1,2,3,6 |  |
| OSTE19\|DNA/MuDR | | 511287 | | 511460 | C | NON | DNA | TAT/AAT | 1,2,3 |  |
| STOWAWAY1_OS\|DNA/TcMar-Stowaway | | 541877 | | 542023 |  | NON | DNA | TA | 1,2,3,4 |  |
| TEOS1\|DNA | | 542934 | | 543358 | C | NON | DNA | CTGAG | 1,2,3 |  |
| TNR2A\|DNA | | 547167 | | 547315 |  | NON | DNA | GA/TT | 1,2,3 |  |
| OSTE9\|DNA | | 550600 | | 550996 |  | NON | DNA | AT | 1,2,3 |  |
| ***O. sativa* L. ssp. *indica*** | |  | |  |  |  |  |  |  |  |
| ZM13-1_OS\|DNA/Tourist | | 1446 | | 1573 |  | NON | DNA | TA |  |  |
| TREP215\|DNA/TcMar-Stowaway | | 5594 | | 5815 |  | NON | DNA | TA |  |  |
| UNIQUE\|DNA/Tourist | | 16267 | | 16413 | C | NON | DNA | TAT |  |  |
| TNR2_OR\|DNA | | 17569 | | 17719 |  | NON | DNA | ACGTTATTCGG/ ACGTTATTCTT |  |  |
| STOWAWAY11_OS\|DNA/TcMar-Stowaway | | 28357 | | 28644 |  | NON | DNA | TA |  |  |
| ENSPM4_OS\|DNA/En-Spm | | 82342 | | 83690 | C | NON | DNA | AGAT |  |  |
| DS-RICE3N\|DNA/hAT-Ac | | 114096 | | 114580 |  | NON | DNA | CTCTTAGC/CTCTCAGC |  |  |
| WANDERER_OS\|DNA/Tourist | | 119104 | | 119344 |  | NON | DNA | TA/TG |  |  |
| STOWAWAY12_OS\|DNA/TcMar-Stowaway | | 121786 | | 122090 |  | NON | DNA | TA |  |  |
| F770\|DNA/Tourist | | 125662 | | 125998 | C | NON | DNA | ATA |  |  |
| STOWAWAY41_OS\|DNA/TcMar-Stowaway | | 134288 | | 134493 |  | NON | DNA | TA |  |  |
| DITTO\|DNA/Tourist | | 137297 | | 150212 | C | NESTED | DNA | ATAA |  |  |
| STOWAWAY2_OS\|DNA/TcMar-Stowaway | | 151061 | | 151299 |  | NON | DNA | TA |  |  |
| STOWAWAY26_OS\|DNA/TcMar-Stowaway | | 156989 | | 157093 |  | NON | DNA | TA |  |  |
| MDM1\|DNA/MuDR | | 157713 | | 158075 |  | NON | DNA | TA |  |  |
| STOWAWAY9_OS\|DNA/TcMar-Stowaway | | 158120 | | 158358 | C | NON | DNA | AT |  |  |
| CLOUD-6\|DNA/MuDR | | 164160 | | 164606 |  | NON | DNA | TTTATTATA/TTTCTTATA |  |  |
| STOWAWAY15-2_OS\|DNA/TcMar-Stowaway | | 171062 | | 171161 |  | NON | DNA | TA |  |  |
| STOWAWAY1_OS\|DNA/TcMar-Stowaway | | 192657 | | 192807 |  | NON | DNA | TA/GA |  |  |
| CASTAWAY\|DNA/Tourist | | 204869 | | 205274 |  | NON | DNA | TAA/TTC |  |  |
| CASTAWAY\|DNA/Tourist | | 205702 | | 206065 | C | NON | DNA | ATT | 2,3,5 |  |
| CASTAWAY\|DNA/Tourist | | 206060 | | 206398 |  | NON | DNA | AAG |  |  |
| STOWAWAY2_OS\|DNA/TcMar-Stowaway | | 219130 | | 219353 |  | NON | DNA | TA |  |  |
| MUDRN1_OS\|DNA/MuDR | | 224630 | | 225650 |  | NON | DNA | AAAAAAATA |  |  |
| STOWAWAY2_OS\|DNA/TcMar-Stowaway | | 226642 | | 226874 |  | NON | DNA | TA |  |  |
| Gaijin\|DNA/Tourist | | 237923 | | 238069 | C | NON | DNA | ATT |  |  |
| STOWAWAY1_OS\|DNA/TcMar-Stowaway | | 241928 | | 242077 | C | NON | DNA | AT |  |  |
| STOWAWAY21_OS\|DNA/TcMar-Stowaway | | 245247 | | 245485 | C | NON | DNA | AT |  |  |
| AMYLTP\|DNA/Tourist | | 281659 | | 282039 |  | NON | DNA | TAAAT/CCGAT |  |  |
| CACTA-F\|DNA/En-Spm | | 287624 | | 288793 |  | NON | DNA | AAA |  |  |
| STOWAWAY15-2_OS\|DNA/TcMar-Stowaway | | 289280 | | 289402 |  | NON | DNA | TA |  |  |
| NDNA1TNA_OS\|DNA/Tourist | | 289555 | | 289896 | C | NON | DNA | TAAGGACAGAAT/TAAGGATAGAAT |  |  |
| STOWAWAY21_OS\|DNA/TcMar-Stowaway | | 297552 | | 297782 |  | NON | DNA | TT/TA |  |  |
| SPMLIKE\|DNA/En-Spm | | 297818 | | 315497 |  | NESTED | DNA | ACACA/ACACCA |  |  |
| STOWAWAY25_OS\|DNA/TcMar-Stowaway | | 321264 | | 321370 |  | NON | DNA | TA |  |  |
| CACTA-K\|DNA/En-Spm | | 328756 | | 329846 |  | NON | DNA | TGC/TAC |  |  |
| WANDERER_OS\|DNA/Tourist | | 332111 | | 332351 |  | NON | DNA | TAA |  |  |
| STOWAWAY1_OS\|DNA/Stowaway | | 366814 | | 367236 | C | NON | DNA | AT |  |  |
| EnSpm-N2_OS\|DNA/En-Spm | | 394525 | | 396172 |  | AUTO | DNA | ATA |  |  |
| TREP215\|DNA/TcMar-Stowaway | | 393065 | | 393278 | C | NON | DNA | CTTA/GTTA |  |  |
| STOWAWAY44_OS\|DNA/TcMar-Stowaway | | 394341 | | 394578 |  | NON | DNA | CA/TA |  |  |
| STOWAWAY15-2_OS\|DNA/TcMar-Stowaway | | 394675 | | 394789 |  | NON | DNA | TA |  |  |
| OSTE33\|DNA/MuDR | | 395135 | | 398013 |  | NESTED | DNA | CTATCCGTAC/CTATCCATAC |  |  |
| SEVERIN-2\|DNA\|Helitron | | 395379 | | 397278 |  | NON | DNA | NNNNN/ATAAA |  |  |
| OLO24\|DNA/Tourist | | 401448 | | 401712 |  | NON | DNA | TGCAA/TAA |  |  |
| MERMITE18D\|DNA/MuDR | | 402100 | | 403053 | C | NON | DNA | AAAAAGA |  |  |
| STOWAWAY41_OS\|DNA/TcMar-Stowaway | | 403506 | | 403758 |  | NON | DNA | TTC/CTG |  |  |
| OLO24C\|DNA/Tourist | | 404411 | | 404755 |  | NON | DNA | TAA |  |  |
| MUDRN5_OS\|DNA/MuDR | | 404968 | | 405330 | C | NON | DNA | TCT |  |  |
| OLO24\|DNA/Tourist | | 405636 | | 405970 | C | NON | DNA | ATT |  |  |
| STOWAWAY21_OS\|DNA/TcMar-Stowaway | | 419278 | | 419513 |  | NON | DNA | TA | 2,3 |  |
| STOWAWAY40_OS\|DNA/TcMar-Stowaway | | 448883 | | 449122 |  | NON | DNA | TTT/ACA |  |  |
| CLOUD-3\|DNA/MuDR | | 457169 | | 457685 | C | NON | DNA | AAAGGTTTA/AAAGATTTA |  |  |
| F569\|DNA/Tourist | | 459721 | | 459936 |  | NON | DNA | TCTTA/CCTTA |  |  |
| TYPEH\|DNA/Tourist | | 466999 | | 467343 | C | NON | DNA | ATT |  |  |
| OSTE19\|DNA/MuDR | | 475095 | | 475255 | C | NON | DNA | TAT/AAT |  |  |
| STOWAWAY1_OS\|DNA/TcMar-Stowaway | | 508117 | | 508266 |  | NON | DNA | TA |  |  |
| TEOS1\|DNA | | 509172 | | 509606 | C | NON | DNA | CTGAG |  |  |
| TNR2A\|DNA | | 513416 | | 513561 |  | NON | DNA | GA/TT |  |  |
| OSTE9\|DNA | | 516842 | | 517346 |  | NON | DNA | AGTAT/CGTAT |  |  |
| ***O. glaberrima*** | |  | |  |  |  |  |  |  |  |
| ZM13-1_OS\|DNA/Tourist | | 730 | | 867 |  | NON | DNA | AT/TC |  |  |
| STOWAWAY2_OS\|DNA/TcMar-Stowaway | | 5662 | | 5891 |  | NON | DNA | TA | 3 |  |
| TNR2_OR\|DNA | | 16731 | | 16888 |  | NON | DNA | ATGCTATTCGG/ ACGTTATTTTT |  |  |
| STOWAWAY11_OS\|DNA/TcMar-Stowaway | | 37713 | | 38000 |  | NON | DNA | TA |  |  |
| CACTA-H\|DNA/En-Spm | | 42777 | | 43782 |  | NON | DNA | TAT |  |  |
| F770\|DNA/Tourist | | 98672 | | 98955 |  | NON | DNA | ATA |  |  |
| STOWAWAY41_OS\|DNA/TcMar-Stowaway | | 99075 | | 99333 |  | NON | DNA | TA/TT |  |  |
| TREP215\|DNA/TcMar-Stowaway | | 120870 | | 121108 |  | NON | DNA | GG/TA | 3 |  |
| CACTA-E\|DNA/En-Spm | | 131953 | | 133697 |  | NON | DNA | GAT | 3,6 |  |
| F770\|DNA/Tourist | | 142618 | | 142804 | C | NON | DNA | ATA |  |  |
| DS-RICE3N\|DNA/hAT-Ac | | 156598 | | 157070 | C | NON | DNA | GCCACATG/GCCACAGA |  |  |
| DITTO\|DNA/Tourist | | 157422 | | 174240 | C | NON | DNA | ATAA |  |  |
| STOWAWAY2_OS\|DNA/TcMar-Stowaway | | 174978 | | 175210 |  | NON | DNA | TA |  |  |
| STOWAWAY26_OS\|DNA/TcMar-Stowaway | | 180348 | | 180451 |  | NON | DNA | TA |  |  |
| MDM1\|DNA/MuDR | | 181072 | | 181434 |  | NON | DNA | TA |  |  |
| STOWAWAY9_OS\|DNA/TcMar-Stowaway | | 181479 | | 181715 | C | NON | DNA | AT |  |  |
| CLOUD-6\|DNA/MuDR | | 187506 | | 187952 |  | NON | DNA | TTTATTATA/TTTCTTATA |  |  |
| STOWAWAY15-2_OS\|DNA/TcMar-Stowaway | | 194394 | | 194493 |  | NON | DNA | TA |  |  |
| CLOUD\|DNA/MuDR | | 195833 | | 196572 |  | NON | DNA | TTAAAAAAA/TAAATAAAA |  |  |
| STOWAWAY1_OS\|DNA/TcMar-Stowaway | | 198643 | | 198793 |  | NON | DNA | TA/TGA |  |  |
| CASTAWAY\|DNA/Tourist | | 210873 | | 211279 |  | NON | DNA | TAA/TTC |  |  |
| CASTAWAY\|DNA/Tourist | | 211694 | | 212063 |  | NON | DNA | CTA/AAA |  |  |
| STOWAWAY2_OS\|DNA/TcMar-Stowaway | | 224758 | | 224988 |  | NON | DNA | TA |  |  |
| MUDRN1_OS\|DNA/MuDR | | 253829 | | 254850 |  | NON | DNA | AAAATA/AAAAAA |  |  |
| STOWAWAY2_OS\|DNA/TcMar-Stowaway | | 255822 | | 256056 |  | NON | DNA | TA |  |  |
| Gaijin\|DNA/Tourist | | 272293 | | 272439 | C | NON | DNA | ATT |  |  |
| STOWAWAY1_OS\|DNA/TcMar-Stowaway | | 276251 | | 276400 | C | NON | DNA | ATT |  |  |
| STOWAWAY21_OS\|DNA/TcMar-Stowaway | | 279564 | | 279801 | C | NON | DNA | AT |  |  |
| ENSPM2_OS\|DNA/En-Spm | | 285418 | | 297514 |  | NESTED | DNA | TGT |  |  |
| AMYLTP\|DNA/Tourist | | 298755 | | 299135 |  | NON | DNA | TAAAT/CCGAT |  |  |
| CACTA-F\|DNA/En-Spm | | 304950 | | 306006 |  | NON | DNA | AAA |  |  |
| STOWAWAY15-2_OS\|DNA/TcMar-Stowaway | | 306484 | | 306605 |  | NON | DNA | TA |  |  |
| NDNA1TNA_OS\|DNA/Tourist | | 306758 | | 307099 | C | NON | DNA | TAAGGACAGAAT |  |  |
| STOWAWAY21_OS\|DNA/TcMar-Stowaway | | 314549 | | 314784 |  | NON | DNA | TT/TA |  |  |
| STOWAWAY25_OS\|DNA/TcMar-Stowaway | | 320612 | | 320718 |  | NON | DNA | TA |  |  |
| CACTA-K\|DNA/En-Spm | | 328093 | | 329203 |  | NON | DNA | TGC/TAC |  |  |
| WANDERER_OS\|DNA/Tourist | | 331480 | | 331722 |  | NON | DNA | TAA |  |  |
| STOWAWAY1_OS\|DNA/Stowaway | | 365068 | | 365529 | C | NON | DNA | AT |  |  |
| EnSpm-N2_OS\|DNA/En-Spm | | 384038 | | 385663 |  | NON | DNA | ATA |  |  |
| STOWAWAY44_OS\|DNA/TcMar-Stowaway | | 393083 | | 393320 |  | NON | DNA | TAAGACA/TAA |  |  |
| STOWAWAY15-2_OS\|DNA/TcMar-Stowaway | | 393417 | | 393535 |  | NON | DNA | TA |  |  |
| OSTE33\|DNA/MuDR | | 393881 | | 396800 |  | NESTED | DNA | CTATCAGTAC/CTATCCATAC |  |  |
| SEVERIN-2\|DNA/helitron | | 394137 | | 396612 |  | NON | DNA |  |  |  |
| OLO24\|DNA/Tourist | | 400207 | | 400470 |  | NON | DNA | TGCAA/TAA |  |  |
| MERMITE18D\|DNA/MuDR | | 400858 | | 401804 | C | NON | DNA | AAAAAGA |  |  |
| STOWAWAY41_OS\|DNA/TcMar-Stowaway | | 402256 | | 402507 |  | NON | DNA | TTC/CTG |  |  |
| OLO24C\|DNA/Tourist | | 403164 | | 403508 |  | NON | DNA | TAA |  |  |
| MUDRN5_OS\|DNA/MuDR | | 403719 | | 404094 | C | NON | DNA | TTTTTTTTTT |  |  |
| OLO24\|DNA/Tourist | | 404398 | | 404734 | C | NON | DNA | ATT |  |  |
| STOWAWAY21_OS\|DNA/TcMar-Stowaway | | 418058 | | 418292 |  | NON | DNA | TA |  |  |
| MUDRN2_OS\|DNA/MuDR | | 434171 | | 435300 | C | NON | DNA | AAGCTTGTA/AATCTTGTA |  |  |
| AMYLTP\|DNA/Tourist | | 438769 | | 439552 |  | NON | DNA | CTATTGACT | 3 |  |
| STOWAWAY40_OS\|DNA/TcMar-Stowaway | | 441879 | | 442094 |  | NON | DNA | TTT/ACA |  |  |
| CLOUD-3\|DNA/MuDR | | 452021 | | 452536 | C | NON | DNA | AAAGATTTA |  |  |
| F569\|DNA/Tourist | | 454564 | | 454780 |  | NON | DNA | TTA |  |  |
| TYPEH\|DNA/Tourist | | 461064 | | 461403 | C | NON | DNA | AT |  |  |
| OSTE19\|DNA/MuDR | | 473486 | | 473649 | C | NON | DNA | AAT |  |  |
| STOWAWAY1_OS\|DNA/TcMar-Stowaway | | 497268 | | 497409 |  | NON | DNA | TA |  |  |
| TEOS1\|DNA | | 498320 | | 498739 | C | NON | DNA | CTGAG |  |  |
| TNR2A\|DNA | | 502585 | | 502730 |  | NON | DNA | GA/TT |  |  |
| OSTE9\|DNA | | 506010 | | 506409 |  | NON | DNA | AGTAT |  |  |
| Gaijin\|DNA/Tourist | | 513870 | | 514016 | C | NON | DNA | GAT/ATT |  |  |
| ***O. rufipogon*** | |  | |  |  |  |  |  |  |  |
| DS-RICE3N\|DNA/hAT-Ac | | 17477 | | 17960 |  | NON | DNA | CTCTCAGC |  |  |
| WANDERER_OS\|DNA/Tourist | | 22434 | | 22673 |  | NON | DNA | TA/TG |  |  |
| STOWAWAY12_OS\|DNA/TcMar-Stowaway | | 25117 | | 25420 |  | NON | DNA | TA |  |  |
| F770\|DNA/Tourist | | 28990 | | 29326 | C | NON | DNA | ATA |  |  |
| STOWAWAY41_OS\|DNA/TcMar-Stowaway | | 37740 | | 37986 |  | NON | DNA | TA/TT |  |  |
| DS-RICE3N\|DNA/hAT-Ac | | 40224 | | 40677 |  | NON | DNA | GCCACATG/GCCACAGA |  |  |
| DITTO\|DNA/Tourist | | 41010 | | 53897 | C | NON | DNA | ATAA |  |  |
| STOWAWAY2_OS\|DNA/TcMar-Stowaway | | 54748 | | 54986 |  | NON | DNA | TA |  |  |
| STOWAWAY26_OS\|DNA/TcMar-Stowaway | | 60114 | | 60220 |  | NON | DNA | TA |  |  |
| MDM1\|DNA/MuDR | | 60838 | | 61200 |  | NON | DNA | TA |  |  |
| STOWAWAY9_OS\|DNA/TcMar-Stowaway | | 61245 | | 61479 | C | NON | DNA | AT |  |  |
| CLOUD-6\|DNA/MuDR | | 79265 | | 79710 |  | NON | DNA | TTTATTATA/TTTCTTATA |  |  |
| STOWAWAY15-2_OS\|DNA/TcMar-Stowaway | | 86163 | | 86262 |  | NON | DNA | TA |  |  |
| CLOUD\|DNA/MuDR | | 87628 | | 88367 |  | NON | DNA | TAAAAAAA |  |  |
| STOWAWAY1_OS\|DNA/TcMar-Stowaway | | 90438 | | 90590 |  | NON | DNA | TA/GA |  |  |
| CASTAWAY\|DNA/Tourist | | 102648 | | 103053 |  | NON | DNA | TAA/TTC |  |  |
| CASTAWAY\|DNA/Tourist | | 103469 | | 103832 | C | NON | DNA | ATT |  |  |
| CASTAWAY\|DNA/Tourist | | 103827 | | 104164 |  | NON | DNA | AAG |  |  |
| STOWAWAY2_OS\|DNA/TcMar-Stowaway | | 117416 | | 117638 |  | NON | DNA | TA |  |  |
| MUDRN1_OS\|DNA/MuDR | | 122899 | | 123915 |  | NON | DNA | AAAAAAATA/AAAAAACAA |  |  |
| STOWAWAY2_OS\|DNA/TcMar-Stowaway | | 124904 | | 125123 |  | NON | DNA | TA |  |  |
| Gaijin\|DNA/Tourist | | 136196 | | 136343 | C | NON | DNA | TT |  |  |
| STOWAWAY1_OS\|DNA/TcMar-Stowaway | | 140200 | | 140349 | C | NON | DNA | AT |  |  |
| STOWAWAY21_OS\|DNA/TcMar-Stowaway | | 143518 | | 143756 | C | NON | DNA | AT |  |  |
| AMYLTP\|DNA/Tourist | | 210406 | | 210785 |  | NON | DNA | AAT/CCG |  |  |
| CACTA-F\|DNA/En-Spm | | 216341 | | 217482 |  | NON | DNA | AAA |  |  |
| STOWAWAY15-2_OS\|DNA/TcMar-Stowaway | | 217969 | | 218097 |  | NON | DNA | TA |  |  |
| NDNA1TNA_OS\|DNA/Tourist | | 218250 | | 218593 | C | NON | DNA | AGAAT/AGGAT |  |  |
| STOWAWAY21_OS\|DNA/TcMar-Stowaway | | 223707 | | 223935 |  | NON | DNA | TT/TA |  |  |
| SPMLIKE\|DNA/En-Spm | | 223971 | | 234738 |  | AUTO | DNA | ACA |  |  |
| STOWAWAY25_OS\|DNA/TcMar-Stowaway | | 240505 | | 240611 |  | NON | DNA | TA |  |  |
| CACTA-K\|DNA/En-Spm | | 248064 | | 249144 |  | NON | DNA | TAC |  |  |
| WANDERER_OS\|DNA/Tourist | | 266169 | | 266409 |  | NON | DNA | TAA |  |  |
| STOWAWAY1_OS\|DNA/Stowaway | | 332555 | | 332977 | C | NON | DNA | AT |  |  |
| EnSpm-N2_OS\|DNA/En-Spm | | 366002 | | 367649 |  | NON | DNA | ATA |  |  |
| STOWAWAY44_OS\|DNA/TcMar-Stowaway | | 388255 | | 388479 |  | NON | DNA | TA |  |  |
| STOWAWAY15-2_OS\|DNA/TcMar-Stowaway | | 388576 | | 388694 |  | NON | DNA | TA |  |  |
| OSTE33\|DNA/MuDR | | 389040 | | 391921 |  | NESTED | DNA | CTATCCGTAC/CTATCCATAC |  |  |
| SEVERIN-2\|DNA/helitron | | 389297 | | 391234 |  | NON | DNA | AAA/TTG |  |  |
| ***O. nivara*** | |  | |  |  |  |  |  |  |  |
| STOWAWAY2_OS\|DNA/TcMar-Stowaway | | 18920 | | 19158 |  | NON | DNA | TA |  |  |
| STOWAWAY26_OS\|DNA/TcMar-Stowaway | | 24285 | | 24391 |  | NON | DNA | TA |  |  |
| MDM1\|DNA/MuDR | | 25009 | | 25375 |  | NON | DNA | ATCTA |  |  |
| STOWAWAY9_OS\|DNA/TcMar-Stowaway | | 25416 | | 25650 | C | NON | DNA | TA |  |  |
| SETARIA2\|DNA/MuDR | | 32767 | | 36987 | C | NESTED | DNA | TAATT | 5 |  |
| CLOUD-6\|DNA/MuDR | | 37810 | | 38255 |  | NON | DNA | TTTATTATA/TTTCTTATA |  |  |
| STOWAWAY15-2_OS\|DNA/TcMar-Stowaway | | 44706 | | 44805 |  | NON | DNA | TA |  |  |
| CLOUD\|DNA/MuDR | | 46169 | | 46907 |  | NON | DNA | TAAAAAAA |  |  |
| STOWAWAY1_OS\|DNA/TcMar-Stowaway | | 48978 | | 49128 |  | NON | DNA | TA/GA |  |  |
| CASTAWAY\|DNA/Tourist | | 61184 | | 61589 |  | NON | DNA | TAA/TTC |  |  |
| CASTAWAY\|DNA/Tourist | | 62005 | | 62368 | C | NON | DNA | TTA |  |  |
| CASTAWAY\|DNA/Tourist | | 62363 | | 62700 |  | NON | DNA | AAG |  |  |
| STOWAWAY2_OS\|DNA/TcMar-Stowaway | | 75434 | | 75653 |  | NON | DNA | TA |  |  |
| MUDRN1_OS\|DNA/MuDR | | 80914 | | 81933 |  | NON | DNA | AAAAAAATA/AAAAACAAA |  |  |
| STOWAWAY2_OS\|DNA/TcMar-Stowaway | | 82921 | | 83140 |  | NON | DNA | TA |  |  |
| Gaijin\|DNA/Tourist | | 94195 | | 94341 | C | NON | DNA | TTA |  |  |
| STOWAWAY1_OS\|DNA/TcMar-Stowaway | | 98197 | | 98346 | C | NON | DNA | TA |  |  |
| STOWAWAY21_OS\|DNA/TcMar-Stowaway | | 101515 | | 101753 | C | NON | DNA | TA |  |  |
| ENSPM2_OS\|DNA/En-Spm | | 107279 | | 163349 |  | NESTED | DNA | TGC/TGT |  |  |
| AMYLTP\|DNA/Tourist | | 164589 | | 164968 |  | NON | DNA | TAAAT/CCGAT |  |  |
| CACTA-F\|\|DNA/En-Spm | | 170671 | | 171814 |  | NON | DNA | AAA |  |  |
| STOWAWAY15-2_OS\|DNA/TcMar-Stowaway | | 172300 | | 172429 |  | NON | DNA | TA |  |  |
| NDNA1TNA_OS\|DNA/Tourist | | 172582 | | 172923 | C | NON | DNA | TAA/AAT |  |  |
| STOWAWAY21_OS\|DNA/TcMar-Stowaway | | 178039 | | 178267 |  | NON | DNA | TACTT/TATTT |  |  |
| SPMLIKE\|DNA/En-Spm | | 178303 | | 188681 |  | AUTO | DNA | ACA |  |  |
| STOWAWAY25_OS\|DNA/TcMar-Stowaway | | 194448 | | 194554 |  | NON | DNA | TA |  |  |
| CACTA-K\|DNA/En-Spm | | 202011 | | 202994 |  | NON | DNA | TAC |  |  |
| WANDERER_OS\|DNA/Tourist | | 226966 | | 227206 |  | NON | DNA | TAA |  |  |
| ***O. glumaepatula*** | |  | |  |  |  |  |  |  |  |
| DS-RICE3N\|DNA/hAT-Ac | | 13910 | | 14391 |  | NON | DNA | CTCTCAGCT |  |  |
| CACTA-E\|DNA/En-Spm | | 15399 | | 17191 |  | NON | DNA | GAT |  |  |
| AMYLTP\|DNA/Tourist | | 22396 | | 22779 | C | NON | DNA | CTATTTCAA | 6 |  |
| F770\|DNA/Tourist | | 26502 | | 26688 | C | NON | DNA | ATA |  |  |
| DS-RICE3N\|DNA/hAT-Ac | | 40493 | | 40962 |  | NON | DNA | GCCACATG/GCCACAGA |  |  |
| DITTO\|DNA/Tourist | | 41308 | | 51159 | C | NESTED | DNA | ATAA |  |  |
| STOWAWAY2_OS\|DNA/TcMar-Stowaway | | 52017 | | 52250 |  | NON | DNA | TA |  |  |
| STOWAWAY26_OS\|DNA/TcMar-Stowaway | | 57376 | | 57482 |  | NON | DNA | TA |  |  |
| MDM1\|DNA/MuDR | | 58100 | | 58462 |  | NON | DNA | TA |  |  |
| STOWAWAY9_OS\|DNA/TcMar-Stowaway | | 58507 | | 58745 | C | NON | DNA | TA |  |  |
| CLOUD-6\|DNA/MuDR | | 64349 | | 64786 |  | NON | DNA | TTTATTATA/TTTCTTATA |  |  |
| STOWAWAY15-2_OS\|DNA/TcMar-Stowaway | | 89120 | | 89221 |  | NON | DNA | TA/TT |  |  |
| CLOUD\|DNA/MuDR | | 90580 | | 91317 |  | NON | DNA | TAAAAAAA |  |  |
| STOWAWAY1_OS\|DNA/TcMar-Stowaway | | 93391 | | 93542 |  | NON | DNA | TA/TG |  |  |
| CASTAWAY\|DNA/Tourist | | 105572 | | 105978 |  | NON | DNA | TAA/TTC |  |  |
| CASTAWAY\|DNA/Tourist | | 106394 | | 106762 |  | NON | DNA | TAA/AAG |  |  |
| STOWAWAY2_OS\|DNA/TcMar-Stowaway | | 119448 | | 119688 |  | NON | DNA | TA |  |  |
| MUDRN1_OS\|DNA/MuDR | | 136575 | | 137597 |  | NON | DNA | AAAAAAATA/AAAAACAAA |  |  |
| STOWAWAY2_OS\|DNA/TcMar-Stowaway | | 138588 | | 138821 |  | NON | DNA | TA |  |  |
| Gaijin\|DNA/Tourist | | 154194 | | 154340 | C | NON | DNA | TTA |  |  |
| STOWAWAY1_OS\|DNA/TcMar-Stowaway | | 158200 | | 158349 | C | NON | DNA | TA |  |  |
| STOWAWAY21_OS\|DNA/TcMar-Stowaway | | 161518 | | 161755 | C | NON | DNA | TA |  |  |
| ENSPM2_OS\|DNA/En-Spm | | 167321 | | 205201 |  | NESTED | DNA | TGT |  |  |
| AMYLTP\|DNA/Tourist | | 206442 | | 206821 |  | NON | DNA | AAT/CCG |  |  |
| CACTA-F\|DNA | | 212448 | | 213593 |  | NON | DNA | AAA |  |  |
| STOWAWAY15-2_OS\|DNA/TcMar-Stowaway | | 214072 | | 214194 |  | NON | DNA | TA |  |  |
| NDNA1TNA_OS\|DNA/Tourist | | 214347 | | 214689 | C | NON | DNA | AA/TA |  |  |
| STOWAWAY21_OS\|DNA/TcMar-Stowaway | | 222292 | | 222528 |  | NON | DNA | TT/TA |  |  |
| COWARD-3\|DNA/Tourist | | 247036 | | 247238 |  | NON | DNA | TAC/TAA | 6 |  |
| WANDERER_OS\|DNA/Tourist | | 251692 | | 251914 |  | NON | DNA | TAA |  |  |
| STOWAWAY1_OS\|DNA/TcMar-Stowaway | | 301038 | | 301171 | C | NON | DNA | TA |  |  |
| CLOUD\|DNA/MuDR | | 301241 | | 301983 |  | NON | DNA | TTTTTTAAA | 6 |  |
| TESS\|DNA/hAT | | 318337 | | 318579 | C | NON | DNA | CTTGC | 6 |  |
| STOWAWAY21_OS\|DNA/TcMar-Stowaway | | 319840 | | 320060 | C | NON | DNA | TA | 6 |  |
| STOWAWAY44_OS\|DNA/TcMar-Stowaway | | 340427 | | 340663 |  | NON | DNA | TA/CA |  |  |
| STOWAWAY15-2_OS\|DNA/TcMar-Stowaway | | 340731 | | 340848 |  | NON | DNA | CG/TA |  |  |
| YOUREN\|DNA/Tourist | | 340996 | | 341282 | C | NON | DNA | TTA | 6 |  |
| OLO24\|DNA/Tourist | | 359705 | | 359968 |  | NON | DNA | CAA/TAA |  |  |
| MERMITE18D\|DNA/MuDR | | 360355 | | 361303 | C | NON | DNA | AAAAAGA |  |  |
| STOWAWAY41_OS\|DNA/TcMar-Stowaway | | 361799 | | 362055 |  | NON | DNA | TA/TT |  |  |
| OLO24C\|DNA/Tourist | | 362671 | | 363034 |  | NON | DNA | TAA |  |  |
| MUDRN5_OS\|DNA/MuDR | | 363247 | | 363646 | C | NON | DNA | TATTTTTTT/TCTTTTTTT |  |  |
| OLO24\|DNA/Tourist | | 363952 | | 364286 | C | NON | DNA | TTA |  |  |
| SEVERIN-2\|RC/Helitron | | 384821 | | 386774 |  | NON | DNA | TAA |  |  |
| TNR12\|DNA/MuDR | | 390411 | | 396133 |  | NON | DNA | TTTTTTCTT/TTTTTCTT | 6 |  |
| MUDRN1_OS\|DNA/MuDR | | 398377 | | 399237 | C | NON | DNA | AAGCTTGTA |  |  |
| STOWAWAY40_OS\|DNA/TcMar-Stowaway | | 407571 | | 407807 |  | NON | DNA | TGTTT/ACAAT |  |  |
| CLOUD-6\|DNA/MuDR | | 420325 | | 420763 | C | NON | DNA | TTAGCATTA/TTACCATTA |  |  |
| CLOUD-3\|DNA/MuDR | | 422005 | | 422523 | C | NON | DNA | ATTTAGAAA |  |  |
| SEVERIN-2\|DNA\|Helitron | | 424358 | | 426967 |  | NON | DNA | AACAAAA/ATTGATTT |  |  |
| INDITTO\|DNA/Tourist | | 427742 | | 428004 | C | NON | DNA | TAA |  |  |
| F569\|DNA/Tourist | | 428008 | | 428225 |  | NON | DNA | TTA |  |  |
| TYPEH\|DNA/Tourist | | 435144 | | 435487 | C | NON | DNA | TTA |  |  |
| ***O. punctata*** | |  | |  |  |  |  |  |  |  |
| CASTAWAY\|DNA/Tourist | | 30569 | | 30926 | C | NON | DNA | AAT/ATT |  |  |
| ID-3\|DNA/Tourist | | 55147 | | 55426 |  | NON | DNA | TAA |  |  |
| ID-3\|DNA/Tourist | | 55430 | | 55710 |  | NON | DNA | TAA |  |  |
| OSTE17\|DNA/MuDR | | 59838 | | 60522 | C | NON | DNA | TATTT/AATTA |  |  |
| ZhAT2_ZM\|DNA/hAT-Tip100 | | 62730 | | 63544 |  | NON | DNA | CAACCAAA |  |  |
| ENSPM4_OS\|DNA/En-Spm | | 64211 | | 73633 |  | AUTO | DNA | ATT |  |  |
| DITAILA\|DNA/Tourist | | 88656 | | 88913 | C | NON | DNA | CAA |  |  |
| STOWAWAY48_OS\|DNA/TcMar-Stowaway | | 94714 | | 94974 | C | NON | DNA | GA/GC |  |  |
| STOWAWAY41_OS\|DNA/TcMar-Stowaway | | 96985 | | 97222 |  | NON | DNA | TA |  |  |
| OLO24C\|DNA/Tourist | | 100686 | | 101021 | C | NON | DNA | AGTC |  |  |
| ENSPM7_OS\|DNA/En-Spm | | 419813 | | 426477 |  | AUTO | DNA | TGA |  |  |
| DITTO\|DNA/Tourist | | 467852 | | 468084 |  | NON | DNA | AAT/AAA |  |  |
| MuDR3_OS\|DNA/MuDR | | 553925 | | 565461 |  | NESTED | DNA | CAAGAAAAA |  |  |
| DITAILA\|DNA/Tourist | | 599680 | | 599926 |  | NON | DNA | TAC |  |  |
| ***O. officinalis*** | |  | |  |  |  |  |  |  |  |
| STOWAWAY41_OS\|DNA/TcMar-Stowaway | | 35198 | | 35463 | C | NON | DNA | TA/AC |  |  |
| ENSPM2_OS\|DNA/En-Spm | | 38077 | | 46609 |  | AUTO | DNA | AAG |  |  |
| ENSPM5_OS\|DNA/En-Spm | | 179048 | | 188641 | C | AUTO | DNA | CTT |  |  |
| STOWAWAY2_OS\|DNA/TcMar-Stowaway | | 214618 | | 214849 | C | NON | DNA | TA |  |  |
| HARB-N1_OS\|DNA/Harbinger | | 263206 | | 264075 |  | NON | DNA | TAA |  |  |
| STOWAWAY13_OS\|DNA/TcMar-Stowaway | | 264615 | | 264854 |  | NON | DNA | TA |  |  |
| ENSPM2_OS\|DNA/En-Spm | | 265678 | | 276021 | C | AUTO | DNA | TGA |  |  |
| MUDRN1_OS\|DNA/MuDR | | 345218 | | 345986 | C | NON | DNA | AAATAAATT |  |  |
| CACTA-I\|DNA/En-Spm | | 346265 | | 346828 | C | NON | DNA | TAT |  |  |
| ENSPM2_OS\|DNA/En-Spm | | 356362 | | 362653 | C | NON | DNA | TGT |  |  |
| SUSU\|DNA/Tourist | | 368951 | | 369239 | C | NON | DNA | AAT |  |  |
| SPMLIKE\|DNA/En-Spm | | 370011 | | 380564 |  | AUTO | DNA | ACT |  |  |
| WANDERER_OS\|DNA/Tourist | | 382104 | | 382342 | C | NON | DNA | AT |  |  |
| OSTE20\|DNA/MuDR | | 382387 | | 382873 | C | NON | DNA | TATATAG |  |  |
| ***O. australiensis*** | |  | |  |  |  |  |  |  |  |
| STOWAWAY48_OS\|DNA/TcMar-Stowaway | | 52616 | | 52878 |  | NON | DNA | TG |  |  |
| DITTO3\|DNA/Tourist | | 136242 | | 137776 |  | NON | DNA | TAA |  |  |
| STOWAWAY34_OS\|DNA/TcMar-Stowaway | | 163093 | | 163323 |  | NON | DNA | TA |  |  |
| CACTA-P\|DNAEn-Spm | | 273782 | | 274923 |  | NON | DNA | CCC |  |  |
| SPMLIKE\|DNA/En-Spm | | 395098 | | 413106 |  | NESTED | DNA | ATT |  |  |
| CACTA-P\|DNAEn-Spm | | 413962 | | 414891 |  | NON | DNA | TGT |  |  |
| STOWAWAY10_OS\|DNA/TcMar-Stowaway | | 416571 | | 416810 | C | NON | DNA | TA |  |  |
| OSTE23\|DNA | | 419559 | | 419712 |  | NON | DNA | TTGAGAT/TTGTGAT |  |  |
| STOWAWAY10_OS\|DNA/TcMar-Stowaway | | 419802 | | 420063 |  | NON | DNA | AA/TA |  |  |
| ENSPM6_OS\|DNA/En-Spm | | 421206 | | 428470 |  | AUTO | DNA | ATA |  |  |
| OSHOOTER\|DNA/En-Spm | | 610372 | | 615301 |  | AUTO | DNA | AGA |  |  |
| TWIFBIG\|DNA/hAT-Ac | | 675936 | | 679284 |  | NON | DNA | GAAGTGCGG |  |  |
| SPMLIKE\|DNA/En-Spm | | 680654 | | 691623 |  | AUTO | DNA | GTT |  |  |
| ***O. brachyantha*** | |  | |  |  |  |  |  |  |  |
| DITTO\|DNA/Tourist | | 6985 | | 7213 | C | NON | DNA | AT/AAT |  |  |
| MDM2\|DNA/MuDR | | 38587 | | 38901 |  | NON | DNA | ATGTTGTAA/ATTTTGTAA |  |  |
| TREP215\|DNA/TcMar-Stowaway | | 74008 | | 74252 |  | NON | DNA | TA |  |  |
| TYPEU4\|DNA | | 75539 | | 75899 |  | NON | DNA | TG |  |  |
| OSTE1\|DNA/MuDR | | 88538 | | 89811 |  | NON | DNA | AAGAA |  |  |
| STOWAWAY1_OS\|DNA/TcMar-Stowaway | | 129099 | | 129249 |  | NON | DNA | TA |  |  |
| DITTO\|DNA/Tourist | | 136219 | | 136422 | C | NON | DNA | AAT |  |  |
| STOWAWAY1_OS\|DNA/TcMar-Stowaway | | 173517 | | 173664 | C | NON | DNA | TA |  |  |
| TREP215\|DNA/TcMar-Stowaway | | 187594 | | 187810 | C | NON | DNA | CGTTC/AATTT |  |  |
| OLO24\|DNA/Tourist | | 189298 | | 189641 | C | NON | DNA | AAT |  |  |
| DITTO-2\|DNA/Tourist | | 232947 | | 233174 | C | NON | DNA | ATT |  |  |
| CLOUD-6\|DNA/MuDR | | 248771 | | 249193 |  | NON | DNA | TTTTAAAAA |  |  |
| STOWAWAY50_OS\|DNA/TcMar-Stowaway | | 250394 | | 250546 | C | NON | DNA | TA |  |  |
| STOWAWAY51_OS\|DNA/TcMar-Stowaway | | 254903 | | 255147 |  | NON | DNA | AT |  |  |
| TEMPINDAS\|DNA/hAT-Ac | | 271635 | | 272096 | C | NON | DNA | CGATGTTG |  |  |
| YOUREN\|DNA/Tourist | | 274939 | | 275224 | C | NON | DNA | ATT |  |  |
| OSTE20\|DNA/MuDR | | 276527 | | 277474 | C | NON | DNA | AAA |  |  |
| ***S. bicolor*** | |  | |  |  |  |  |  |  |  |
| STOWAWAY2_ZM\|DNA/TcMar-Stowaway | | 1182 | | 1437 |  | NON | DNA | TA |  |  |
| hAT-18_ZM\|DNA/hAT-Tag1 | | 47664 | | 48233 |  | NON | DNA | GCTCTCACTCC/GGTCTCACTTT |  |  |
| ENSPM-6_ZM\|DNA/En-Spm | | 57665 | | 62284 | C | NON | DNA | TTC |  |  |
| ENSPM-N1_ZM\|DNA/En-Spm | | 62687 | | 63476 |  | NON | DNA | AGT |  |  |
| STOWAWAY41_OS\|DNA/TcMar-Stowaway | | 69255 | | 69506 |  | NON | DNA | TA |  |  |
| STOWAWAY2_OS\|DNA/TcMar-Stowaway | | 71589 | | 71844 |  | NON | DNA | TA |  |  |
| EnSpm-10_ZM\|DNA/En-Spm | | 98061 | | 101474 | C | NON | DNA | ATT |  |  |
| ENSPM-6_ZM\|DNA/En-Spm | | 123380 | | 130074 | C | AUTO | DNA | ACA |  |  |
| STOWAWAY17_OS\|DNA/TcMar-Stowaway | | 137490 | | 137720 |  | NON | DNA | TA |  |  |
| OLO24\|DNA/Tourist | | 299560 | | 299902 | C | NON | DNA | GGAAT |  |  |
| ENSPM-6_ZM\|DNA/En-Spm | | 309986 | | 310936 |  | NON | DNA | AAA |  |  |
| ENSPM-6_ZM\|DNA/En-Spm | | 396650 | | 397812 | C | NON | DNA | ACA |  |  |
| ENSPM-7_ZM\|DNA/En-Spm | | 398572 | | 411544 | C | AUTO | DNA | TTT |  |  |
| STOWAWAY2_ZM\|DNA/TcMar-Stowaway | | 527316 | | 527565 |  | NON | DNA | TA |  |  |
| EnSpm-10_ZM\|DNA/En-Spm | | 602060 | | 621338 |  | AUTO | DNA | TATGCA |  |  |
| DITTO-2\|DNA/Tourist | | 639941 | | 640181 | C | NON | DNA | ACT |  |  |
| EnSpm-12_ZM\|DNA/En-Spm | | 662763 | | 667748 | C | AUTO | DNA | AGA |  |  |
| STOWAWAY2_ZM\|DNA/TcMar-Stowaway | | 680308 | | 680542 |  | NON | DNA | TA |  |  |
| ENSPM-6_ZM\|DNA/En-Spm | | 824692 | | 825713 | C | NON | DNA | TTA |  |  |
| STOWAWAY2_ZM\|DNA/TcMar-Stowaway | | 831199 | | 831452 | C | NON | DNA | TA |  |  |
| HAZEAN1\|DNA/hAT | | 872600 | | 873324 | C | NON | DNA | CGATCCTA |  |  |
| SPMLIKE\|DNA/En-Spm | | 888619 | | 894193 |  | AUTO | DNA | ACA |  |  |
| DITTO-2\|DNA/Tourist | | 902996 | | 903236 | C | NON | DNA | ATT |  |  |
| OLO24\|DNA/Tourist | | 903752 | | 904085 | C | NON | DNA | TAA |  |  |
| OLO24\|DNA/Tourist | | 912624 | | 912969 |  | NON | DNA | TTA |  |  |
| ENSPM-6_ZM\|DNA/En-Spm | | 943902 | | 945441 | C | AUTO | DNA | ATG |  |  |
| EnSpm-10_ZM\|DNA/En-Spm | | 986375 | | 990175 |  | AUTO | DNA | ATA |  |  |
| EnSpm-10_ZM\|DNA/En-Spm | | 1079769 | | 1084587 | C | AUTO | DNA | GAC |  |  |
| EnSpm-10_ZM\|DNA/En-Spm | | 1099370 | | 1120992 | C | NESTED | DNA | TTCT |  |  |
| ENSPM-6_ZM\|DNA/En-Spm | | 1153726 | | 1159035 | C | AUTO | DNA | AAT |  |  |
| STOWAWAY17_OS\|DNA/TcMar-Stowaway | | 1168013 | | 1168272 |  | NON | DNA | TA |  |  |
| STOWAWAY41_OS\|DNA/TcMar-Stowaway | | 1169599 | | 1169864 |  | NON | DNA | TA |  |  |
| EnSpm-10_ZM\|DNA/En-Spm | | 1184750 | | 1200956 | C | AUTO | DNA | TCC |  |  |
| QIQI\|DNA/Tourist | | 1203575 | | 1203894 | C | NON | DNA | TGA |  |  |
| STOWAWAY2_ZM\|DNA/TcMar-Stowaway | | 1205840 | | 1206096 | C | NON | DNA | AT |  |  |
| OLO24C\|DNA/Tourist | | 1209474 | | 1209725 | C | NON | DNA | TAA |  |  |
| STOWAWAY40_OS\|DNA/TcMar-Stowaway | | 1221003 | | 1221238 |  | NON | DNA | TA |  |  |
| STOWAWAY17_OS\|DNA/TcMar-Stowaway | | 1246295 | | 1246550 | C | NON | DNA | TA |  |  |
| ENSPM-N1_ZM\|DNA/En-Spm | | 1251474 | | 1251572 | C | NON | DNA | TAT |  |  |
| ENSPM-6_ZM\|DNA/En-Spm | | 1264116 | | 1264747 |  | NON | DNA | GAA |  |  |
| ENSPM-6_ZM\|DNA/En-Spm | | 1298989 | | 1302166 | C | AUTO | DNA | CAC |  |  |
| STOWAWAY17_OS\|DNA/TcMar-Stowaway | | 1303113 | | 1303366 | C | NON | DNA | TA |  |  |
| ENSPM-6_ZM\|DNA/En-Spm | | 1330201 | | 1334649 |  | AUTO | DNA | CAA |  |  |
| ENSPM1_ZM\|DNA/En-Spm | | 1643768 | | 1651045 | C | AUTO | DNA | ATT |  |  |
| OLO24\|DNA/Tourist | | 1701973 | | 1702321 | C | NON | DNA | TAA |  |  |
| EnSpm-10_ZM\|DNA/En-Spm | | 1714271 | | 1717858 |  | AUTO | DNA | TAG |  |  |
| STOWAWAY44_OS\|DNA/TcMar-Stowaway | | 1727961 | | 1728190 | C | NON | DNA | TA |  |  |
| STOWAWAY41_OS\|DNA/TcMar-Stowaway | | 1829934 | | 1830194 |  | NON | DNA | TA |  |  |
| OLO24\|DNA/Tourist | | 1857677 | | 1858036 | C | NON | DNA | TTA |  |  |
| ENSPM-6_ZM\|DNA/En-Spm | | 2091714 | | 2092726 |  | NON | DNA | AGA |  |  |
| ***Z. mays*** | |  | |  |  |  |  |  |  |  |
| CLOUD\|DNA/MuDR | | 6229 | | 6524 | C | NON | DNA | AACA |  |  |
| HAZEMAN1\|DNA | | 174207 | | 174682 | C | NON | DNA | TGA |  |  |
| ZhATN1_ZM\|DNA/hAT-Ac | | 302702 | | 303818 | C | NON | DNA | CAAGGCCG |  |  |
| ZhAT13_ZM\|DNA/hAT-Tag1 | | 387125 | | 388089 |  | NON | DNA | ATGTA |  |  |
| HARB3_ZM\|DNA/Harbinger | | 390125 | | 391130 |  | NON | DNA | TAA |  |  |
| HAZEAN2\|DNA/hAT-Ac | | 394023 | | 394640 |  | NON | DNA | CCTCCAAG |  |  |
| hAT-N18_ZM\|DNA/hAT-Ac | | 439347 | | 440120 |  | NON | DNA | GGTGCAAC |  |  |
| 1- *O.sativa* L. ssp. *japonica* | |  |  |  |  |  |  |  |  |  |
| 2- *O. sativa* L. ssp. *indica* | |  |  |  |  |  |  |  |  |  |
| 3- *O. glaberrima* | |  |  |  |  |  |  |  |  |  |
| 4- *O. rufipogon* | |  |  |  |  |  |  |  |  |  |
| 5- *O. nivara* | |  |  |  |  |  |  |  |  |  |
| 6- *O. glumaepatula* | |  |  |  |  |  |  |  |  |  |
