# Supplementary material for: Comparative Sequence Analysis of the Ghd7 Orthologous Regions Revealed Movement of Ghd7 in the Grass Genomes
Source: PLoS One. 2012 Nov 21;7(11):e50236. doi: 10.1371/journal.pone.0050236 (PMC3503983; doi:10.1371/journal.pone.0050236)
Supplement: Table S12 — Comparison of Gypsy and Copia content in the Ghd7 , Adh1 and Hd1 regions. (DOCX) [file pone.0050236.s016.docx]

**Table S12** Comparison of *Gypsy* and *Copia* content in the *Ghd7*, *Adh1* and *Hd1* regions.

|  | *Ghd7* |  | *Adh1* |  | *Hd1* |  |
| --- | --- | --- | --- | --- | --- | --- |
|  | *Copia* (%) | *Gypsy* (%) | *Copia* (%) | *Gypsy* (%) | *Copia* (%) | *Gypsy* (%) |
| *O. sativa* L. ssp*. japonica* (AA) | 7.39 | 23.37 | 4.8 | 4.6 | 0 | 8.29 |
| *O. sativa* L. ssp*. indica* (AA) | 7.67 | 24.04 | 6 | 9.9 | 0 | 20.53 |
| *O. glaberrima* (AA) | 6.4 | 21.9 | 6.1 | 0 | 0.21 | 8.67 |
| *O. glumaepatula* (AA)^a^ | 4.24 | 33.83 | * | * | * | * |
| *O. rufipogon* (AA) | 8.51 | 44.69 | 5.7 | 0 | 1.17 | 12.11 |
| *O. nivara* (AA) | 6.45 | 45.52 | 3.2 | 0 | 0.2 | 0 |
| *O. puctata* (BB) | 6 | 51.22 | 0 | 2 | 3.55 | 8.15 |
| *O. officinalis* (CC) | 5.5 | 26.91 | 2 | 11.8 | 3.1 | 13.49 |
| *O. australiensis* (EE) | 10.54 | 35.72 | 16 | 11.4 | 6.2 | 56.99 |
| *O. brachyantha* (FF) | 8.47 | 12.39 | 9.9 | 0.01 | 0.07 | 3.08 |
| *B. distachyon* | 5.06 | 0.12 |  |  |  |  |
| *S. bicolor* | 7.97 | 20.44 |  |  |  |  |
| *Z. mays* | 36.89 | 35.38 |  |  |  |  |
| ^a^This species is not included in *Adh1* and *Hd1* sequence dataset. | | | | | | |
