# Supplementary material for: Comparative Sequence Analysis of the Ghd7 Orthologous Regions Revealed Movement of Ghd7 in the Grass Genomes
Source: PLoS One. 2012 Nov 21;7(11):e50236. doi: 10.1371/journal.pone.0050236 (PMC3503983; doi:10.1371/journal.pone.0050236)
Supplement: Table S13 — Comparison of gene densities and TE contents in the Ghd7 , Adh1 , Hd1 and Moc1 regions. (DOCX) [file pone.0050236.s017.docx]

**Table S13** Comparison of gene densities and TE contents in the *Ghd7*, *Adh1*, *Hd1* and *Moc1* regions.

|  | Gene Density (kb/gene) | | | | TE content (%) | | | |
| --- | --- | --- | --- | --- | --- | --- | --- | --- |
|  | *Ghd7* | *Adh1* | *Hd1* | *Moc1* | *Ghd7* | *Adh1* | *Hd1* | *Moc1*^b^ |
| *O.* *sativa* L. ssp. *japonica* (AA) | 29.11 | 13.3 | 12 | 8.4 | 48.99 | 41.2 | 40.3 |  |
| *O.* *sativa* L. ssp. *indica* (AA) | 26.4 | 16.6 | 13.7 | 7.2 | 44.89 | 53.7 | 44.3 |  |
| *O.* *nivara* (AA) | 49.46 | 10.7 | 17.1 | 7.8 | 66.76 | 47.6 | 39.4 |  |
| *O. rufipogon* (AA) | 32.26 | 12.9 | 10.2 | 7.9 | 65.93 | 48.6 | 38.7 |  |
| *O.* *glaberrima* (AA) | 27.08 | 10 | 15.1 | 7.5 | 43.01 | 39 | 37.6 |  |
| *O. glumaepatula* (AA)^a^ | 33.81 | * | * | * | 53.28 | * | * |  |
| *O. punctata* (BB) | 60.54 | 11.6 | 12 | 11.7 | 70.06 | 46.5 | 29.8 |  |
| *O.* *officinalis* (CC) | 110.24 | 14.9 | 16.8 | 11.4 | 61.28 | 56.4 | 50.7 |  |
| *O. australiensis* (EE) | 106.37 | 27.4 | 44.4 | 23.6 | 60.03 | 58.8 | 80.6 |  |
| *O.* *brachyantha* (FF) | 16.76 | 10.7 | 9.4 | 8.8 | 29.5 | 28.3 | 27.7 |  |
| Average | 49.2 | 14.23 | 16.74 | 10.48 | 54.37 | 46.68 | 43.23 | 29.5 |
| ^a^This species is not included in the other 3 gene regions. There is a gap between the four BAC which were selected in this species. So the actual results are more than the values. | | | | | | | | |
| ^b^This region was just counted the number of TE from every species of *Oryza* genus and average TE content. | | | | | | | | |
