# Supplementary material for: Comparative Sequence Analysis of the Ghd7 Orthologous Regions Revealed Movement of Ghd7 in the Grass Genomes
Source: PLoS One. 2012 Nov 21;7(11):e50236. doi: 10.1371/journal.pone.0050236 (PMC3503983; doi:10.1371/journal.pone.0050236)
Supplement: Table S14 — Accession number of CCT family genes from rice, B. distachyon , S. bicolor and Z. mays . (DOCX) [file pone.0050236.s018.docx]

**Table S14** Accession number of CCT family genes from rice, *B. distachyon*, *S. bicolor* and *Z. mays*.

| *O. sativa* L. ssp. *japonica* | *B. distachyon* | *S. bicolor* | *Z. mays* (Chromosome No.) |
| --- | --- | --- | --- |
| LOC_Os07g15770 | Bradi3g33340 | Sb01g029080 | GRMZM2G004483_P01 (9) |
| LOC_Os10g41100 | Bradi3g10010 | Sb06g000570 | GRMZM2G381691_P01 (10) |
| LOC_Os04g42020 | Bradi5g14600 | Sb06g021480 | GRMZM2G095598_P01 (2) |
| LOC_Os02g39710 | Bradi3g48450 | Sb04g025660 | GRMZM2G021777_P01 (5) |
| LOC_Os06g44450 | Bradi3g05800 | Sb10g026060 | AC233888.1_FGP002 (10) |
| LOC_Os02g08150 | Bradi1g31280 | Sb04g005250 | GRMZM2G148772_P01 (5) |
|  |  | Sb07g008550 | GRMZM2G038783_P01 (9) |
|  |  |  | GRMZM2G176173_P01 (4) |
|  |  |  | GRMZM2G092363_P01 (10) |
