# Supplementary material for: Comparative Sequence Analysis of the Ghd7 Orthologous Regions Revealed Movement of Ghd7 in the Grass Genomes
Source: PLoS One. 2012 Nov 21;7(11):e50236. doi: 10.1371/journal.pone.0050236 (PMC3503983; doi:10.1371/journal.pone.0050236)
Supplement: Table S15 — Chromosomal location and sequence size of Ghd7 -related genes. (DOCX) [file pone.0050236.s019.docx]

**Table S15** Chromosomal location and sequence size of *Ghd7*-related genes.

| Gene region | Orthologous regions in the four species | | | | |
| --- | --- | --- | --- | --- | --- |
|  |  | *O. sativa* L. ssp. *japonica* | *B. distachyon* | *S. bicolor* | *Z. mays* |
| *Ghd7* | Chromosome No. | chr7 | chr 1 | chr 2 | chr 2 |
|  | Position | 8851000_9404000 | 51390000_51531100 | 9880000_12000000 | 167800000_168300000 |
| Bradi3g10010 | Chromosome No. | chr 2 | chr 3 | chr 4 | chr 5 |
|  | Position | 9400000_9900000 | 7927490_8417490 | 12450000_14000000 | 134020000_137620000 |
| Sb06g000570 | Chromosome No. | chr 4 | chr 5 | chr 6 | chr 10 |
| GRMZM2G381691 | Position | 170000_649000 | 2150000_2500000 | 920000_6420000 | 93830000_94330000 |
| *O. brachyantha* (FF) sequence from unpublished data. | | | | | |
